# Antithrombotic strategies and DOAC dosing following left atrial appendage occlusion: a network meta-analysis

**DOI:** 10.1093/ehjcvp/pvaf078

**Published:** 2025-11-25

**Authors:** Athanasios Samaras, Paschalis Karakasis, Athanasios Feidakis, George Giannakoulas, Nikolaos Fragakis, Jens-Erik Nielsen-Kudsk, Xavier Freixa, Devi G Nair, James V Freeman, Martin Bergmann, Ulf Landmesser, Apostolos Tzikas

**Affiliations:** School of Medicine, Faculty of Health Sciences, Aristotle University of Thessaloniki, 54124 Thessaloniki, Greece; Second Department of Cardiology, General Hospital ‘Hippokration’, 54642 Thessaloniki, Greece; Second Department of Cardiology, General Hospital ‘Hippokration’, 54642 Thessaloniki, Greece; Department of Cardiology, Heart Center, University of Cologne, 50937 Cologne, Germany; First Department of Cardiology, AHEPA University Hospital of Thessaloniki, 54636 Thessaloniki, Greece; Second Department of Cardiology, General Hospital ‘Hippokration’, 54642 Thessaloniki, Greece; Department of Cardiology, Aarhus University Hospital, 8200 Aarhus, Denmark; Department of Cardiology, Institut Cardiovascular, IDIBAPS, Hospital Clinic of Barcelona, 08036 Barcelona, Spain; Department of Cardiac Electrophysiology, St Bernard's Heart and Vascular Center, Jonesboro, AR 72401, USA; Section of Cardiovascular Medicine, Yale University School of Medicine, New Haven, CT 06511, USA; Center for Outcomes Research and Evaluation, Yale-New Haven Hospital, New Haven, CT 06511, USA; Department of Cardiology and Intensive Care Medicine, Asklepios Klinik Altona, 22763 Hamburg, Germany; Charité-University Medicine Berlin Corporate Member of Free University Berlin and Humboldt-University Berlin, 10117 Berlin, Germany; German Centre for Cardiovascular Research (DZHK), Partner Site Berlin, 10785 Berlin, Germany; Department of Cardiology, Angiology and Intensive Care Medicine, Deutsches Herzzentrum Charité, Campus Benjamin Franklin, 13353 Berlin, Germany; Berlin Institute of Health, Charité-University Medicine Berlin, 13353 Berlin, Germany; School of Medicine, Faculty of Health Sciences, Aristotle University of Thessaloniki, 54124 Thessaloniki, Greece; Second Department of Cardiology, General Hospital ‘Hippokration’, 54642 Thessaloniki, Greece; European Interbalkan Medical Center, 57001 Thessaloniki, Greece

**Keywords:** Left atrial appendage occlusion, Antithrombotic strategies, Direct oral anticoagulants, DOAC dosing, Outcomes

## Abstract

**Aims:**

The optimal short-term antithrombotic strategy following left atrial appendage occlusion (LAAO) remains uncertain, with the need to balance thromboembolic prevention and bleeding risk presenting a critical challenge. Recent evidence suggests that direct oral anticoagulants (DOACs) may provide a favourable safety–efficacy profile, with low-dose regimens showing potential benefits during the device endothelialization period. This network meta-analysis (NMA) aimed to compare the efficacy and safety of various antithrombotic strategies, including DOAC dosing, following LAAO.

**Methods and results:**

A systematic review and NMA were conducted following Cochrane and PRISMA guidelines. Eligible studies included randomized controlled trials (RCT) and observational studies comparing at least two antithrombotic regimens in patients with non-valvular atrial fibrillation undergoing percutaneous LAAO. Primary outcomes were major bleeding and thromboembolism. Secondary outcomes included device-related thrombosis (DRT) and all-cause mortality. Pairwise and network meta-analyses were performed using a random-effects model. A total of 52 studies (49 observational and 3 RCTs) involving 69 751 patients were included. DOACs were consistently associated with significantly lower rates of major bleeding and all-cause mortality than other antithrombotic regimens. Low-dose DOACs showed a potential advantage over standard-dose DOACs in reducing major bleeding risk (odds ratio 0.45, 95% confidence interval: 0.22–0.92). For thromboembolism and DRT, standard-dose DOAC significantly reduced risk compared with single antiplatelet therapy (SAPT) but not with dual antiplatelet therapy (DAPT), whereas low-dose DOAC significantly reduced both outcomes compared with SAPT, DAPT, and vitamin K antagonists plus SAPT. In ranking analysis, DOACs emerged as the most effective and safest antithrombotic strategy, with low-dose DOACs demonstrating further safety benefits in bleeding outcomes.

**Conclusion:**

DOACs provide a superior safety–efficacy profile compared with other antithrombotic strategies following LAAO, significantly reducing the risks of major bleeding, thromboembolic events, and mortality. While low-dose DOACs may offer additional bleeding risk reduction without compromising efficacy, further research is warranted to confirm their role in clinical practice.

## Graphical Abstract

**Graphical Abstract:**
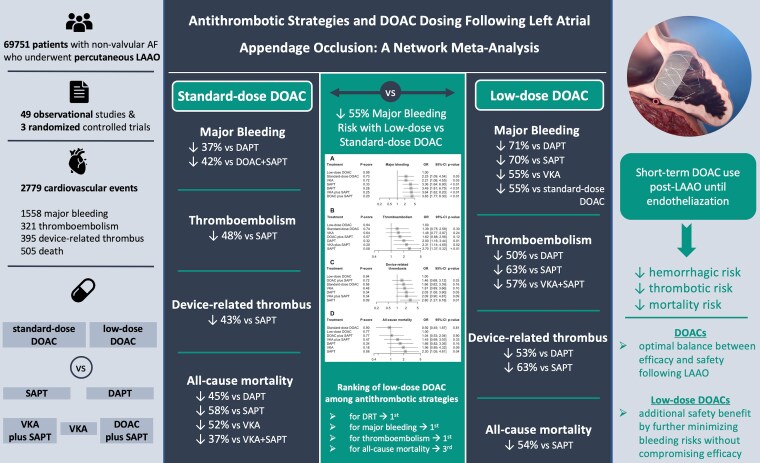

## Introduction

Amidst the evolution of left atrial appendage occlusion (LAAO) as an effective strategy for stroke prevention in atrial fibrillation (AF), the optimal post-procedural antithrombotic regimen remains a subject of uncertainty and ongoing debate.^1^ The Achilles’ heel of LAAO is the development of device-related thrombosis (DRT) after successful device implantation, particularly during the critical endothelization period.^2^ Despite procedural advancements, DRT may occur in 2%–5% of cases, and even up to 1–2 years after implant, and it has been linked to thromboembolic events.^3^ Another important limitation is the risk of incomplete occlusion, which may compromise long-term outcomes.^4^ The choice of short-term therapy after LAAO is therefore especially challenging, as it must carefully reduce the thrombotic risk without increasing the risk for major bleeding, which is the most frequent serious adverse event following LAAO.^5^

Currently, there is no consensus regarding the optimal short-term post-procedural antithrombotic therapy.^6^ Hence, treatment strategies vary considerably across studies, regions, and clinical practices, each carrying a distinct risk-benefit profile.^7^ In Europe, short-term dual antiplatelet therapy (DAPT) followed by lifelong single antiplatelet therapy (SAPT) is commonly recommended, whereas in the USA, standard-dose oral anticoagulation (OAC) with or without aspirin for at least 45 days remains the approved strategy, followed by antiplatelet therapy if imaging confirms satisfactory device sealing.^8^ Lately, low-dose direct oral anticoagulation (DOAC) has gained interest, showing promising results in achieving an optimal balance between thrombotic and haemorrhagic risks.^9^ However, its use should still be considered off-label as preliminary data regarding its use after LAAO remains limited.^10^

This study aims to evaluate compare the efficacy and safety of different antithrombotic strategies, including DOAC dosing, following LAAO based on a network meta-analysis (NMA).

## Methods

This systematic review and NMA were performed and reported following the Cochrane Collaboration Handbook for Systematic Reviews of Interventions and the Preferred Reporting Items for Systematic Reviews and Meta-analysis statement guidelines (see Supplementary material online, *Tables S1* and *S2*). The protocol of this meta-analysis has been prospectively registered at the Open Science Framework registries (10.17605/OSF.IO/5MUX6).

### Data source and search strategy

Literature searches were performed at the PubMed/Medline, EMBASE, Cochrane, and Scopus databases, from inception through the final search date of 15 September 2024. Two authors performed a systematic review, and disagreements were resolved through consensus discussions, involving the senior author for final adjudication. Study selection involved screening titles and abstracts followed by a full-text evaluation of potentially eligible studies. The detailed search strategy for each database is presented in Supplementary material online, *Table S3*.

### Eligibility criteria

There was no restriction concerning the publication date, status, or language. We considered studies eligible for inclusion if they (i) were randomized controlled trials (RCTs) or observational studies; (ii) compared at least two antithrombotic regimens; (iii) enrolled patients with nonvalvular AF who underwent LAAO with the use of endocardial devices; and (iv) presented data regarding any of the prespecified efficacy and safety outcomes. We excluded studies that (i) did not report any of the outcomes of interest; (ii) analysed solely epicardial devices; (iii) analysed LAAO in conjunction with cardiac surgery; (iv) only reported periprocedural endpoints; (v) reported endpoints based on antithrombotic therapy at the time of outcome rather than therapy prescribed at discharge; or (vi) were editorials, letters, conference abstracts, case reports, or case series. In the case of overlapping populations, only the study with the largest sample size was included, unless different studies offered different outcomes.

For studies with overlapping populations, only the report with the largest sample size or most comprehensive follow-up was included to avoid double-counting of patients. Smaller companion publications from the same registry or trial were excluded to maintain independence of datasets, although this may have led to omission of some outcome details.

### Definitions of direct oral anticoagulant dosing

Low-dose DOAC was defined as apixaban 2.5 mg twice daily, rivaroxaban 10–15 mg once daily, dabigatran 110 mg twice daily, or edoxaban 30 mg once daily. Standard-dose DOAC was defined as apixaban 5 mg twice daily, rivaroxaban 20 mg once daily, dabigatran 150 mg twice daily, or edoxaban 60 mg once daily.

### Outcomes

The primary safety outcome was major bleeding, according to the study definition. The primary efficacy outcome was thromboembolism as a composite of stroke, transient ischaemic attack, or systemic embolism. Secondary endpoints included (i) DRT, defined as a thrombus attached to the atrial surface of the device as seen on transoesophageal echocardiography or cardiac computed tomography and (ii) all-cause mortality. For the assessment of major bleeding and thromboembolism, the definitions utilized were those specified in the respective primary studies (see Supplementary material online, *Table S4*). We limited our analysis to postprocedural endpoints and excluded periprocedural endpoints in studies where they were distinguished. For studies reporting multiple follow-up periods, we selected the earliest available post-discharge follow-up to best capture the temporal proximity and likely causality between the prescribed antithrombotic strategy at discharge and early adverse events. This approach aims to isolate the effect of initial post-procedural therapy, particularly during the device endothelialization phase, which is the highest-risk period for DRT and bleeding. Moreover, longer-term outcomes were reported inconsistently and heterogeneously and were therefore not synthesized in the primary analysis.

### Quality assessment

Quality assessment of RCTs was conducted using Cochrane’s Risk of Bias 2 tool for randomized studies and the Risk of Bias in Non-randomized Studies of Interventions tool for observational studies. The risk of bias assessment was performed independently by 2 authors, and disagreements were resolved by consensus between them. We explored the potential for publication bias by visual inspection of the comparison-adjusted funnel plots and by Egger’s test.

Certainty of evidence for each pairwise comparison was assessed using the Confidence in Network Meta-Analysis (CINeMA) framework, which evaluates six domains: within-study bias, across-studies bias, indirectness, imprecision, heterogeneity, and incoherence. Each domain was rated as having no, some, or major concerns, and translated into an overall confidence level (high, moderate, low, very low). CINeMA integrates judgments from risk of bias tools (ROBINS-I for observational studies and RoB2 for randomized trials) together with considerations of network structure and precision.

### Sensitivity analyses

Subgroup analyses were conducted, by restricting the follow-up periods to 3 and 6 months, to assess the impact of follow-up time on the primary efficacy and safety endpoints. To address potential biases arising from heterogeneous study designs, a sensitivity analysis confined to observational studies was conducted. Given the limited number of RCTs (*n* = 3), no separate sensitivity analysis was performed for this subgroup, as the paucity of data precluded a robust and meaningful synthesis, particularly within the framework of a network meta-analysis.

### Statistical analysis

Transitivity is a critical assumption in network meta-analysis. In our context, where the network included predominantly observational studies alongside a few randomized trials, we could not assume joint randomization. Instead, we assessed transitivity by evaluating whether potential effect modifiers were sufficiently similar across treatment comparisons to permit valid indirect inferences. Specifically, we reviewed the dataset of eligible studies for discrepancies in predefined effect modifiers, including average patient age, sex distribution, baseline risk factors (CHA_2_DS_2_-VASc and HAS-BLED scores where available), device type, timing of first imaging for device-related thrombus, duration of intervention, and follow-up period. Despite variability, the populations could reasonably be considered exchangeable for analysis.

Network diagrams were developed for each outcome to visually represent the interventions directly compared in the included studies. We attempted to use multivariable adjusted data to account for confounding whenever available; however, only one study reported adjusted outcomes stratified by antithrombotic regimen, and therefore our analyses relied on crude event counts. Direct pairwise effect estimates were expressed as odds ratios (ORs) with 95% confidence intervals (CIs), calculated using random-effects DerSimonian–Laird models. Both direct and indirect evidence was synthesized using a random-effects frequentist NMA on the log-odds ratio scale. Results are presented as odds ratios with 95% CIs.

The overall homogeneity of the network was evaluated using Cochran’s *Q* test, while between-study variance was measured with τ². Any τ^2^ values under 0.25 were considered acceptable heterogeneity; values between 0.25 and 1.0 represented moderately high heterogeneity, and values greater than 1.0 represented very high heterogeneity. Statistical inconsistency was examined on a global scale using the design-by-treatment interaction test. We considered evidence of inconsistency if *P*-values were less than 0.05. To assess the presence of small-study effects, including potential publication bias, comparison-adjusted funnel plots were utilized for visual inspection, and the Egger’s test was employed for formal evaluation. Because regimen-specific adjusted estimates were rarely reported, the analyses were based on crude event counts as provided in each study. Consequently, potential confounding could not be directly adjusted for in the synthesis.

We estimated the ranking probabilities of the different antithrombotic regimens based on *P*-scores. *P*-score is measured on a scale from 0 (worst) to 1 (best), with a higher score indicating the better overall performance of the competing treatment.

We used R version 4.2.2 and the extension packages ‘netmeta’ and ‘dmetar’, for all calculations and almost all graphics. Stata version 14.2 (StataCorp LLC) was used to create network plots.

## Results

### Study selection and characteristics

Our systematic search identified 2841 potential articles, which underwent full-text review (see Supplementary material online, *Figure S1*). Ultimately, 49 observational studies and 3 RCTs were included, totalling 69 751 patients with nonvalvular AF and 2779 cardiovascular events in the network analysis. Study design, population, antithrombotic regimens, results, and other characteristics of the included studies are provided in Supplementary material online, *Tables S5*–S7. Thirteen of these studies reported data regarding a total of 83 events in 1454 patients that received low-dose DOACs. In the pooled population, the mean age was 73 years, and 59% of patients were men. Mean CHA_2_DS_2_-VASc and HAS-BLED scores were 4.2 and 3.2, respectively. Of the 45 studies evaluating the presence of DRT, most of them (*n* = 33) reported a follow-up duration of 1 to 3 months. The average follow-up duration for thromboembolism, major bleeding, and mortality was approximately 17 months.

The specific time frames of antithrombotic therapies used in each study are presented in Supplementary material online, *Table S6*. The duration of OAC therapy following LAAO generally lasted 45 to 90 days. After this period, OAC was discontinued, and patients were typically transitioned to DAPT for a duration varying between 1 and 3 months. Subsequently, most patients were maintained on SAPT for 3 to 6 months, with some transitioning to indefinite antiplatelet monotherapy. In certain cases, antithrombotic therapy was completely discontinued after a period of SAPT treatment.

### Antithrombotic strategies post-left atrial appendage occlusion

**Figure 1:**
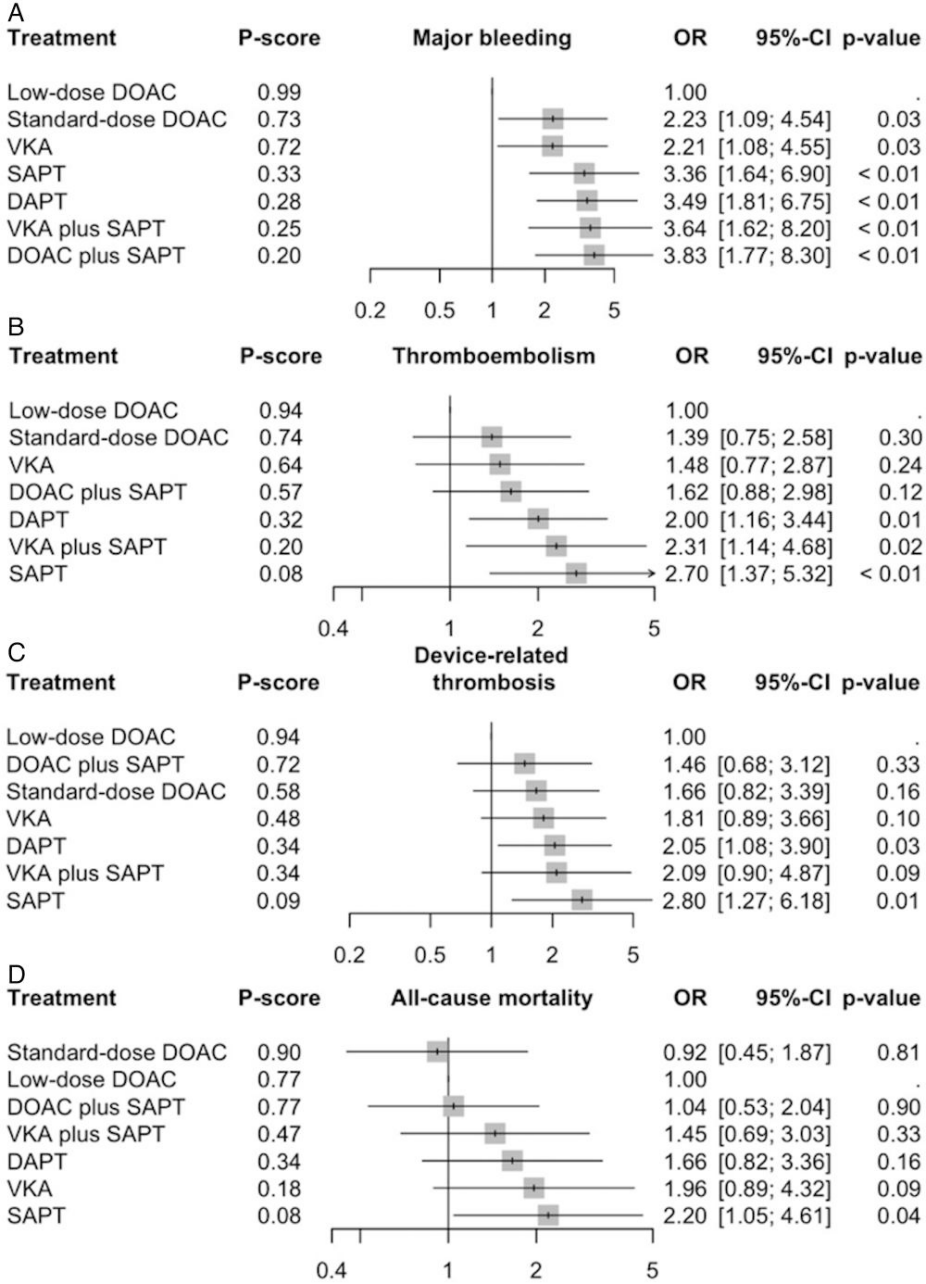
Forest plots of random effects network meta-analyses for device-related thrombosis (*A*), major bleeding (*B*), thromboembolic events (*C*), and all-cause mortality (*D*), sorted by descending P-score. Low-dose was used as a statistical reference for display purposes. 95% CI, 95% confidence interval; DAPT, dual antiplatelet therapy; DOAC, direct oral anticoagulants; OR, odds ratio; SAPT, single antiplatelet therapy; VKA, vitamin K antagonist.

Overall, seven antithrombotic strategies used in each outcome analysis, including (i) low-dose DOAC; (ii) standard-dose DOAC; (iii) SAPT; (iv) DAPT; (v) vitamin K antagonists (VKA); (vi) DOAC plus SAPT; and (vii) VKA plus SAPT. All antithrombotic therapies were directly compared with at least 1 other. We set ‘low-dose DOAC’ as the reference comparator. The network estimates for each outcome of interest are presented in *Figure 1*, with low-dose DOAC used as the reference.

Among studies reporting DOAC use, apixaban was the most frequently used agent, followed by rivaroxaban and dabigatran. Low-dose regimens most often involved apixaban 2.5 mg twice daily or rivaroxaban 15 mg once daily, whereas dabigatran 110 mg twice daily was reported in smaller cohorts.

### Major bleeding

Major bleeding was reported in 39 studies, encompassing 64 930 patients who collectively experienced 1558 events (2.4%).

**Figure 2:**
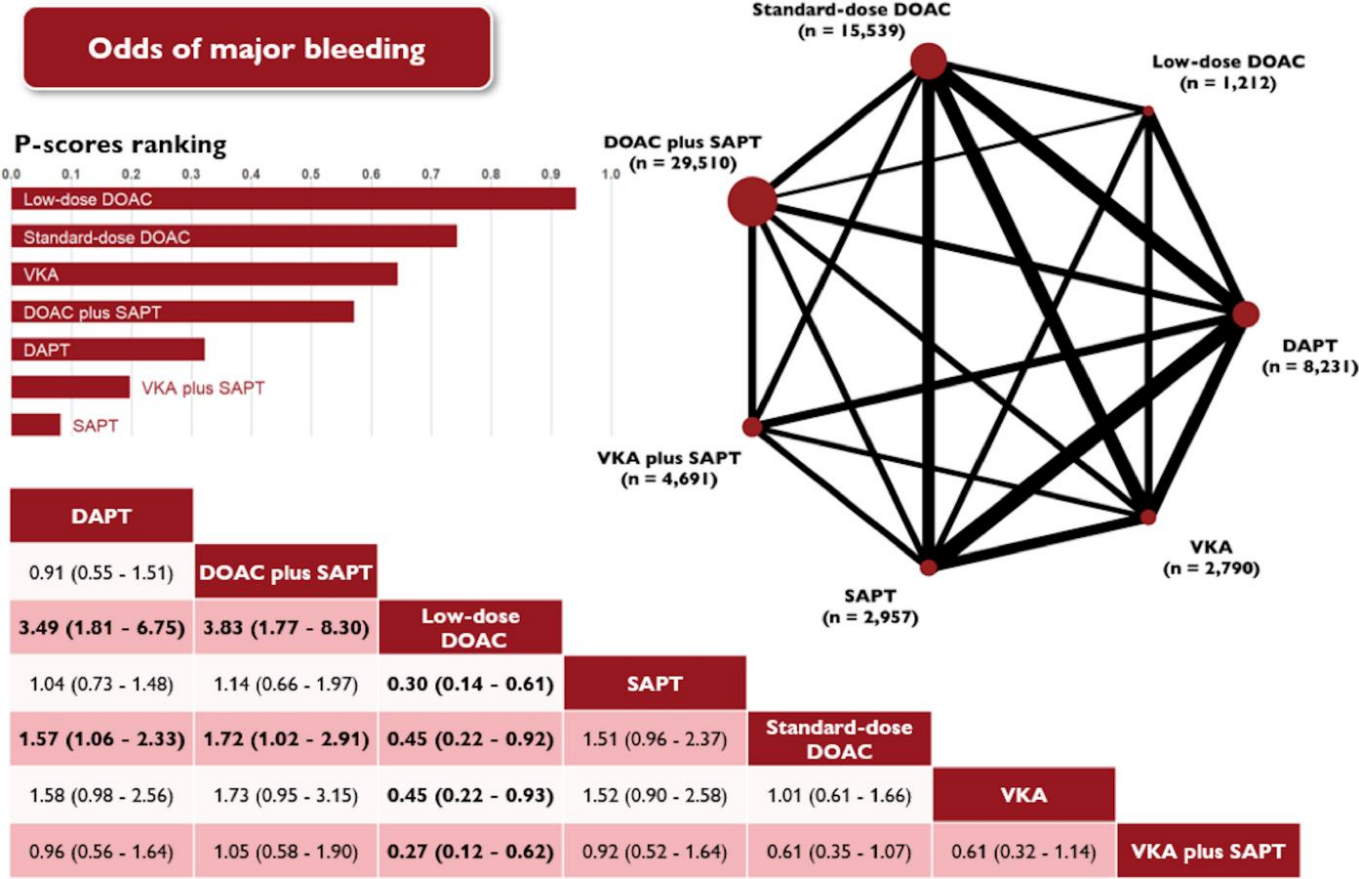
Network plot (top right) displaying the efficacy of different antithrombotic therapies prescribed at discharge and the odds of major bleeding following left atrial appendage occlusion. In the plot, each node represents a specific antithrombotic strategy, while the edges indicate direct comparisons made between these strategies in the included trials. The size of each node reflects the number of patients allocated to that treatment, and the thickness of each edge corresponds to the frequency of direct comparisons between the strategies. The accompanying league table (bottom) presents antithrombotic therapies in alphabetical order, showing network odds ratios and 95% confidence intervals for pairwise comparisons between the column- and row-defining treatments. Odds ratios less than 1 indicate a benefit for the column-defining therapy. Estimates with a two-tailed *P*-value <0.05 were considered statistically significant and are presented in bold. The bar plot (top left) illustrates P-scores, which are measured on a scale ranging from 0 (worst) to 1 (best), with higher values indicating superior overall effectiveness of the competing treatments. DAPT, dual antiplatelet therapy; DOAC, direct oral anticoagulants; SAPT, single antiplatelet therapy; VKA, vitamin K antagonist.

Standard- and low-dose DOACs were consistently associated with significantly lower rates of major bleeding than all other antithrombotic regimens (*Figure 2* and Supplementary material online, *Figure S2*). In particular, standard-dose DOACs significantly reduced the risk of major bleeding compared with DAPT (OR 0.63; 95% CI: 0.43–0.94), and DOAC plus SAPT (OR 0.58; 95% CI: 0.34–0.98). Low-dose DOACs significantly reduced the risk of major bleeding compared with SAPT (OR 0.30; 95% CI: 0.14–0.61), DAPT (OR 0.29; 95% CI: 0.15–0.55), VKAs (OR 0.45; 95% CI: 0.22–0.93), and standard-dose DOACs (OR 0.45; 95% CI: 0.22–0.92).

### Thromboembolism

Thromboembolic events were reported in 40 studies that enrolled 63 033 patients incurring a combined total of 321 events (0.5%).

**Figure 3:**
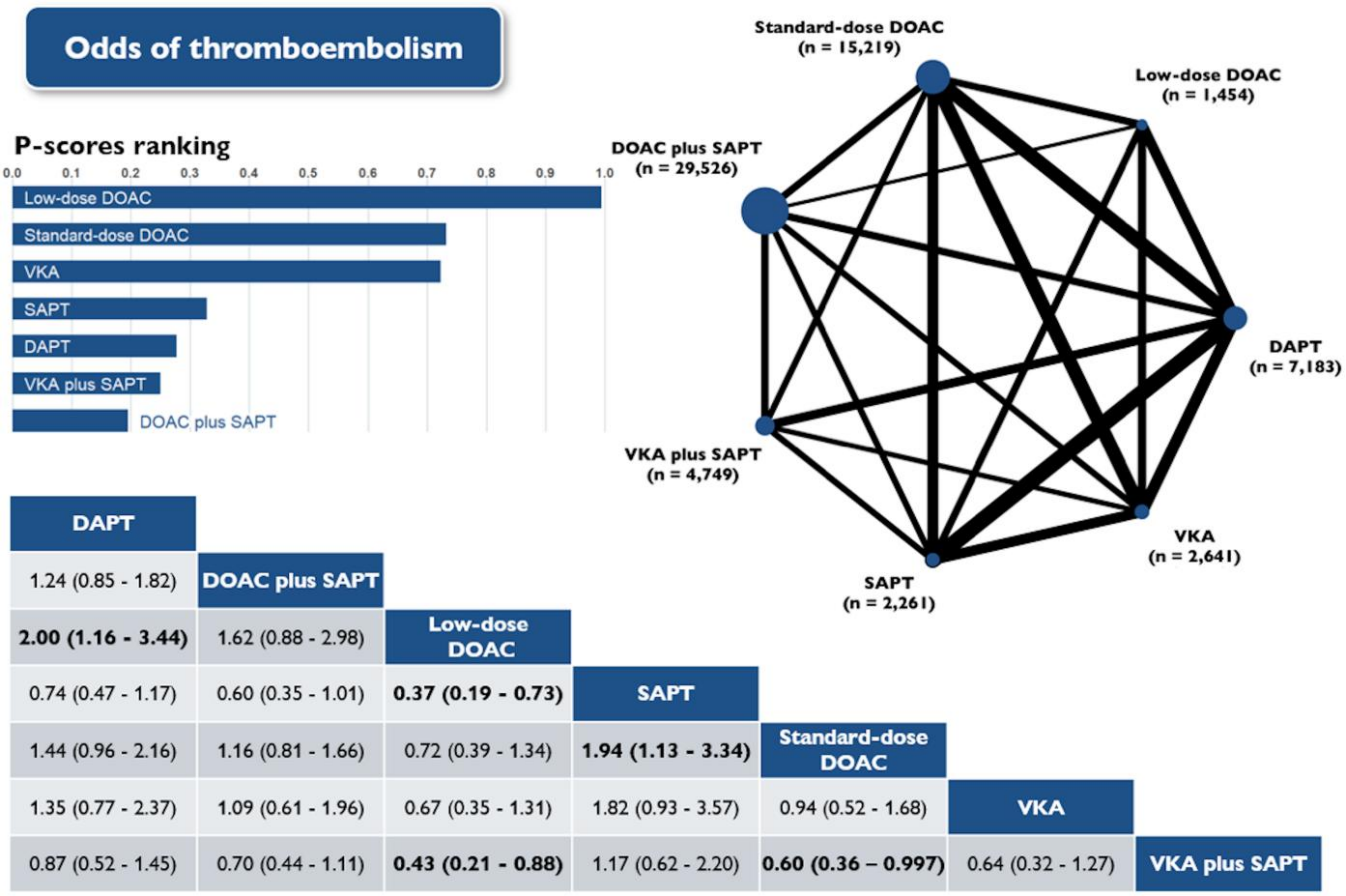
Network plot, league table and P-scores presenting estimates from the network random-effects meta-analysis for the association between different antithrombotic therapies and the odds of thromboembolism in patients who have undergone left atrial appendage occlusion. Network odds ratios and 95% confidence intervals for pairwise comparisons between the column- and row-defining treatments are presented. DAPT, dual antiplatelet therapy; DOAC, direct oral anticoagulants; SAPT, single antiplatelet therapy; VKA, vitamin K antagonist.

Standard-dose DOACs significantly reduced the risk of thromboembolism compared with SAPT (OR 0.52; 95% CI: 0.30–0.88). Low-dose DOACs significantly reduced the risk of thromboembolism compared with SAPT (RR 0.37; 95% CI: 0.19–0.73), DAPT (OR 0.50; 95% CI: 0.29–0.86), and VKA plus SAPT (OR 0.43; 95% CI: 0.21–0.88, *Figure 3* and Supplementary material online, *Figure S3*). DOACs (irrespective of dosing) also showed potential reduction of thromboembolic risk compared with the rest of the antithrombotic regimens and patterns, but these results were not statistically significant.

### Device-related thrombosis

The occurrence of DRT was reported in 46 studies, encompassing 61 588 patients who collectively experienced 395 events (0.6%).

**Figure 4:**
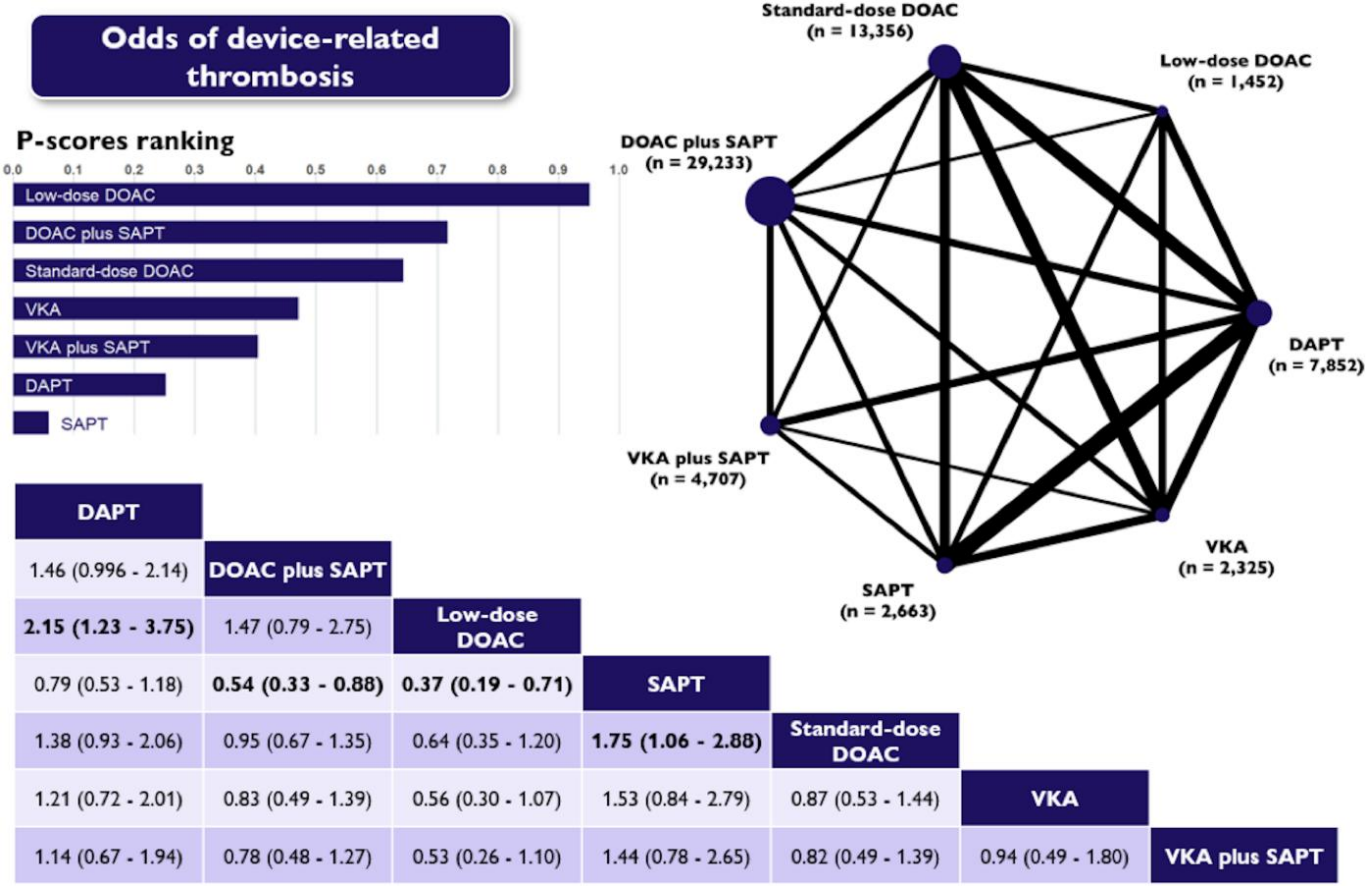
Network plot, league table and P-scores presenting estimates from the network random-effects meta-analysis for the association between different antithrombotic therapies and the odds of DRT in patients who have undergone left atrial appendage occlusion. Network odds ratios and 95% confidence intervals for pairwise comparisons between the column- and row-defining treatments are presented. DAPT, dual antiplatelet therapy; DOAC, direct oral anticoagulants; SAPT, single antiplatelet therapy; VKA, vitamin K antagonist.

Standard-dose DOACs significantly reduced the risk of DRT compared with SAPT (OR 0.57; 95% CI: 0.38–0.94). When compared with SAPT and DAPT, low-dose DOACs significantly reduced the risk of DRT, with odds ratios of 0.37 (95% CI: 0.19–0.71) and 0.47 (95% CI: 0.27–0.82), respectively (*Figure 4* and Supplementary material online, *Figure S4*). In general, both standard- and low-dose DOACs consistently appeared to be more effective than the rest of antithrombotic strategies, even though results were not statistically significant.

### All-cause mortality

All-cause mortality was reported in 23 studies that enrolled 59 641 patients, with a combined total of 505 events (0.8%).

**Figure 5:**
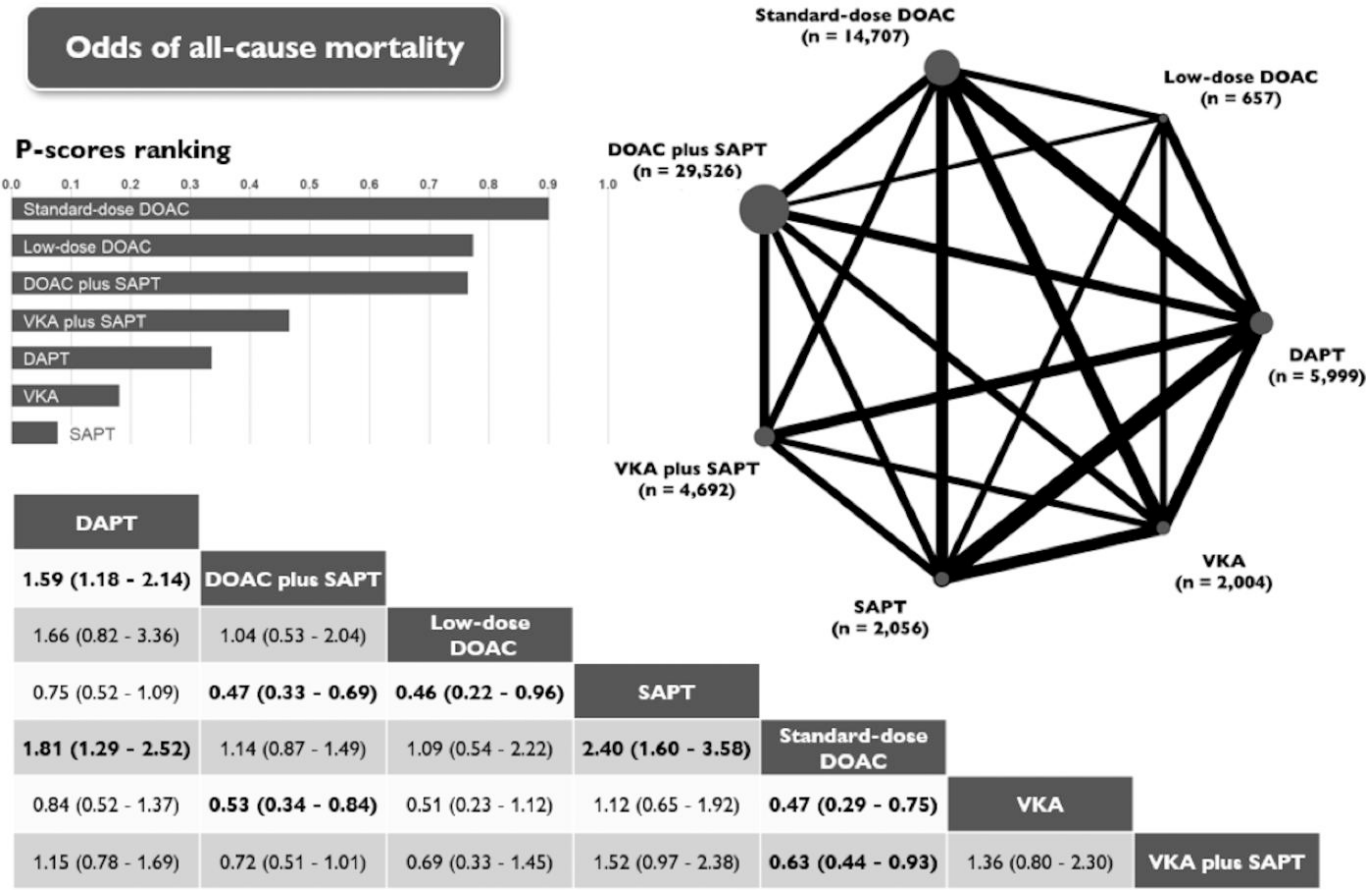
Network plot, league table and P-scores presenting estimates from the network random-effects meta-analysis for the association between different antithrombotic therapies and the odds of all-cause mortality in patients who have undergone left atrial appendage occlusion. Network odds ratios and 95% confidence intervals for pairwise comparisons between the column- and row-defining treatments are presented. DAPT, dual antiplatelet therapy; DOAC, direct oral anticoagulants; SAPT, single antiplatelet therapy; VKA, vitamin K antagonist.

Standard-dose DOACs significantly reduced the risk of mortality compared with SAPT (OR 0.42; 95% CI: 0.28–0.63), DAPT (OR 0.55; 95% CI: 0.40–0.77), VKAs (OR 0.48; 95% CI: 0.29–0.77), and VKA plus SAPT (OR 0.63; 95% CI: 0.43–0.93). Compared with SAPT (OR 0.46; 95% CI: 0.22–0.96), low-dose DOACs showed significant reduction in mortality risk (*Figure 5* and Supplementary material online, *Figure S5*). Comparisons with other antithrombotic schemes revealed neutral results.

### Ranking of antithrombotic strategies

*Table 1* and Supplementary material online, *Figures S6*–S9 present *P*-score values and ranking probabilities for efficacy and safety endpoints.

**Table 1:** *P*-Scores for each antithrombotic strategy and endpoints of interest

Antithrombotic strategy	Device-related thrombosis	Major bleeding	Thromboembolic events	All-cause mortality
DAPT	0.2514	0.3225	0.2773	0.3358
DOAC plus SAPT	0.7171	0.5712	0.1950	0.7656
Low-dose DOAC	**0.9525**	**0.9415**	**0.9948**	0.7738
SAPT	0.0588	0.0817	0.3279	0.0768
Standard-dose DOAC	0.6440	0.7427	0.7323	**0.9014**
VKA	0.4714	0.6438	0.7228	0.1808
VKA plus SAPT	0.4047	0.1967	0.2500	0.4658

The highest *P*-scores are presented in bold.

DAPT, dual antiplatelet therapy; DOAC, direct oral anticoagulants; SAPT, single antiplatelet therapy; VKA, vitamin K antagonist.

Based on *P*-scores, DOACs consistently emerged as the top-ranking antithrombotic strategy, particularly for balancing both efficacy and safety in LAAO patients, with low-dose appearing to have a potential advantage. Low-dose DOACs achieved the highest *P*-scores for prevention of major bleeding (0.9415), thromboembolism (0.9948), and DRT (0.9525) indicating this treatment to be the most effective at reducing both haemorrhaging and thrombotic risk. While low-dose DOACs ranked second for all-cause mortality (0.7738), below standard-dose DOACs, they still outperformed antiplatelet therapies and VKAs.

### Network consistency and heterogeneity

All fitted models converged relatively well, and we did not find statistical evidence of inconsistency in our NMA across each outcome. For major bleeding and thromboembolism, *I*² values ranged from 34% to 62%, indicating moderate heterogeneity, while τ² values remained <0.25 for most comparisons. For DRT and all-cause mortality, both *I*² and τ² were generally low (<25% and <0.15, respectively), suggesting better consistency. The highest heterogeneity was observed in comparisons involving antiplatelet-based strategies, likely reflecting differences in DAPT and SAPT definitions, durations, and local practice patterns.

The influence of direct evidence proportion for each network estimate is reported in Supplementary material online, *Figures S10*–S13. Direct vs. indirect evidence for each network is reported in Supplementary material online, *Figures S14*–S17.

### Sensitivity analyses

Sensitivity analyses were performed, restricting the follow-up period to 3 and 6 months (see Supplementary material online, *Tables S8*–S11). Results for DRT and major bleeding were similar to the results obtained in the primary analysis. In the ranking probabilities (*P*-score value), low-dose DOAC remained ranked with the higher probability of being the best therapy for both outcomes across both follow-up periods (see Supplementary material online, *Figures S18*–S21). Additionally, sensitivity analyses limited to observational studies revealed no substantial deviations from the main results (see Supplementary material online, *Tables S12*–S15 and *Figures S22*–S25).

### Quality assessment

The study found no evidence of publication bias for DRT (Egger's test *P* = 0.084), thromboembolic events (Egger's test *P* = 0.256), and all-cause mortality (Egger's test *P* = 0.668). However, there was an indication of publication bias for major bleeding endpoints (Egger's test *P* = 0.003), as shown in Supplementary material online, *Figures S26*–S29. The analysis revealed that most studies were deemed to have a significant risk of bias due to their observational design, as evaluated using the ROBINS-I tool (see Supplementary material online, *Figure S30*). The three RCTs included in the analysis were evaluated using the RoB-2 tool and presented some concerns for bias (see Supplementary material online, *Figure S31*).

Using CINeMA, most treatment comparisons were rated as providing low to very low certainty of evidence, driven by serious within-study bias (reflecting the predominance of observational designs), indirectness, and imprecision. Comparisons involving low-dose DOAC were additionally downgraded for imprecision due to small sample size and limited events. Only contrasts supported by randomized evidence reached moderate certainty, though these were few and underpowered. Full CINeMA ratings by domain are presented in Supplementary material online, *Tables S16*–S19.

## Discussion

### Evolution of antithrombotic strategies in left atrial appendage occlusion

LAAO represents a viable option for patients with non-valvular AF who face a high risk of bleeding or have contraindications to long-term anticoagulation therapy.^3^ Recent promising evidence from randomized data has upgraded the position of LAAO over traditional OAC therapy for thromboprophylaxis in AF patients,^11^ which may increase LAAO procedures worldwide. However, the optimal antithrombotic strategy following successful LAAO remains under constant debate and is still inadequately established due to insufficient supporting evidence.^1,5,12^ The field has undergone considerable evolution since the initial trials, with practice patterns adapting in response to emerging evidence and clinical experience. The PROTECT-AF trial initially recommended a treatment regimen consisting of warfarin and aspirin for 45 days, followed by DAPT through 6 months post-implant, and subsequently lifelong aspirin therapy.^13^ This approach aimed to prevent DRT while facilitating complete endothelialization of the device. As the field advanced, especially in Europe, there has been a notable shift towards approaches that emphasized antiplatelet therapy.^14^ The emphasis on antiplatelet therapy may stem from the early development of Amplatzer LAAO devices in Europe and the increased bleeding risk associated with European LAAO candidates. In the USA, the post-Watchman regimen has transitioned from a comprehensive protocol that included warfarin and aspirin to the current practice of full-dose DOAC combined with or without aspirin in most patients for the initial 6–12 weeks, subsequently followed by antiplatelet therapy for a minimum of 12 months.^9,15-17^ DAPT, despite being the most commonly employed regimen, does not consistently reduce the risk of major bleeding, thereby complicating decision-making in high-risk patients.^1,18^ Recently, DAPT received approval in the USA for use as post-Watchman antithrombotic treatment.^15,19^ The Amulet device has received FDA approval with a DAPT discharge regimen,^12^ and multiple studies regarding post-procedural DOAC treatment are currently underway in both the USA and Europe.^15^

### Direct oral anticoagulant vs. antiplatelet therapy (dual antiplatelet therapy/single antiplatelet therapy) following left atrial appendage occlusion

DOACs appear to be a good alternative for short-term anticoagulation therapy after LAAO, showing superior effectiveness than DAPT in preventing thromboembolic events while minimizing bleeding risks.^16,17,19^ However, the evidence on DOAC efficacy and safety post-LAAO remains limited.^15^

Our findings indicate that both standard- and low-dose DOACs have a potential advantage over DAPT and SAPT in preventing DRT, thromboembolism and major bleeding following LAAO. The superiority of DOACs over DAPT is a significant finding, as DAPT has been a common post-LAAO antithrombotic strategy. This aligns with recent trends in post-LAAO management.^15,20^ Our findings are consistent with previous studies that have questioned the efficacy of antiplatelet therapies in the LAAO setting.^16,17^ In the EWOLUTION registry, patients receiving DAPT post-LAAO had higher rates of thromboembolic complications and major bleeding compared with those on anticoagulants.^14^ Similarly, the PROTECT-AF trial demonstrated that DAPT was associated with a higher incidence of ischemic stroke and systemic embolism compared with anticoagulation.^3^ In a recent meta-analysis, monotherapy with DOAC had the highest likelihood of lower thromboembolic events and major bleeding compared with no antithrombotic therapy.^21^ While this study did not specifically compare low-dose DOACs to DAPT, it supports the trend toward DOAC use post-LAAO.^22^

Lowering the risk of major bleeding using DOACs, compared with DAPT or SAPT, is a particularly relevant finding in the context of LAAO, where patients are often elderly and have multiple comorbidities that increase their risk of bleeding.^12^ The OAC-ALONE trial, which compared OAC with antiplatelet therapy post-LAAO, found that anticoagulation was associated with a lower rate of haemorrhagic events than antiplatelet therapy.^23^ Our results further support the use of DOACs over antiplatelet therapies in this high-risk population.

### Low-dose direct oral anticoagulants: promising but off-label strategy

The introduction and use of low-dose DOACs as the next step in the evolution of antithrombotic therapy post-LAAO has shown promise as a novel strategy to prevent DRT.^20^ This NMA suggested that low-dose DOACs ranked favourably compared with other antithrombotic strategies and regimens across outcomes, appearing to reduce major bleeding compared with standard-dose regimens while maintaining similar protection against thromboembolism and device-related thrombosis. This finding, however, is based on a limited evidence base (1454 patients and 83 events across 13 cohorts, mostly observational). The strategy remains off-label, and current guidelines continue to recommend standard-dose anticoagulation or DAPT after LAAO.

Even though data on low-dose DOACs are limited and further research is needed to confirm their suggested benefit, these results challenge the traditional dose-response approach in anticoagulation therapy, where higher anticoagulant doses have conventionally been assumed to provide enhanced efficacy in preventing thromboembolic events.^24^ A biological rationale on the favourable effect of low-dose DOACs exists, as reduced intensity may lower bleeding risk without fully compromising antithrombotic efficacy, but this hypothesis requires formal testing. Ongoing randomized trials (ANDES—NCT03568890, SimpLAAfy—NCT06521463) will be decisive in confirming or refuting the role of low-dose DOACs. Until then, these results should be considered hypothesis-generating rather than practice-changing.

### Standard- vs. low-dose direct oral anticoagulants

This meta-analysis suggests that low-dose DOAC appear to significantly minimize bleeding risks, without increasing thromboembolic events or DRT, compared with standard-dose DOAC. These findings align with emerging data that endorse the ‘low-dose hypothesis’, indicating that lower doses of anticoagulation may be sufficient in structural interventions such as LAAO.^15,20,25^ Standard-dose DOACs have typically shown superior efficacy in preventing thromboembolism in the general AF population.^26^ For instance, a large meta-analysis by Ruff *et al*. found that standard-dose DOACs were associated with a significant reduction in stroke or systemic embolism compared with warfarin.^26^

### Efficacy profile of low-dose direct oral anticoagulant

A crucial finding of our analysis is a consistent and significant reduction in DRT and thromboembolism with low-dose DOAC compared with other antithrombotic therapies. DRT remains a major concern following LAAO, as it is associated with an increased risk of stroke and systemic embolism.^27^ Our findings align with those of the AMULET IDE trial, which indicated that anticoagulation therapy significantly reduced the incidence of DRT compared with DAPT.^28^

An interesting finding of this meta-analysis is the lower incidence of DRT (0.6%), compared with traditional cohorts and analyses.^27,29^ We believe that this could be attributed to several reasons. First, given the intention of this meta-analysis to maximize the association between DRT and the antithrombotic therapy at discharge, we selected the follow-up period that was closest to discharge, which resulted in fewer documented DRT events. Late occurrence of DRT is not infrequent.^30^ Their inclusion in our study would increase the incidence, but these late DRTs would most likely have a less direct association with post-LAAO antithrombotic treatment.^30,31^ Second, our analysis was primarily driven by the large SURPASS (WATCHMAN FLX SURveillance Post Approval AnalySiS Plan) subregistry, which included patients that received the second-generation LAA closure device (Watchman FLX, Boston Scientific) from 2020 until 2022.^17^ This registry had a very low DRT rate (0.3%). Finally, there is not agreement regarding definitions of DRT, and different imaging modalities are very different in their ability to detect it, with CT detecting much more DRTs than TEE.^32^ Finally, DRT incidence was likely underestimated, reflecting the use of older echocardiographic definitions that detect only large atrial-surface thrombi. Also, many studies did not mandate systematic imaging at 2–4 months, and definitions were heterogeneous, often limited to echocardiographically visible thrombi.

The exact mechanism by which low-dose DOACs mitigate DRT remains unclear, but it could be partially explained by the pathophysiology of thrombus development in the left atrium.^1,22^ The left atrium is susceptible to blood stasis, as it functions as a low-pressure chamber. Furthermore, the implantation of a metallic device in the left atrial appendage may activate the coagulation system.^33^ This environment may facilitate thrombus development via the coagulation cascade rather than via platelet aggregation.^34^ Antiplatelet agents focus on inhibiting platelet aggregation; however, the latter does not seem to be the primary mechanism of thrombus formation in the context of LAAO devices.^35^ In contrast, DOACs inhibit factor Xa or thrombin, thereby directly influencing the coagulation cascade, which plays a more significant role in DRT formation.^36^ Evidence from older but landmark trials, such as the PROTECT-AF and PREVAIL, further supports this perspective, indicating that anticoagulants significantly reduce DRT compared with antiplatelet regimens.^13,37^

Furthermore, occlusion of the left atrial appendage itself minimizes the risk of thrombus formation by eliminating the primary source of thrombus in patients with AF.^1^ Thus, high-dose anticoagulation may not offer additional benefit in this population but may instead increase the risk of adverse bleeding events, particularly in elderly patients or those with comorbidities that predispose them to bleeding.

The context of LAAO itself may alter the direct dose–response relationship.^38^ Hence, the efficacy of low-dose DOACs could be attributed to several factors. First, the lower doses could improve patient adherence, as reduced side effects may result in better compliance with the prescribed regimen, ultimately leading to better overall outcomes.^12^ The interruption of full-dose OAC due to bleeding could increase the subsequent risk of thromboembolism.^12^ It has been shown in prior studies, nevertheless, that suboptimal anticoagulation increases the risk of thrombus formation.^39^ Second, the LAAO device itself provides some degree of protection against thromboembolism, which might allow for effective anticoagulation at lower doses.^15^ Also, it has been shown that inflammation plays a role in DRT formation, and high doses of anticoagulants may exacerbate this process by increasing endothelial injury and platelet activation.^40^ Finally, the addition of non-thrombogenic coating to Watchman with the FLX Pro device may render the need for full-dose OAC.^41^ Of note, the SIMPLAAFY trial, which just started enrolling will compare low-dose DOAC, SAPT, and DAPT in patients getting Watchman FLX Pro.

### Safety profile of low-dose direct oral anticoagulant

Our analysis suggested that low-dose DOACs are associated with a significantly lower risk of major bleeding compared with all other antithrombotic strategies. This finding aligns with prior research that highlighted the safety benefits associated with the use of low-dose DOACs. Of note, in the ENGAGE AF-TIMI 48 trial, low-dose edoxaban led to a lower risk of major bleeding compared with VKA.^42^ The reduced risk of major bleeding with low-dose DOACs is particularly relevant in the context of LAAO, where patients often have multiple comorbidities and an inherently higher bleeding risk.^43^

The pharmacokinetics of DOACs significantly influence bleeding outcomes. Recent studies have shown that DOACs exhibit concentration-dependent inhibition of thrombin and factor Xa; however, this inhibition plateaus at elevated doses.^44^ This suggests that low-dose DOACs may effectively prevent thrombus formation while reducing the risk of bleeding, especially in the context of LAAO.^38^

### Comparisons with other meta-analyses

Prior meta-analyses and network meta-analyses have also investigated post-procedural antithrombotic strategies following LAAO, with varying scope and focus. In the 2023 network meta-analysis by Carvalho *et al*., seven antithrombotic regimens were compared, including DOAC monotherapy, DOAC plus SAPT, VKA, VKA plus SAPT, DAPT, SAPT alone, and no therapy.^20^ Their findings suggested that DOAC monotherapy was associated with the lowest risk of thromboembolic and bleeding events. However, that analysis did not stratify DOACs by dose, and the incidence of DRT was not significantly different across strategies.

A smaller NMA by Wang *et al*. included only 10 observational studies with fewer than 2000 patients and found that VKA ranked highest and DAPT lowest in terms of efficacy and safety but again did not distinguish between DOAC doses.^45^ More recently, two focused meta-analyses, by Lima *et al*.^46^ and Tabowei *et al*.^47^ specifically compared low-dose DOAC with DAPT, incorporating only 828 to 1015 patients. Both reported that low-dose DOAC was associated with significantly lower rates of DRT, thromboembolism, and major bleeding compared with DAPT. However, these analyses were limited by small sample sizes, fewer included studies, and the lack of comprehensive strategy-level comparisons.

In contrast, our present NMA is the most comprehensive and extensive NMA to date, incorporating 52 studies and nearly 70 000 patients, including over 1400 patients treated with low-dose DOACs. Unlike earlier NMAs, which either grouped DOACs together or focused on narrow comparisons, our findings demonstrate that both standard- and low-dose DOACs are significantly superior to antiplatelet-based and VKA-based strategies for reducing thromboembolic events, major bleeding, and DRT. Importantly, we show that low-dose DOACs are associated with a lower risk of major bleeding compared with standard-dose DOACs, without sacrificing thromboembolic protection. This distinction, along with the broader scope, updated evidence base (including post-2022 studies), and substantially greater population size, supports a nuanced, dose-conscious approach to post-LAAO antithrombotic therapy.

### Challenges and future directions

Despite the promising results of recent studies, several challenges remain in optimizing post-LAAO antithrombotic therapy. These include determining the optimal duration of therapy, refining patient selection criteria, gathering more data on long-term outcomes, considering device-specific factors, and evaluating the cost-effectiveness of various strategies.^43^

Several ongoing randomized trials, including the ANDES trial (NCT03568890), FADE-DRT (NCT04502017), and other large studies, such as CLOSURE-AF, CHAMPION-AF, and CATALYST, are expected to provide further clarity on the optimal antithrombotic regimen post-LAAO.^15^ These studies are anticipated to offer robust evidence on the comparative efficacy and safety of low-dose DOACs vs. other therapies, potentially reshaping the standard of care for post-LAAO patients.

### Strengths and limitations

Several aspects of this network meta-analysis must be considered. First, the included studies consist of a mixture of observational studies and a small number of RCTs, which may introduce heterogeneity in study design, patient populations, and antithrombotic protocols. Observational studies inherently carry a higher risk of bias, particularly in terms of selection bias and confounding, as we observed in our risk of bias assessments using the ROBINS-I tool. Given the predominance of observational evidence, a frequentist random-effects NMA was performed using study-level event data. Although advanced frameworks (e.g. design-adjusted or hierarchical models) are recommended in mixed-design networks, these were not applicable in our case due to the limited number and coverage of RCTs. Meta-regression using study design as a covariate was also not feasible, as RCTs were lacking for most treatment contrasts. Another limitation is that our analyses relied on crude, unadjusted event counts because regimen-stratified adjusted estimates were rarely available across included studies. The lack of detailed patient-level data prevented us from exploring potential confounding variables, such as renal function, comorbidities, and adherence to therapy, that may impact the outcomes. These limitations increase the likelihood of bias in the indirect comparisons.

Heterogeneity across studies was moderate to substantial, particularly for comparisons involving SAPT and DAPT, where definitions and duration of therapy varied widely. Also, bleeding and thromboembolic risk were unevenly distributed across regimens. As a result, indirect comparisons must be interpreted with caution. Although we attempted to control for this by conducting a sensitivity analysis and exploring publication bias, the limitations of the non-randomized data remain. While we performed sensitivity analyses restricting follow-up to 3 and 6 months to evaluate the robustness of our findings over time, we did not conduct additional analyses based on study design or risk of bias. This decision was primarily due to the limited number of randomized trials (*n* = 3) and the overall dominance of observational studies in the LAAO literature, which precluded meaningful stratification or meta-regression by study type. However, to evaluate the robustness of our findings, we conducted a sensitivity analysis restricted to observational studies, which yielded consistent effect estimates and treatment rankings. The follow-up periods across studies were not uniform, with some endpoints being evaluated at different time intervals. This inconsistency may affect the comparability of outcomes, particularly for long-term endpoints such as all-cause mortality and thromboembolic events. Shorter follow-up periods may have limited the capture of long-term complications, such as late DRTs, which may develop beyond six months post-implantation. Also, follow-up was based primarily on echocardiography, which underestimates DRT compared not only with systematic CT-based imaging at 2–4 months but also with older echocardiographic criteria that detect only large atrial-surface thrombi.

Although low-dose DOACs demonstrated promising results, the limited number of RCTs and the small sample size in low-dose DOACs highlight the need for further investigation and do not allow for robust treatment recommendations. Due to limited reporting of the specific DOAC agents and heterogeneity in how ‘low-dose’ regimens were defined across studies, we could not reliably assess molecule-specific effects (e.g. apixaban vs. rivaroxaban). As a result, potential differences between individual DOACs could not be captured, and our findings should be interpreted as class effects rather than agent-specific effects.

Treatment crossover, regimen discontinuation, and medication adherence could not be systematically captured, particularly in observational cohorts, and may have diluted treatment differences. Furthermore, variability in definitions of bleeding (e.g. TIMI, BARC, ISTH), and thromboembolic events across studies may have contributed to inconsistencies in the reporting of these outcomes. All-cause mortality was inconsistently reported (23 studies) and generally limited to short follow-up, precluding firm conclusions on long-term survival. The lack of uniform adjudication introduces uncertainty that may weaken the robustness of bleeding comparisons. Also, this precluded stratification by baseline bleeding or thromboembolic risk. In clinical practice, regimen selection is individualized: reduced-intensity strategies may be most appropriate for patients at very high bleeding risk, while standard-dose anticoagulation may be preferred in patients at higher thromboembolic risk. Such clinical nuances could not be captured in our synthesis. Our findings were rated as low or very low certainty in CINeMA, consistent with ROBINS-I and RoB2 assessments due to the predominance of observational evidence, residual confounding, non-uniform outcome definitions, and underreporting of events all contributed to downgrading. As such, our conclusions should be regarded as exploratory and hypothesis-generating, pending confirmation in large randomized trials.

## Conclusion

The field of post-LAAO antithrombotic management is undergoing significant advancements, with DOACs emerging as a leading therapeutic option. Our meta-analysis highlights their ability to provide a favourable balance between efficacy and safety, effectively reducing the risks of device-related thrombosis, thromboembolism, and bleeding compared with other strategies such as DAPT.

Emerging evidence suggests that low-dose DOACs may offer additional benefits by further minimizing bleeding risks without compromising efficacy. However, these findings should be interpreted cautiously due to the limited data available. Robust RCTs are essential to validate these results and establish comprehensive treatment recommendations.

Future research should prioritize optimizing patient selection, tailoring dosing strategies, defining ideal treatment durations, and assessing long-term outcomes to enhance the management of this high-risk population. Advances in LAAO devices with antithrombogenic coatings (e.g. Watchman FLX PRO) and strategies employing aspirin-only or even no pharmacological therapy are under investigation. These efforts may fundamentally alter the landscape of post-LAAO antithrombotic management and improve clinical outcomes following LAAO.

## Supplementary Material

pvaf078_Supplementary_Data

## Data Availability

The data underlying this article will be shared on reasonable request to the corresponding author
