# Supplementary material for: Antithrombotic strategies and DOAC dosing following left atrial appendage occlusion: a network meta-analysis
Source: Eur Heart J Cardiovasc Pharmacother. 2025 Nov 25;12(1):38–51. doi: 10.1093/ehjcvp/pvaf078 (PMC12862979; doi:10.1093/ehjcvp/pvaf078)
Supplement: pvaf078_Supplementary_Data [file pvaf078_supplementary_data.docx]

**Supplementary material**

**Low-Dose Direct Oral Anticoagulation After Left Atrial Appendage Occlusion: a Network Meta-Analysis**

Contents

[**Supplementary Table 1**. PRISMA Main Checklist 4](#_Toc209468328)

[**Supplementary Table 2.** PRISMA Abstract Checklist 9](#_Toc209468329)

[**Supplementary Table 3.** Details of the Search Strategy According to the Database 10](#_Toc209468330)

[**Supplementary Table 4.** Definitions of thromboembolic events, and major bleeding 12](#_Toc209468331)

[**Supplementary Figure 1.** Preferred Reporting Items for Systematic Reviews and Meta-Analyses (PRISMA) flow diagram. 17](#_Toc209468332)

[**Supplementary Table 5.** Characteristics of included studies 18](#_Toc209468333)

[**Supplementary Table 6.** Patterns and duration of antithrombotics in each study following LAAO 24](#_Toc209468334)

[**Supplementary Table 7.** Endpoint events, sample size, and follow-up duration, stratified by each post-LAAO antithrombotic therapy and by each study 34](#_Toc209468335)

[**Supplemental Figure 2.** Forest plots of random effects network meta-analysis for major bleeding across different reference groups: (A) vs low-dose DOAC, (B) vs DAPT, (C) vs DOAC plus SAPT, (D) vs SAPT, (E) vs standard-dose DOAC, (F) vs VKA, and (G) vs VKA plus SAPT. 42](#_Toc209468336)

[**Supplemental Figure 3.** Forest plots of random effects network meta-analysis for thromboembolism across different reference groups: (A) vs low-dose DOAC, (B) vs DAPT, (C) vs DOAC plus SAPT, (D) vs SAPT, (E) vs standard-dose DOAC, (F) vs VKA, and (G) vs VKA plus SAPT. 43](#_Toc209468337)

[**Supplementary Figure 4.** Forest plots of random effects network meta-analysis for device-related thrombosis across different reference groups: (A) vs low-dose DOAC, (B) vs DAPT, (C) vs DOAC plus SAPT, (D) vs SAPT, (E) vs standard-dose DOAC, (F) vs VKA, and (G) vs VKA plus SAPT. 44](#_Toc209468338)

[**Supplemental Figure 5.** Forest plots of random effects network meta-analysis for all-cause mortality across different reference groups: (A) vs low-dose DOAC, (B) vs DAPT, (C) vs DOAC plus SAPT, (D) vs SAPT, (E) vs standard-dose DOAC, (F) vs VKA, and (G) vs VKA plus SAPT. 45](#_Toc209468339)

[**Supplemental Figure 6**. Ranking probabilities (rankograms) for the outcome of major bleeding for each antithrombotic strategy. 46](#_Toc209468340)

[**Supplemental Figure 7**. Ranking probabilities (rankograms) for the outcome of thromboembolism for each antithrombotic strategy. 46](#_Toc209468341)

[**Supplemental Figure 8**. Ranking probabilities (rankograms) for the outcome of device-related thrombosis for each antithrombotic strategy. 47](#_Toc209468342)

[**Supplemental Figure 9**. Ranking probabilities (rankograms) for the outcome of all-cause mortality for each antithrombotic strategy. 47](#_Toc209468343)

[**Supplemental Figure 10**. Plot for direct evidence proportions in the network meta-analysis for major bleeding events 48](#_Toc209468344)

[**Supplemental Figure 11**. Plot for direct evidence proportions in the network meta-analysis for thromboembolic events 49](#_Toc209468345)

[**Supplemental Figure 12**. Plot for direct evidence proportions in the network meta-analysis for device-related thrombosis 50](#_Toc209468346)

[**Supplemental Figure 13**. Plot for direct evidence proportions in the network meta-analysis for all-cause mortality 51](#_Toc209468347)

[**Supplemental Figure 14**. Split network estimates illustrating the contribution of direct and indirect evidence and local inconsistency in network meta-analysis for major bleeding. 52](#_Toc209468348)

[**Supplemental Figure 15**. Split network estimates illustrating the contribution of direct and indirect evidence and local inconsistency in network meta-analysis for thromboembolic events. 53](#_Toc209468349)

[**Supplemental Figure 16**. Split network estimates illustrating the contribution of direct and indirect evidence and local inconsistency in network meta-analysis for device-related thrombosis. 54](#_Toc209468350)

[**Supplemental Figure 17**. Split network estimates illustrating the contribution of direct and indirect evidence and local inconsistency in network meta-analysis for all-cause mortality. 55](#_Toc209468351)

[**Supplemental Table 8**. League table of the sensitivity analysis for major bleeding restricting follow-up to 3 months. 56](#_Toc209468352)

[**Supplemental Figure 18**. Ranking probabilities (rankograms) of the sensitivity analysis for major bleeding restricting follow-up to 3 months. 56](#_Toc209468353)

[**Supplemental Table 9**. League table of the sensitivity analysis for major bleeding restricting follow-up to 6 months. 57](#_Toc209468354)

[**Supplemental Figure 19**. Ranking probabilities (rankograms) of the sensitivity analysis for major bleeding restricting follow-up to 6 months. 57](#_Toc209468355)

[**Supplemental Table 10**. League table of the sensitivity analysis for device-related thrombosis restricting follow-up to 3 months. 58](#_Toc209468356)

[**Supplemental Figure 20**. Ranking probabilities (rankograms) of the sensitivity analysis for device-related thrombosis restricting follow-up to 3 months. 58](#_Toc209468357)

[**Supplemental Table 11**. League table of the sensitivity analysis for device-related thrombosis restricting follow-up to 6 months. 59](#_Toc209468358)

[**Supplemental Figure 21**. Ranking probabilities (rankograms) of the sensitivity analysis for device-related thrombosis restricting follow-up to 6 months. 59](#_Toc209468359)

[**Supplemental Table 12**. League table presenting results of sensitivity analyses limited to observational studies, evaluating the relative odds of major bleeding across treatment comparisons. 60](#_Toc209468360)

[**Supplemental Figure 22**. Forest plot from random-effects network meta-analysis evaluating the risk of major bleeding, based on sensitivity analysis restricted to observational studies. 60](#_Toc209468361)

[**Supplemental Table 13**. League table presenting results of sensitivity analyses limited to observational studies, evaluating the relative odds of thromboembolism across treatment comparisons. 61](#_Toc209468362)

[**Supplemental Figure 23**. Forest plot from random-effects network meta-analysis evaluating the risk of thromboembolism, based on sensitivity analysis restricted to observational studies. 61](#_Toc209468363)

[**Supplemental Table 14**. League table presenting results of sensitivity analyses limited to observational studies, evaluating the relative odds of device-related thrombosis across treatment comparisons. 62](#_Toc209468364)

[**Supplemental Figure 24**. Forest plot from random-effects network meta-analysis evaluating the risk of device-related thrombosis, based on sensitivity analysis restricted to observational studies. 62](#_Toc209468365)

[**Supplemental Table 15**. League table presenting results of sensitivity analyses limited to observational studies, evaluating the relative odds of all-cause mortality across treatment comparisons. 63](#_Toc209468366)

[**Supplemental Figure 25**. Forest plot from random-effects network meta-analysis evaluating the risk of all-cause mortality, based on sensitivity analysis restricted to observational studies. 63](#_Toc209468367)

[**Supplemental Table 16**. Certainty of evidence ratings for major bleeding derived using the Confidence in Network Meta-Analysis (CINeMA) framework. 64](#_Toc209468368)

[**Supplemental Table 17**. Certainty of evidence ratings for thromboembolism derived using the Confidence in Network Meta-Analysis (CINeMA) framework. 65](#_Toc209468369)

[**Supplemental Table 18**. Certainty of evidence ratings for device-related thrombosis derived using the Confidence in Network Meta-Analysis (CINeMA) framework. 66](#_Toc209468370)

[**Supplemental Table 19**. Certainty of evidence ratings for all-cause mortality derived using the Confidence in Network Meta-Analysis (CINeMA) framework. 67](#_Toc209468371)

[**Supplemental Figure 26**. Funnel plot of network meta-analysis depicting the relationship between effect size versus standard error for the effect of different antithrombotic strategies on major bleeding events. 68](#_Toc209468372)

[**Supplemental Figure 27**. Funnel plot of network meta-analysis depicting the relationship between effect size versus standard error for the effect of different antithrombotic strategies on thromboembolic events. 69](#_Toc209468373)

[**Supplemental Figure 28**. Funnel plot of network meta-analysis depicting the relationship between effect size versus standard error for the effect of different antithrombotic strategies on device-related thrombosis. 70](#_Toc209468374)

[**Supplemental Figure 29**. Funnel plot of network meta-analysis depicting the relationship between effect size versus standard error for the effect of different antithrombotic strategies on all-cause mortality. 71](#_Toc209468375)

[**Supplemental Figure 30.** Risk of Bias in Non-randomized Studies of Interventions (ROBINS-I) 72](#_Toc209468376)

[**Supplemental Figure 31.** Risk of Bias in randomized studies (Revised Cochrane risk of bias tool for randomized trials RoB2) 73](#_Toc209468377)

###

### **Supplementary Table 1**. PRISMA Main Checklist

| **Topic** | **No** | **Item** | **Location where item is reported** |
| --- | --- | --- | --- |
| **TITLE** |  |  |  |
| **Title** | 1 | Identify the report as a systematic review. | Pag.1 |
| **ABSTRACT** |  |  |  |
| **Abstract** | 2 | See the PRISMA 2020 for Abstracts checklist | Pag.3 |
| **INTRODUCTION** |  |  |  |
| **Rationale** | 3 | Describe the rationale for the review in the context of existing knowledge. | Pag.4 |
| **Objectives** | 4 | Provide an explicit statement of the objective(s) or question(s) the review addresses. | Pag.4 |
| **METHODS** |  |  |  |
| **Eligibility criteria** | 5 | Specify the inclusion and exclusion criteria for the review and how studies were grouped for the syntheses. | Pag.5-6 |
| **Information sources** | 6 | Specify all databases, registers, websites, organisations, reference lists and other sources searched or consulted to identify studies. Specify the date when each source was last searched or consulted. | Pag.5 |
| **Search strategy** | 7 | Present the full search strategies for all databases, registers and websites, including any filters and limits used. | Supplementary |
| **Selection process** | 8 | Specify the methods used to decide whether a study met the inclusion criteria of the review, including how many reviewers screened each record and each report retrieved, whether they worked independently, and if applicable, details of automation tools used in the process. | Pag.5-6 |
| **Data collection process** | 9 | Specify the methods used to collect data from reports, including how many reviewers collected data from each report, whether they worked independently, any processes for obtaining or confirming data from study investigators, and if applicable, details of automation tools used in the process. | Pag.5 |
| **Data items** | 10a | List and define all outcomes for which data were sought. Specify whether all results that were compatible with each outcome domain in each study were sought (e.g. for all measures, time points, analyses), and if not, the methods used to decide which results to collect. | Pag.6 |
|  | 10b | List and define all other variables for which data were sought (e.g. participant and intervention characteristics, funding sources). Describe any assumptions made about any missing or unclear information. | Pag.6 |
| **Study risk of bias assessment** | 11 | Specify the methods used to assess risk of bias in the included studies, including details of the tool(s) used, how many reviewers assessed each study and whether they worked independently, and if applicable, details of automation tools used in the process. | Pag.6-7 |
| **Effect measures** | 12 | Specify for each outcome the effect measure(s) (e.g. risk ratio, mean difference) used in the synthesis or presentation of results. | Pag.6-7 |
| **Synthesis methods** | 13a | Describe the processes used to decide which studies were eligible for each synthesis (e.g. tabulating the study intervention characteristics and comparing against the planned groups for each synthesis (item 5)). | Pag.6-7 |
|  | 13b | Describe any methods required to prepare the data for presentation or synthesis, such as handling of missing summary statistics, or data conversions. | Pag.6-7 |
|  | 13c | Describe any methods used to tabulate or visually display results of individual studies and syntheses. | Pag.6-7 |
|  | 13d | Describe any methods used to synthesize results and provide a rationale for the choice(s). If meta-analysis was performed, describe the model(s), method(s) to identify the presence and extent of statistical heterogeneity, and software package(s) used. | Pag.6-7 |
|  | 13e | Describe any methods used to explore possible causes of heterogeneity among study results (e.g. subgroup analysis, meta-regression). |  |
|  | 13f | Describe any sensitivity analyses conducted to assess robustness of the synthesized results. | Pag.6-7 |
| **Reporting bias assessment** | 14 | Describe any methods used to assess risk of bias due to missing results in a synthesis (arising from reporting biases). | NA |
| **Certainty assessment** | 15 | Describe any methods used to assess certainty (or confidence) in the body of evidence for an outcome. | NA |
| **RESULTS** |  |  |  |
| **Study selection** | 16a | Describe the results of the search and selection process, from the number of records identified in the search to the number of studies included in the review, ideally using a flow diagram. | Pag.7-9 |
|  | 16b | Cite studies that might appear to meet the inclusion criteria, but which were excluded, and explain why they were excluded. | NA |
| **Study characteristics** | 17 | Cite each included study and present its characteristics. | Supplementary |
| **Risk of bias in studies** | 18 | Present assessments of risk of bias for each included study. | Supplementary |
| **Results of individual studies** | 19 | For all outcomes, present, for each study: (a) summary statistics for each group (where appropriate) and (b) an effect estimates and its precision (e.g. confidence/credible interval), ideally using structured tables or plots. | Pag.7-9 |
| **Results of syntheses** | 20a | For each synthesis, briefly summarise the characteristics and risk of bias among contributing studies. | Pag.7-9 |
|  | 20b | Present results of all statistical syntheses conducted. If meta-analysis was done, present for each the summary estimate and its precision (e.g. confidence/credible interval) and measures of statistical heterogeneity. If comparing groups, describe the direction of the effect. | Pag.7-9 |
|  | 20c | Present results of all investigations of possible causes of heterogeneity among study results. | Pag.7-9 |
|  | 20d | Present results of all sensitivity analyses conducted to assess the robustness of the synthesized results. | Supplementary |
| **Reporting biases** | 21 | Present assessments of risk of bias due to missing results (arising from reporting biases) for each synthesis assessed. | Supplementary |
| **Certainty of evidence** | 22 | Present assessments of certainty (or confidence) in the body of evidence for each outcome assessed. | NA |
| **DISCUSSION** |  |  |  |
| **Discussion** | 23a | Provide a general interpretation of the results in the context of other evidence. | Pag. 9-13 |
|  | 23b | Discuss any limitations of the evidence included in the review. | Pag. 13-14 |
|  | 23c | Discuss any limitations of the review processes used. | Pag.13-14 |
|  | 23d | Discuss implications of the results for practice, policy, and future research. | Pag.13 |
| **OTHER INFORMATION** |  |  |  |
| **Registration and protocol** | 24a | Provide registration information for the review, including register name and registration number, or state that the review was not registered. | Pag. 5 |
|  | 24b | Indicate where the review protocol can be accessed, or state that a protocol was not prepared. | Pag. 5 |
|  | 24c | Describe and explain any amendments to information provided at registration or in the protocol. | Pag.5 |
| **Support** | 25 | Describe sources of financial or non-financial support for the review, and the role of the funders or sponsors in the review. | Pag.18 |
| **Competing interests** | 26 | Declare any competing interests of review authors. | Pag.18 |
| **Availability of data, code and other materials** | 27 | Report which of the following are publicly available and where they can be found: template data collection forms; data extracted from included studies; data used for all analyses; analytic code; any other materials used in the review. | NA |

### **Supplementary Table 2.** PRISMA Abstract Checklist

| **Topic** | **No.** | **Item** | **Reported?** |
| --- | --- | --- | --- |
| **TITLE** |  |  |  |
| **Title** | 1 | Identify the report as a systematic review. | Yes |
| **BACKGROUND** |  |  |  |
| **Objectives** | 2 | Provide an explicit statement of the main objective(s) or question(s) the review addresses. | Yes |
| **METHODS** |  |  |  |
| **Eligibility criteria** | 3 | Specify the inclusion and exclusion criteria for the review. | Yes |
| **Information sources** | 4 | Specify the information sources (e.g. databases, registers) used to identify studies and the date when each was last searched. | Yes |
| **Risk of bias** | 5 | Specify the methods used to assess risk of bias in the included studies. | No |
| **Synthesis of results** | 6 | Specify the methods used to present and synthesize results. | Yes |
| **RESULTS** |  |  |  |
| **Included studies** | 7 | Give the total number of included studies and participants and summarise relevant characteristics of studies. | Yes |
| **Synthesis of results** | 8 | Present results for main outcomes, preferably indicating the number of included studies and participants for each. If meta-analysis was done, report the summary estimate and confidence/credible interval. If comparing groups, indicate the direction of the effect (i.e. which group is favoured). | Yes |
| **DISCUSSION** |  |  |  |
| **Limitations of evidence** | 9 | Provide a brief summary of the limitations of the evidence included in the review (e.g. study risk of bias, inconsistency and imprecision). | No |
| **Interpretation** | 10 | Provide a general interpretation of the results and important implications. | Yes |
| **OTHER** |  |  |  |
| **Funding** | 11 | Specify the primary source of funding for the review. | No |
| **Registration** | 12 | Provide the register name and registration number. | No |

### **Supplementary Table 3.** Details of the Search Strategy According to the Database

| **Database** | **Search strategy** |
| --- | --- |
| **PubMed/**  **MEDLINE** | ("Atrial Appendage"(mh) OR "Left Atrial Append*"(tiab) OR LAA (tiab)) AND (occlusion(tiab) OR occlud*(tiab) OR closure(tiab) OR exclusion(tiab) OR LAAC(tiab) OR LAAO(tiab) OR PLAATO(tiab) OR Watchman(tiab) OR "Cardiac Plug"(tiab) OR Amplatzer(tiab) OR ACP(tiab) OR Amulet(tiab) OR wavecrest(tiab) OR Occlutech(tiab) OR LAmbre(tiab) OR Ultraseal(tiab) OR "Sideris Patch"(tiab) OR Pfm(tiab) OR lariat(tiab) OR atriclip(tiab)) AND ((death (mh) OR died(tiab) OR mortality(tiab) OR "cardiovascular mortality"(tiab) OR "cardiovascular death*"(tiab)) OR (stroke(mh) OR infarction(tiab) OR cerebrovascular(tiab) OR ischemic*(tiab) OR thromb*(tiab) OR emboli*(tiab) OR 'systemic emboli*'(tiab) OR TIA(tiab) OR "transient ischemic attack"(tiab)) OR (device AND throb*) OR DRT OR 'device-related thromb*' OR (hemorrhage(mh) OR bleed*(tiab) OR hemorrhag*(tiab) OR major bleeding (tiab)) OR Antithrombotic* OR (Coumarins (mh) OR "Vitamin K antagonist*"(tiab) OR warfarin (tiab) OR VKA (tiab) OR Marevan(tiab)) OR ("Factor Xa inhibitors"(mh) OR "Xa Inhibitor" (tiab) OR "direct oral anticoagulant*"(tiab) OR "direct acting anticoagulant*"(tiab) OR "direct-acting anticoagulant*"(tiab) OR DOAC(tiab) OR "new oral anticoagulant*"(tiab) OR NOAC(tiab) OR apixaban(tiab) OR rivaroxaban(tiab) OR apixaban (tiab) OR Antithrombin (mh) OR dabigatran OR "Thrombin Inhibitor*" OR "Direct Thrombin") OR ("Platelet Aggregation Inhibitors"(mh) OR antiplatelet*(tiab) OR "Antiaggregant*"(tiab) OR aspirin(tiab) OR "acetylsalicylic acid" (tiab) OR ASA(tiab) OR "p2y12 inhibitor*"(tiab) OR clopidogrel(tiab) OR prasugrel(tiab) OR ticagrelor(tiab) OR "dual antiplatelet*"(tiab) OR DAPT(tiab) OR "single antiplatelet*"(tiab) OR SAPT(tiab))) |
| **EMBASE** | ('heart atrium appendage'/exp OR 'left atrial append*':ti,ab,kw OR laa:ti,ab,kw) AND ('left atrial appendage closure device'/exp OR occlusion:ti,ab,kw OR occlud*:ti,ab,kw OR closure:ti,ab,kw OR exclusion:ti,ab,kw OR laac:ti,ab,kw OR laao:ti,ab,kw OR plaato:ti,ab,kw OR watchman:ti,ab,kw OR 'cardiac plug':ti,ab,kw OR amplatzer:ti,ab,kw OR acp:ti,ab,kw OR amulet:ti,ab,kw OR wavecrest:ti,ab,kw OR occlutech:ti,ab,kw OR lambre:ti,ab,kw OR ultraseal:ti,ab,kw OR 'sideris patch':ti,ab,kw OR pfm:ti,ab,kw OR lariat:ti,ab,kw OR atriclip:ti,ab,kw) AND (('death'/exp OR 'mortality'/exp OR 'cardiovascular death'/exp OR died) OR ('cerebrovascular disease'/exp OR 'brain infarction'/exp OR 'brain ischemia'/exp OR 'stroke' OR 'brain hemorrhage'/exp OR cerebrovascular OR ischemic* OR thromb* OR emboli* OR 'systemic embolism' OR 'TIA' OR 'transient ischemic attack') OR (device AND throb*) OR DRT OR 'device-related thromb*' OR ('bleeding'/exp OR bleed* OR hemorrhag* OR 'major bleeding') OR Antithrombotic* OR ('coumarin anticoagulant'/exp OR 'antivitamin K'/exp OR 'Vitamin K antagonist*' OR warfarin OR VKA OR Marevan) OR ('anticoagulant agent'/exp OR 'Xa Inhibitor' OR 'direct oral anticoagulant*' OR 'direct acting anticoagulant*' OR 'direct-acting anticoagulant*' OR DOAC OR 'new oral anticoagulant*' OR NOAC OR apixaban OR rivaroxaban OR apixaban OR 'Antithrombin' OR dabigatran OR 'Thrombin Inhibitor*' OR 'Direct Thrombin') OR ('antithrombocytic agent'/exp AND 'Platelet Aggregation Inhibitors' OR 'antiplatelet*' OR 'Antiaggregant*' OR aspirin OR 'acetylsalicylic acid' OR ASA OR 'p2y12 inhibitor*' OR clopidogrel OR prasugrel OR ticagrelor OR 'dual antiplatelet therapy'/exp OR 'dual antiplatelet*' OR DAPT OR 'single antiplatelet*' OR SAPT)) |
| **COCHRANE** | ("Atrial Appendage" OR "Left Atrial Append*" OR LAAO AND (occlusion OR occlud* OR closure OR exclusion OR LAAC OR LAAO OR PLAATO OR Watchman OR "Cardiac Plug" OR Amplatzer OR ACP OR Amulet OR wavecrest OR Occlutech OR LAmbre OR Ultraseal OR "Sideris Patch" OR Pfm OR lariat OR atriclip) AND ((death OR died OR mortality OR "cardiovascular mortality" OR "cardiovascular death*") OR (stroke OR infarction OR cerebrovascular OR ischemic* OR thromb* OR emboli* OR 'systemic emboli*' OR TIA OR "transient ischemic attack") OR (device AND throb*) OR DRT OR 'device-related thromb*' OR (hemorrhage OR bleed* OR hemorrhag* OR major bleeding ) OR Antithrombotic* OR (Coumarins OR "Vitamin K antagonist*" OR warfarin OR VKA OR Marevan) OR ("Factor Xa inhibitors" OR "Xa Inhibitor" OR "direct oral anticoagulant*" OR "direct acting anticoagulant*" OR "direct-acting anticoagulant*" OR DOAC OR "new oral anticoagulant*" OR NOAC OR apixaban OR rivaroxaban OR apixaban OR Antithrombin OR dabigatran OR "Thrombin Inhibitor*" OR "Direct Thrombin") OR ("Platelet Aggregation Inhibitors" OR antiplatelet* OR "Antiaggregant*" OR aspirin OR "acetylsalicylic acid" OR ASA OR "p2y12 inhibitor*" OR clopidogrel OR prasugrel OR ticagrelor OR "dual antiplatelet*" OR DAPT OR "single antiplatelet*" OR SAPT)) |
| **SCOPUS** | ( atrial AND appendage OR laa AND oclussion OR laac OR laao OR plaato OR watchman OR "Cardiac Plug" OR amplatzer OR acp OR amulet OR wavecrest OR occlutech OR lambre OR ultraseal OR "Sideris Patch" OR pfm OR atriclip ) AND ( death OR mortality OR "cardiovascular mortality" OR stroke OR infarction OR cerebrovascular OR ischemic OR thromb OR emboli OR 'systemic AND emboli OR tia OR "transient ischemic attack" OR drt OR "device-related thromb" OR hemorrhage OR bleed OR major AND bleeding OR coumarins OR "Vitamin K antagonist" OR warfarin OR vka OR marevan OR "Factor Xa inhibitors" OR "Xa Inhibitor" OR "direct oral anticoagulant" OR "direct acting anticoagulant" OR "direct-acting anticoagulant" OR doac OR "new oral anticoagulant" OR noac OR apixaban OR rivaroxaban OR apixaban OR antithrombin OR dabigatran OR "Thrombin Inhibitor" OR "Direct Thrombin" OR "Platelet Aggregation Inhibitors" OR antiplatelet OR "Antiaggregant" OR aspirin OR "acetylsalicylic acid" OR asa OR "p2y12 inhibitor" OR clopidogrel OR prasugrel OR ticagrelor OR "dual antiplatelet" OR dapt OR "single antiplatelet" OR sapt ) |
| **ClinicalTrials.gov** | Left Atrial Appendage |

### **Supplementary Table 4.** Definitions of thromboembolic events, and major bleeding

| **Author - Year** | **Thromboembolism** | **Bleeding** |
| --- | --- | --- |
|  |  |  |
| **Freixa 2024**(1)  ADALA Randomized Clinical Trial | stroke, systemic embolism, and DRT | BARC type 3 and above |
| **Reinhardt 2024**(2)  NCDR LAAO – SURPASS | ischemic stroke, TIA and systemic embolism | bleeding requiring hospitalization, and/or causing a decrease in hemoglobin level >2 g/dL, and/or requiring blood transfusion that was not hemorrhagic stroke |
| **Mesnier 2024**(3) |  | life-threatening or disabling and major bleeding |
| **Moliner-Abós 2024**(4) |  | ISTH classification |
| **Li 2023**(5) | stroke or transient ischemic attack (TIA) determined on magnetic resonance imaging (MRI) or computed tomography (CT), peripheral thromboembolism, pulmonary embolism and venous thromboembolism | ISTH classification |
| **Zhang 2023**(6) | ischemic stroke/TIA | BARC type 3 and above |
| **Zhou 2023**(7) | strokes, transient ischemic attacks, and systemic embolism. Ischemic and hemorrhagic strokes were defined as the presence of clinically relevant focal neurological symptoms with consistent abnormalities on computed tomography or magnetic resonance imaging confirmed by a neurologist. | intracranial, retroperitoneal, intraspinal, intraocular, or pericardial hemorrhage as indicated by a decrease in hemoglobin concentrations of more than 2 g/dL or requiring transfusion of ≥2 units of packed red blood cells |
| **Bangash 2023**(8) | new stroke, TIA, or systemic embolism | cerebral, gastrointestinal, or other bleeding event requiring transfusion of at least 2 units of packed red blood cells |
| **Kailey 2023**(9) | ischemic stroke | BARC type 3 and above |
| **Zadori 2023**(10) | Definition missing | Definition missing |
| **Maarse 2023**(11) | Ischemic Stroke: Sudden onset of a focal or global neurological deficit with at least one of the following: Change in level of consciousness, Hemiplegia, Hemiparesis, One-sided numbness or sensory loss, Dysphasia or aphasia, Hemianopsia, Amaurosis fugax, Any other neurological signs or symptoms consistent with stroke AND Duration of neurological dysfunction >24 hours, Duration of neurological dysfunction <24 hours with imaging-documentend new infarction, Neurological dysfunction resulting in death. AND Absence: No other readily identifiable non-stroke cause for the clinical presentation to be determined by or in conjuction with a designated neurologist.  **TIA**: Focal neurological dysfunction not satisfying the above criteria for stroke, specifically lasting <24 hours and without imaging documented acute brain infarction. | BARC type 3 and above |
| **Margonato 2023**(12) | Definition missing | Type IIII or V of the Bleeding Academic Research Consortium classification |
| **Ge 2022**(13) | Stroke, transient ischemic attack (TIA), and systemic embolism (SE) | ISTH classification |
| **Fu 2022**(14) | ischemic stroke, TIA and systemic embolism | intracranial, retroperitoneal, intraspinal, intraocular or pericardial hemorrhage; a decrease in hemoglobin of >2 g/dL; or transfusion of ≥2 units of packed red blood cells |
| **Vignali 2022**(15) | Ischemic events were described in accordance with the Munich consensus document on definitions [ 35 ], and included any of the following complications: ischemic stroke, transient ischemic attack (TIA), DRT, evidence of systemic embolism (SE). | Hemorrhagic events were defined as major (≥3) bleeding event according to BARC classification: overt bleeding with hemoglobin decrease ≥3 g/dl; any transfusion with overt bleeding; cardiac tamponade; bleeding requiring surgical intervention; bleeding requiring intravenous vasoactive agents; intracranial hemorrhage; intraocular bleed compromising vision; coronary bypass-related bleeding within 48 h; fatal bleeding. |
| **Fukunaga 2022**(16) | Stroke/TIA based on MCC | BARC type 3 and above |
| **Li 2022**(17) | Stroke/TIA | BARC type 3 and above |
| **Pivato 2022**(18)  LIGATE | Stroke, TIA, and systemic embolism | BARC type 3 and above |
| **Su 2022**(19)  RECORD | Stroke, TIA, and systemic embolism accordingly to MMC | BARC type 3 and above |
| **Cepas-Guillen 2021**(20) | stroke, systemic embolism | BARC type 3 and above |
| **Della Rocca 2021**(21) | ischemic stroke, TIA, peripheral thromboembolism | ISTH classification  Major bleeding included those leading to a decrease in hemoglobin of ≥2.0 g/dL during a 24-hour period, transfusion of ≥2 U of packed red blood cells, bleeding at critical sites (eg, intracranial, intraspinal, retroperitoneal), or fatal ones. |
| **Schrag 2021**(22)  LAA-CAA cohort | Symptomatic ischemic stroke, transient ischemic attack, and intracerebral hemorrhage | Definition missing |
| **Briosa e Gala**(23) **2021** | -- | -- |
| **Casu 2021**(24) | Ischemic stroke | Major bleeding (definition missing) + intracranial hemorrhage |
| **Chen 2021**(25) | Ischemic stroke | Definition missing |
| **Faroux 2021**(26) | Ischemic stroke | BARC type 3 and above |
| **Mazzone 2021**(27) | Ischemic stroke and TIA | Overt bleeding associated with a drop in the hemoglobin level of at least 3.0 g/dl or requiring transfusion. |
| **Tjoe 2021**(28) | Cerebrovascular accident | Bleeding requiring at least 2 units packed red blood cell transfusion or surgery to correct. |
| **Zhu 2021**(29) | Ischemic stroke | Cerebral hemorrhage |
| **Duthoit 2020**(30)  ADRIFT Randomized Pilot Study | stroke (ischemic or hemorrhagic or transient ischemic attack), systemic embolism | Major or clinically significant Extracranial bleeding (ISTH) |
| **Asmarats 2020**(31) | -- | -- |
| **Barocelli 2020**(32) | Ischemic stroke, TIA, and others based on MCC | BARC type 3 and above |
| **Darmon 2020**(33) | Ischemic stroke and systemic embolization | BARC type 3 and above |
| **Patti 2020**(34) | Ischemic stroke and systemic embolic event | BARC type 3 and above |
| **Zhang 2020**(35) | - | - |
| **Şahiner 2019**(36) | Ischemic Stroke and TIA | Symptomatic bleeding in a critical area or organ (intracranial, intraspinal, retroperitoneal etc.) and bleeding causing a fall in hemoglobin level of 20 g/L or more or leading to transfusion of two or more units of whole blood or red cells. |
| **Cheung 2019**(37) | Stroke/TIA accordingly VARC-2 | VARC-2 |
| **Cohen 2019**(38) | Stroke (missing definition) | Fatal bleeding, bleeding into a critical organ (intracranial, intraspinal, intraocular, retroperitoneal, intraarticular, pericardial, intramuscular with compartment syndrome), or hemoglobin drop of ≥2 g/dL and/or transfusion of ≥2 units of whole blood or packed red cells within 48 h of index bleed. |
| **Fauchier 2018**(39)  RELEXAO | -- | -- |
| **Landmesser 2018**(40)  Amulet Observational | -- | BARC type 3 and above |
| **Peyrol 2018**(41) | Thromboembolic events | -- |
| **Zhang 2018**(42) | Stroke, TIA, and thromboembolic events | Definition missing |
| **Bergmann 2017**(43)  EWOLUTION | Ischemic stroke | Definition missing |
| **da Costa 2017**(44) | Thromboembolic event | Definition missing |
| **Korsholm 2017**(45) | Stroke and peripheral embolism | VARC-2 |
| **Saw 2017**(46) | Stroke/TIA accordingly VARC-2 | VARC-2 |
| **Enomoto 2016**(47) | Stroke, TIA, and systemic embolism | Intracranial, retroperitoneal, intraspinal, intraocular, or pericardial hemorrhage; drop in hemoglobin 42 g/dL; transfusion of ≥2 units of packed red blood cells. |
| **Tung 2016**(48) | Stroke was defined as a sudden onset focal neurological deficit lasting for at least 24 hours. Systemic embolism was defined as an acute vascular occlusion of an extremity or organ other than the brain. | Bleeding event leading to a hospital presentation requiring specialist medical or surgical treatment and/or a change in anticoagulant or antiplatelet therapy. |
| **Tzikas 2016**(49) | Stroke, TIA, and systemic embolism | Missing definition |
| **Bösche 2015**(50) | Stroke/TIA | Intracranial bleeding, hospitalization due to bleeding, hemoglobin decrease >2 mg/dL and/or transfusion of red blood cells. |
| **Wiebe 2015**(51) | Ischemic stroke or TIA | Missing definition and missing data stratified by antithrombotic therapy |
| **Meincke 2013**(52) | -- | Definition missing |


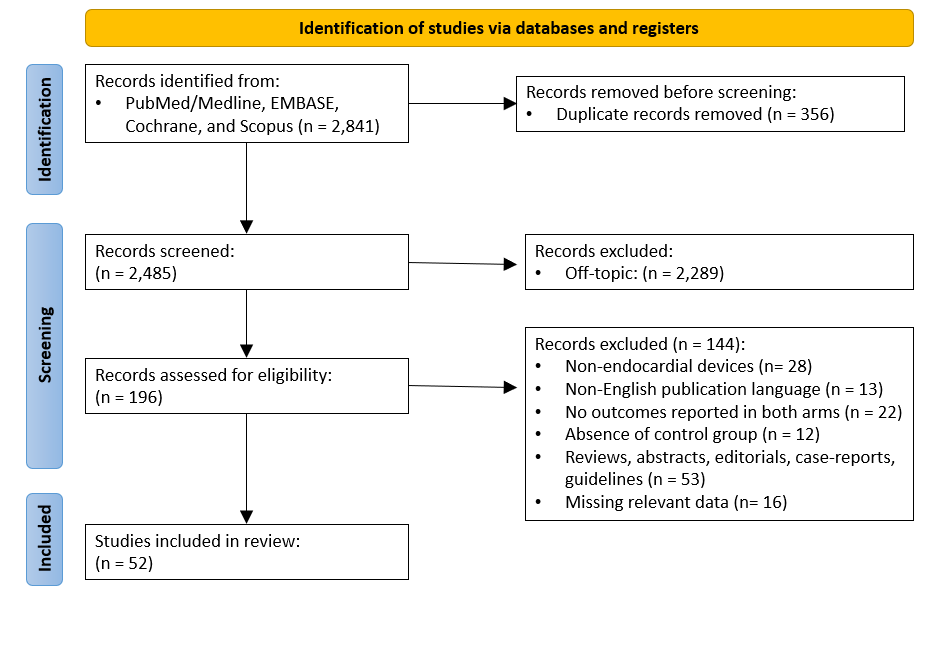


### **Supplementary Figure 1.** Preferred Reporting Items for Systematic Reviews and Meta-Analyses (PRISMA) flow diagram.

### **Supplementary Table 5.** Characteristics of included studies

| **Author - Year** | **Study design** | **Sample size (n)** | **Average follow-up** | **Age, mean** | **Male sex, n** | **Device type (%)** | **Paroxysmal AF** | **Persistent/Permanent AF, n** | **CHA2DS2-VASc Score, mean** | **HAS-BLED Score, mean** | **Prior major bleeding, n** | **Prior stroke, n** | **First DRT assessment** |
| --- | --- | --- | --- | --- | --- | --- | --- | --- | --- | --- | --- | --- | --- |
| **Freixa 2024**(1)  ADALA Randomized Clinical Trial | RCT | 90 | 3 months | 76.6 | 60 | Amulet(67.8), Lambre (8.9), Watchman FLX (23.3) |  | 56 | 4 | 3.5 | 53 | 17 | 1 month |
| **Reinhardt 2024**(2)  NCDR LAAO – SURPASS | Observational (retrosective) | 53878 | 6 months | 76.2 | 31788 | Watchman FLX (100) | 32073 | 21805 | 4.8 | 2.4 | 30749 | 7685 | 45 days |
| **Mesnier 2024**(3) | Observational (retrosective) | 1649 | 3 months | 76 | 967 | Amulet/ACP (72.5), Watchman (27.5) | 636 | 1013 | 4.3 | 3.6 | - | 712 | - |
| **Moliner-Abós 2024**(4) | Observational (retrospective) | 40 | 46.2 months | 76.6 | 72.5 | Amulet (92.5), Amplatzer (5), Watchman (2.5) | 90 | 10 | 4 | 3 | 7 | 12 | Not performed |
| **Li 2023**(5) | Observational (prospective) | 420 | 1 year | 68.8 | 267 | Watchman (100) |  |  | 3.7 | 3.1 | 50 | 186 | 45 days |
| **Zhang 2023**(6) | Observational (retrosective) | 203 |  |  | 97 | Watchman (58.6), Lacbes (41.4) | 100 | 103 |  |  |  |  | 2 months |
| **Zhou 2023**(7) | Observational (retrosective) | 457 | 2.7 years | 71.8 | 297 | ACP (23.9), Lambre (24.7), Watchman (51.4) | 174 | 287 | 4.5 | 3.8 |  | 295 | 45 days |
| **Bangash 2023**(8) | Observational (retrospective) | 103 | 12.2 months | 67.6 | 67 | Lambre (98), Watchman (2) | 42.6 | 57.4 | 4.7 | 1.9 |  | 100 |  |
| **Kailey 2023**(9) | Observational (retrospective) | 75 | 2.2 years | 72.9 | 72 | Amulet (100) | 35.6 | 49.2 | 3.8 | 3.5 | 32 | 31 | 3 months |
| **Zadori 2023**(10) | single-center retrospective | 136 | 2.7 ± 2.5 | 72.5 | 80 (58.8%) | Amplatzer Cardiac Plugs (33.8%), Amulet (36.8%), Watchman (23.3%), Watchman Flx (2.2%), LAmbre (3.7%) |  |  | 4.6 | 2.6 | 68 (50%) | 57 (41.8%) | no routine TOE |
| **Maarse 2023**(11) | single-center, nonrandomized, prospective cohort study | 230 | 5.2 ± 3.1 | 69.5 | 142 (61.7%) | Watchman 2.5 (93.9%), Amplatzer Amulet (6.1%) | 109 (47.4%) | 121 (52.6%) | 3.9 | 2.9 |  | 151 (65.6%) | 3 months |
| **Margonato 2023**(12) | single-center retrospective study | 102 | 3.9 (median) | 69 | 63 (62%) | Watchman 2.5 (21%), Watchman FLX (17%), Amplatzer Amulet (60%), Amplatzer Cardiac Plug (1%) | 22 (22%) | 80 (78%) | 3 | 2 |  |  | according to internal protocol |
| **Ge 2022**(13) | Observational (retrosective) | 84 |  | 68 | 54 | Watchman (100) | 21 | 63 | 3.87 | 2.68 |  | 42 | 6 weeks |
| **Fu 2022**(14) | Observational (retrosective) | 368 | 45 days |  | 236 | Watchman (100) | 62 | 306 | 4.5 | 3 | 75 | 234 | 45 days |
| **Vignali 2022**(15) | single-center mixed retrospective and prospective observational study | 123 | 2.7 (1.5, 3.5) median | 77 | 78 (60%) | Amplatzer (91%), Watchman (9%) |  |  | 4.46 | 3.23 |  | 66 (50.8%) | 3 months |
| **Fukunaga 2022**(16) | Observational (retrospective) | 55 | 360 days, mean | 74.1 | 36 | Watchman (100) | 24 | 31 | 4.6 | 3.8 | 34 | 28 | at 45 days |
| **Li 2022**(17) | Observational (retrospective) | 160 | 14.5 months, median | 69.13 | 118 | Watchman (100) | 77 | 83 | 3.66 | 4.16 | NA | 28 | at 3 months |
| **Pivato 2022**(18)  LIGATE | Observational (retrospective) | 52 | 117 (57–219) days, median | 75 | 36 | Ultraseal (100) | 14 | 38 | 4 | 3 | 42 | 11 |  |
| **Su 2022**(19)  RECORD | Observational (prospective) | 3032 | 30 days; | 69.1 | 1782 | Watchman (100) | 1249 | 1847 | 4 | 2.4 | 314 | 1418 |  |
| **Cepas-Guillen 2021**(20) | Observational (retrosective) | 139 | 3 months | 73.1 | 89 | Amulet/ACP (81), Lambre (17), Watchman (2) | 58 | 81 | 4.3 | 3.6 | 106 | 49 | 6 months |
| **Della Rocca 2021**(21) | Observational (prospective) | 555 | 14 months | 75.1 | 349 | Watchman (100) |  |  | 4 | 3 | 297 | 246 | 45 days |
| **Schrag 2021**(22)  LAA-CAA cohort | Observational (prospective) | 26 | 25 months, mean | 73 | 16 | Amulet (7.7), Watchman (65.4) |  |  | 4.6 | 3.8 | 13 | 6 |  |
| **Briosa e Gala**(23) **2021** | Observational (retrospective) | 229 | 3.9±3.7 years, mean | 74.4 | 165 | ACP (12.2), Amulet (3.5), Watchman (60.3), Watchman-FLX (24.0) | 61 | 168 | 4.4 | 3.2 | 50 | 49 | at 30 days |
| **Casu 2021**(24) | Observational (retrospective) | 120 | 16.1 ±10.2 months, mean | 74 | 84 | ACP (18.3), Amulet (35.9), Watchman (45.0), Other (0.8) | 51 | 69 | 4.16 | 3.56 | 71 | 34 |  |
| **Chen 2021**(25) | Observational (retrospective) | 340 | 868 days, mean | 64.9 | 208 | ACP (13.8), Watchman (86.2) | 5 | 335 | 3.1 | 1.8 |  | 145 | at 45 days |
| **Faroux 2021**(26) | Observational (retrospective) | 285 | 22 (8–38) months, median | 75.9 | 166 | ACP (3.5), Amulet (44.9), Watchman (39.3) | 86 | 199 | 4.6 | 3.6 | 160 | 86 | within 3 months |
| **Mazzone 2021**(27) | Observational (retrospective) | 260 | 420 days, median | 72.7 | 175 | ACP (21.2), Amulet (45.4), Watchman (31.5), Watchman-FLX (1.9) |  |  | 3.8 | 3.6 | 147 | 66 | at 3 months |
| **Tjoe 2021**(28) | Observational (retrospective) | 213 | 1 year | 77 | 135 | Watchman (100) | 109 | 94 | 4.1 | 3.2 | 72 | 56 | at 45 days |
| **Zhu 2021**(29) | Observational (retrospective) | 70 | 45 days | 66 | 41 | Watchman (100) |  |  | 3.6 | 3.2 | 9 | 53 | at 45 days |
| **Duthoit 2020**(30)  ADRIFT Randomized Pilot Study | RCT | 104 | 3 months | 77.2 | 65 | Watchman (34.6), Amulet (65.4) | 37 | 67 | 4.6 | 3.7 | 70 | 50 | 3 months |
| **Asmarats 2020**(31) | Observational (prospective) | 78 | 6 months | 75 | 53 | Watchman (100) | 37 | 41 | 5 | 4 |  | 29 | at 6 months |
| **Barocelli 2020**(32) | Observational (retrospective) | 60 | 2.32 years | 75.4 | 38 | ACP (58.6), Amulet (41.4) | 14 | 46 | 4.4 | 3.2 | 53 | 27 | at 1 month |
| **Darmon 2020**(33) | Observational (prospective) | 152 | 20.6±15.4, mean | 79.3 | 106 | ACP (9.2), Amulet (71.7), Watchman (19.1) |  |  | 4.4 | 3.8 |  | 60 | at 6 weeks |
| **Patti 2020**(34) | Observational (retrospective) | 610 | 12 months | 74.9 | 339 | ACP or Amulet (68.9), Watchman or other (31.1) | 226 | 384 | 4.09 | 3.33 | 340 | 202 | between 1 and 3 months |
| **Zhang 2020**(35) | Observational (retrospective) | 49 | 29.0±12.1 months | 65.6 | 22 | Watchman (100) | 4 | 45 | 3.5 | 2.6 |  | 9 | at 45-60 days |
| **Şahiner 2019**(36) | Observational (retrospective) | 60 | 21±15 months, mean | 72.3 | 25 | Amulet (100) | 13 | 47 | 4.61 | 4.32 | 53 | 13 | at 1, 6, and 12 months |
| **Cheung 2019**(37) | Observational (retrospective) | 161 | 28.3±24.4 months, mean | 71.4 | 109 | ACP/Amulet (47.5), Lambre (11.1), Watchman (41.4) | 47 | 114 | 4.1 | 2.9 | 63 | 62 | at 45±7 days |
| **Cohen 2019**(38) | Observational (retrospective) | 97 | 8 months | 76.9 | 61 | Watchman (100) | 35 | 61 | 4.7 | 3.5 | 24 | 39 |  |
| **Fauchier 2018**(39)  RELEXAO | Observational (retrospective) | 469 | 13±13 months, mean | 74.9 | 299 | Amplatzer (42), Watchman (58) |  | 244 | 4.5 | 3.7 | NA | 179 | at 1–3 months |
| **Landmesser 2018**(40)  Amulet Observational | Observational (prospective) | 1088 | 1 year | 75 | 702 | Amulet (100) |  |  | 4.2 | 3.3 | 780 | 300 | at discharge |
| **Peyrol 2018**(41) | Observational (retrospective) | 38 | 15±5 months, mean | 75.8 | 29 | Amulet (100) | 23 | 15 | 4.4 | 3.4 | 33 | 14 | between 6 and 12 weeks |
| **Zhang 2018**(42) | RCT | 99 | 18.7±7.4 months | 70 | 46 | Watchman (100) | 0 | 99 | 4.5 | 3.3 |  | 34 | at 45-60 days |
| **Bergmann 2017**(43)  EWOLUTION | Observational (prospective) | 1005 | 3 months | 73.4 | 601 | Watchman (100) |  |  | 4.5 | 2.3 | 318 | 194 | within 3 months |
| **da Costa 2017**(44) | Observational (retrospective) | 15 | 12 months | 69.4 | 9 | ACP (100) | 0 | 15 | 4.8 | 4.5 | 11 | 9 | at discharge |
| **Korsholm 2017**(45) | Observational (prospective) | 107 | 2.3 years, median | 73.2 | 77 | ACP (67.3), Amulet (32.7) |  | 51 | 4.4 | 4.1 | 88 | 54 | at 6 weeks |
| **Saw 2017**(46) | Observational (retrospective) | 339 | 355 (179–622) days, median | 74.4 | 228 | ACP (100) | 118 | 221 | 4.3 | 3 | 164 | 122 | at 134 days (median) |
| **Enomoto 2016**(47) | Observational (retrospective) | 426 | 4 months | 76 | 281 | Watchman (100) |  |  | 3.9 | 2.5 |  | NA | at 6 weeks to 4 months |
| **Tung 2016**(48) | Observational (Retrospective) | 47 | 2.4±1.7 years | 74.9 | 29 | Watchman (100) |  |  | 4.5 | 3 | 38 |  |  |
| **Tzikas 2016**(49) | Observational (retrospective) | 1047 | 13 months | 75 | 648 | ACP (100) | 594 | 453 | 4.5 | 3.1 | 492 | 404 | at 7 months |
| **Bösche 2015**(50) | Observational (retrospective) | 45 | 417 days | 75 | 26 | Watchman (100) |  |  | 4 | 3.5 |  | 14 | at 45 days |
| **Wiebe 2015**(51) | Observational (prospective) | 102 | 3.0±1.6 years | 71.6 | 64 | Watchman (100) |  |  | 4.3 | 2.9 |  | 18 | at 45 days and 3 months |
| **Meincke 2013**(52) | Observational (retrospective) | 59 | 6.3±0.46 months, mean | 73.6 | 35 | Watchman (100) | 15 | 44 | 4.4 | 3.5 | 36 | 11 | at 45 days |

### **Supplementary Table 6.** Patterns and duration of antithrombotics in each study following LAAO

| **Author - Year** | **standard-dose DOAC** | **low-dose DOAC** | **VKA** | **SAPT** | **DAPT** | **DOAC + SAPT** | **VKA + SAPT** | **Antithrombotic regimen (%)** | **Duration of ATT at discharge and de-escalation ATT** |
| --- | --- | --- | --- | --- | --- | --- | --- | --- | --- |
| **Freixa 2024**(1)  ADALA Randomized Clinical Trial |  | x |  |  | x |  |  | ldDOAC-api (50), DAPT (50) | Patients in the low-dose DOAC group received apixaban (2.5 mg twice daily) for 3 months post-LAAO, then switched to aspirin (100 mg/day). The DAPT group received aspirin (100 mg/day) and clopidogrel (75 mg/day) for 3 months, followed by aspirin alone (100 mg/day). |
| **Reinhardt 2024**(2)  NCDR LAAO – SURPASS | x |  | x | x | x | x | x | SAPT (2.6), DAPT (8.1), VKA (2.4), DOAC (22.5),  VKA+SAPT (8.4), DOAC+SAPT (53.2) | The most common discharge strategy was DOAC plus aspirin (48.3%), followed by DOAC alone (22.6%) and DAPT (8.1%). Over 75% of patients were discharged on a DOAC-based regimen. Warfarin was used in 11% of cases (warfarin plus aspirin 7.7%, warfarin alone 2.4%). Smaller groups received SAPT (2.6%), triple therapy (1.6%), or no antithrombotic therapy (0.7%). Anticoagulation use decreased by 45 days and was rare at 6 months, while DAPT and SAPT usage increased over time. |
| **Mesnier 2024**(3) | x |  | x | x | x |  |  | None (3.6), SAPT (25), DAPT (39.1), VKA (9), DOAC (19.8) | not defined |
| **Moliner-Abós 2024**(4) | x |  |  | x |  |  |  | LMWH (10), DOAC (7.5), LMWH+SAPT (2.5), SAPT (80) | At discharge, single antiplatelet therapy (SAPT, 80%) was the most common regimen, followed by anticoagulation (18%). During follow-up, 90% of patients discontinued anticoagulation. By the end, 60% remained on SAPT, 30% had no antithrombotic therapy, and a few continued anticoagulation for specific conditions such as ischemic stroke or severe device-related issues. |
| **Li 2023**(5) |  | x |  |  | x |  |  | ldDOAC-riv10/15 (66.6), DAPT (33.3) | The RRD group received rivaroxaban (10 mg or 15 mg) for 45 days post-procedure, then transitioned to DAPT (aspirin 100 mg + clopidogrel 75 mg) after confirming device stability and no significant peridevice leak on 45-day TEE. After 6 months, mono-antiplatelet therapy was continued indefinitely. Another group was on DAPT for 180 days post-implantation, followed by long-term aspirin therapy. If 45-day TEE revealed DRT, full-dose rivaroxaban (20 mg/day) was initiated until a subsequent TEE confirmed DRT resolution. |
| **Zhang 2023**(6) | x | x |  |  |  | x |  | ldDOAC-riv10 (40.9), fdDOAC (59.1) | Patients who underwent percutaneous coronary stenting received rivaroxaban plus clopidogrel for 60 days postoperatively. Others received rivaroxaban only (10 mg for elderly patients, 20 mg for non-elderly). After 60 days, DAPT (aspirin + clopidogrel) was administered until 6 months, followed by long-term SAPT with aspirin. |
| **Zhou 2023**(7) |  | x | x |  |  |  |  | ldDOAC-riv10/15 (74.8), VKA (25.2) | Oral anticoagulation was usually continued for at least 45 days after successful LAAC, whereas DAPT (aspirin 100 mg plus clopidogrel 75 mg) was continued for 1.5–6 months. Lifelong SAPT was subsequently administered. |
| **Bangash 2023**(8) | x | x |  |  | x |  |  | DAPT (6), standard-dose DOAC (31.1), low-dose DOAC (61.2) | At discharge, all patients were prescribed anticoagulants based on physician preference and patient condition. Rivaroxaban (75.24%) was the most common, followed by dabigatran (4.75%) and apixaban (5.94%). DAPT was prescribed in 5.94%. Rivaroxaban doses (20 mg or 15 mg) varied. After TEE/CT confirmed no DRT or PDL, NOACs were discontinued and replaced with DAPT for 1–3 months. NOAC use decreased from 52.4% at 3 months to 11.8% at 12 months. |
| **Kailey 2023**(9) | x |  | x | x | x |  |  | SAPT (34.7), DAPT (2.7), DOAC (29.3), VKA (12), None (21.3) | Standard treatment included anticoagulation (e.g., apixaban, rivaroxaban, edoxaban, dabigatran, or warfarin) or DAPT (aspirin + clopidogrel) for 6 weeks to 6 months. Minimal treatment consisted of either no therapy or SAPT. |
| **Zadori 2023**(10) | x |  | x | x | x |  |  | LMWH 1.5%, VKA 0.7 %, DOAC 3.0%, DAPT 45.2%, SAPT 45.2%, None 2.2% | Patients typically received DAPT for 3 months, followed by long-term SAPT. In cases of high bleeding risk, SAPT was used initially and maintained, or antithrombotic therapy was discontinued entirely in a few cases. |
| **Maarse 2023**(11) | x |  |  | x | x | x |  | DOAC+SAPT 7%, DOAC 49%, DAPT 20%, SAPT 21%, None 3% | After 3 months, de-escalation to SAPT was implemented when possible. At the first follow-up, most patients reduced their antithrombotic regimen, though 22% (46/210) remained on OAT. After one year, OAT use decreased to 12% (23/194). OAT was continued in cases of high thromboembolic risk, planned cardioversion or ablation, thromboembolic events, PDL, or suspected DRT. Between one year and the end of the study, OAT was reinitiated in 19% (36/194) of patients. |
| **Margonato 2023**(12) | x |  | x |  | x |  |  | LMWH 6%, DAPT 30%, VKA 29%, DOAC 35% | not defined |
| **Ge 2022**(13) |  | x | x |  |  |  |  | ldDOAC-dab (45.2), VKA (54.8) | Patients received low-dose dabigatran (110 mg twice daily) or warfarin (INR 2–3) for at least 45 days post-LAAO, followed by DAPT (aspirin 100 mg + clopidogrel 75 mg daily) for 6 months. Thereafter, aspirin monotherapy was continued for life. |
| **Fu 2022**(14) |  | x | x |  |  |  |  | ldDOAC-riv/dab (79.1), VKA (20.9) | Patients with no or minimal pericardial effusion were discharged on NOACs or warfarin for at least 45 days. Follow-up TEE or CT at 45 days assessed device stability, positioning, and residual flow. If no thrombus and complete sealing or minimal residual flow (<5 mm) were confirmed, NOACs or warfarin were discontinued, and DAPT (aspirin 100 mg + clopidogrel 75 mg daily) was prescribed for six months. |
| **Vignali 2022**(15) |  |  |  | x | x |  |  | SAPT 39.8%, DAPT 60.2% | Short-duration DAPT was used for 1 month, followed by de-escalation to SAPT. Long-duration DAPT lasted 12 months before de-escalation to SAPT. |
| **Fukunaga 2022**(16) |  |  |  |  | x | x | x | VKA+SAPT (49.1), DOAC+SAPT (40.0), DAPT (10.9) | If DRT or ischemic stroke/systemic embolism was detected on routine imaging, warfarin or an appropriate-dose DOAC was reinstated, with follow-up imaging conducted after approximately 3 months. |
| **Li 2022**(17) | x |  | x |  |  | x |  | DOAC/VKA (51.2), DOAC+SAPT (48.8) | OAC/NOAC therapy included warfarin (INR 2–3), rivaroxaban (15 mg daily), or dabigatran (110 mg twice daily). NOAC+SAPT regimens combined rivaroxaban or dabigatran with aspirin (100 mg daily) or clopidogrel (75 mg daily). TEE or CCTA at 3 months assessed for DRT or peri-device leaks (<5 mm). If stable, regimens transitioned to DAPT (aspirin + clopidogrel) for 3 months, followed by lifelong monotherapy with aspirin or clopidogrel. |
| **Pivato 2022**(18)  LIGATE | x |  |  | x | x |  |  | DAPT (84.6), DOAC (5.8), SAPT (5.8), None (1.9) | Most patients (84.6%) were discharged on DAPT, recommended for 1 month in 34.1% of cases and 3 months in 52.3%. A few patients were discharged on SAPT (3), OACs (3), or low-molecular-weight heparin (3). No patients were discharged on warfarin. One patient with intracranial bleeding was discharged without antithrombotic therapy. |
| **Su 2022**(19)  RECORD | x |  | x | x | x | x | x | DAPT (5.0), DOAC (66.7), DOAC+SAPT (12.7), SAPT (1.6), SAPT+VKA (1.1), VKA (12.0), Others (0.8) | Antithrombotic therapies were maintained for at least 30 days. |
| **Cepas-Guillen 2021**(20) |  | x |  | x | x |  |  | ldDOAC-api (29), DAPT (53), SAPT (18) | The first follow-up imaging, typically TEE, was performed between 6–12 weeks. For patients intolerant to TEE, cardiac CT was used. A satisfactory TEE result (complete LAAO without DRT) allowed withdrawal of one antiplatelet agent in DAPT or modification of low-dose apixaban therapy. SAPT with aspirin became the most common treatment after three months and was usually continued indefinitely. |
| **Della Rocca 2021**(21) |  | x |  |  |  | x |  | ldDOAC-api/riv (35.7), DOAC+SAPT (64.3) | The first group received full-dose DOAC and aspirin (81 mg) at discharge, de-escalating to DAPT after 45 days and SAPT after 6 months if no leak or DRT was detected on TEE. If a significant leak (>5 mm) was found at 45-day TEE, full-dose DOAC with aspirin was continued, transitioning to full-dose DOAC monotherapy if the leak persisted, with percutaneous closure scheduled 6–12 months post-LAAO. The second group received low-dose DOAC and aspirin (81 mg) for 45 days, continuing low-dose DOAC thereafter. For patients with a significant leak (>5 mm) on the first and second TEE, full-dose DOAC monotherapy was maintained until percutaneous closure 6–12 months post-LAAO. |
| **Schrag 2021**(22)  LAA-CAA cohort | x | x | x | x | x |  |  | DAPT (7.7), SAPT (46.2), VKA (23.1), DOAC (19.2), None (3.8) | The bridging thromboprophylaxis approach after LAAC was tailored to each patient's hemorrhage risk. High-risk patients, such as those with symptomatic ICH, were prescribed SAPT or DAPT for 6 weeks, while those who tolerated anticoagulation continued on OAC or DOAC for 6 weeks. After confirmatory imaging (TEE or CT), therapy was discontinued in the highest-risk patients. Low-dose aspirin was maintained in lower ICH risk patients or as mandated by IDE clinical trial protocols. |
| **Briosa e Gala**(23) **2021** | x |  | x |  | x |  |  | DAPT (81), OAC (0.9), SAPT (10), None (0.5) | At discharge, 81% of patients were started on DAPT. If postprocedural imaging was satisfactory, 90% transitioned to SAPT or no antiplatelet therapy. The median time to DAPT discontinuation was 62 days. After the first follow-up, 90% were on SAPT (46.3% aspirin, 39.3% clopidogrel) or no antiplatelet therapy. |
| **Casu 2021**(24) |  |  |  | x | x |  |  | DAPT (54.2), SAPT (30.0), None (4.2) | After the procedure, 30% of patients were discharged on SAPT, while 54.2% received DAPT. A short DAPT course (1–3 months) was prescribed in 42.5%, and 11.7% received a longer course (6–12 months). Other antithrombotic therapies were used in 11.6%, and 4.2% received no antithrombotic therapy. |
| **Chen 2021**(25) | x |  | x |  |  |  |  | DOAC (49.7), VKA (50.3) | Warfarin was prescribed for at least 45 days post-LAAC. If TEE confirmed successful closure (residual flow <5 mm) and no device-related thrombosis, warfarin was discontinued, and patients switched to DAPT (aspirin + clopidogrel) for 4.5 months, followed by long-term aspirin monotherapy. No data were available for DOAC use. |
| **Faroux 2021**(26) | x |  |  |  | x |  |  | DAPT (80.6), DOAC (19.4) | Post-LAAC antithrombotic management included short-term (1–3 months) DAPT (low-dose aspirin + clopidogrel 75 mg) or DOAC therapy, with the strategy determined by the physician's clinical judgment. |
| **Mazzone 2021**(27) | x |  |  | x | x | x |  | DAPT (71.5), DOAC (19.2), SAPT or none (4.6), APT+DOAC (4.6) | The standard strategy involved DAPT (ASA 81–300 mg daily + clopidogrel 75 mg daily) without a loading dose. After 3 months, patients transitioned to SAPT (ASA 81–300 mg daily) if TEE showed no major leaks or device-related thrombosis. For higher embolic risk without OAC contraindications, VKA or NOACs were used at discharge, followed by de-escalation to DAPT or SAPT after 3 months. Patients with prohibitive hemorrhagic risk (e.g., recent major bleeding) were discharged on SAPT or without antithrombotic therapy. |
| **Tjoe 2021**(28) | x |  |  |  |  | x | x | DOAC+SAPT (38.0), VKA+SAPT (28.6), DOAC (33.3) | Following an FDA-approved protocol, TEE was performed 45 days post-implant to check for device leaks (>5 mm shunting) or thrombosis. If no issues were detected, anticoagulation was stopped and replaced with DAPT for 6 months, followed by lifelong aspirin. If a leak or thrombus was found, anticoagulation continued until resolution was confirmed by follow-up TEE (at 90 days or later), after which DAPT for 6 months and then lifelong aspirin were prescribed. |
| **Zhu 2021**(29) | x |  | x |  |  |  |  | NOAC (57.1), VKA (42.9) | Following LAAC, all patients should receive oral anticoagulation with warfarin or DOACs as recommended and be followed up in outpatient clinic 45 days post-surgery. |
| **Duthoit 2020**(30)  ADRIFT Randomized Pilot Study |  | x |  |  | x |  |  | ldDOC-riv 10/15 (68.3), DAPT (31.7) | 3 months duration of ATT after discharge. NO de-escalation in the first 3 months. NO follow up after 3 months. |
| **Asmarats 2020**(31) | x |  | x | x | x |  |  | DAPT (39.8), DOAC (33.3), SAPT (21.8), VKA (5.1) | Patients were discharged on dual APT for 3 months followed by lifelong aspirin (single APT when deemed at too high bleeding risk) or under OAC for 45 days and then aspirin for life in the absence of absolute contraindications. The final decision was left at the physician's discretion. |
| **Barocelli 2020**(32) |  |  |  | x | x |  |  | DAPT (75.9), SAPT (24.1) | Dual antiplatelet therapy (DAPT) with acetylsalicylic acid (ASA) 100 mg daily and clopidogrel 75 mg daily for 1–6 months was prescribed after the device implantation. Patients deemed at very high bleeding risk were treated with single antiplatelet therapy. In the group of patients discharged with DAPT the median duration of therapy was 82 days (interquartile range of 30–105 days). |
| **Darmon 2020**(33) |  |  |  | x | x |  | x | DAPT (37.5), SAPT (47.3), SAPT+VKA (0.7), None (14.5) | Post-LAAC antithrombotic management was guided by the operator and heart team, including bleeding specialists. OACs were generally avoided, with strategies focusing on antiplatelet therapy. Discharge regimens included DAPT (low-dose aspirin 75–160 mg + clopidogrel 75 mg daily) for 6 weeks to 6 months followed by lifelong SAPT, lifelong SAPT alone, or no antithrombotic therapy (NATT). |
| **Patti 2020**(34) |  |  |  | x | x |  |  | DAPT (54.1), SAPT (45.9) | The majority of patients on SAPT received low-dose aspirin (95%), with the remainder on clopidogrel. SAPT was typically continued up to 1 year. DAPT (low-dose aspirin + clopidogrel 75 mg) lasted an average of 3.6±3.3 months, with aspirin monotherapy commonly continued after DAPT cessation. |
| **Zhang 2020**(35) | x |  | x |  |  |  |  | DOAC (53.1), VKA (46.9) | Low-molecular-weight heparin was given postoperatively. Warfarin or NOACs were administered for 45–60 days. If TEE confirmed complete LAA closure and no thrombus, patients transitioned to DAPT (aspirin + clopidogrel) until 6 months, followed by lifelong enteric-coated aspirin (100 mg daily). |
| **Şahiner 2019**(36) |  | x | x | x | x |  |  | DAPT (88.3), DOAC (8.3), SAPT (3.3) | Postprocedural antiplatelet therapy was tailored to each patient, involving either DAPT, SAPT, or low-dose anticoagulant therapy based on individual thromboembolic and bleeding risks. |
| **Cheung 2019**(37) |  |  |  |  | x |  | x | DAPT (58.6), SAPT+VKA (41.4) | All patients were prescribed lifelong aspirin (80 mg daily). For ACP/Amulet and LAmbre devices, clopidogrel (75 mg) was given for six months. For the WATCHMAN device, warfarin was used for at least 45 days until TEE confirmed peri-device flow <5 mm, after which clopidogrel was continued until six months post-implant. |
| **Cohen 2019**(38) | x |  | x |  |  |  |  | DOAC (53.6), VKA (46.4) | In the warfarin group, 95% of patients, and in the NOAC group, 98% of patients, successfully tolerated at least 6 weeks of OAC. Nearly all patients discontinued OAC within 90 days post-procedure. |
| **Fauchier 2018**(39)  RELEXAO | x |  | x | x | x | x |  | DAPT (23.2), OAC (28.8), OAC+SAPT (4.3), SAPT (36.2), None (7.5) | Subsequent antithrombotic therapy data were available for 77% of patients with device-related thrombus. Among them, 30% underwent a therapy change, primarily involving the initiation of OAC in patients not previously on it. |
| **Landmesser 2018**(40)  Amulet Observational |  |  |  | x | x | x | x | OAC±APT (17.6), SAPT (22.5), DAPT (57.6), None (2.2) | The protocol recommended patients take aspirin ≥ 6 months post-LAAO and clopidogrel per standard of care. The final decision was left at the physician's discretion. |
| **Peyrol 2018**(41) |  |  |  | x | x |  |  | DAPT (71.1), SAPT (26.3), None (2.6) | Following the manufacturer's guidelines, DAPT (aspirin 75 mg + clopidogrel 75 mg daily) was prescribed for at least 6 weeks unless contraindicated. If TEE showed no abnormalities (e.g., thrombus or peri-device leak >5 mm), clopidogrel was discontinued, and lifelong aspirin monotherapy (75 mg daily) was continued. |
| **Zhang 2018**(42) | x |  | x | x |  |  |  | DOAC (33.3), SAPT (33.3), VKA (33.3) | The treatment regimens included:  Warfarin Group: Warfarin (INR 2.0–3.0) for 45 days, then DAPT (aspirin 100 mg + clopidogrel 75 mg) for up to 6 months, followed by aspirin (100 mg/day).  Dabigatran Group: Dabigatran (110–150 mg twice daily, adjusted for age and renal function) for 45 days, then DAPT for 45 days to 6 months, followed by aspirin (100 mg/day).  Dual Antiplatelet Group: DAPT (aspirin 100 mg + clopidogrel 75 mg) for 6 months, followed by aspirin (100 mg/day). |
| **Bergmann 2017**(43)  EWOLUTION | x | x | x | x | x |  |  | DAPT (60.2), NOAC (10.8), VKA (15.5), SAPT (6.9), None (6.5) | Antithrombotic management of patients after successful LAAC was at the operator's discretion, with a duration of 3 months. |
| **da Costa 2017**(44) |  |  |  | x | x |  |  | SAPT (53.3), DAPT (20.0) None (26.7) | Immediately post-procedure, anticoagulants were discontinued, and DAPT was initiated with ASA (200 mg/day) and clopidogrel (300 mg loading dose, then 75 mg/day maintenance) for 6 months. |
| **Korsholm 2017**(45) |  |  |  | x | x |  |  | DAPT (12.1), SAPT (87.9) | Most patients (87.8%) were discharged on SAPT, while 12.2% received DAPT. At 6 months, 89.9% were still on SAPT. By the 12-month follow-up, 26% of LAAO patients were no longer on SAPT or any antithrombotic therapy. |
| **Saw 2017**(46) | x |  | x | x | x |  |  | DAPT (62.3), OAC (6.3), SAPT (40.0), None (0.4) | The device manufacturer recommended antithrombotic therapy with aspirin (80–100 mg/day) and clopidogrel (75 mg/day) for 1–3 months, followed by aspirin alone (80–100 mg/day) for at least another 3 months. However, therapy choice and duration were individualized based on physician preference and documented at admission and follow-up visits. |
| **Enomoto 2016**(47) | x |  | x |  |  |  |  | DOAC (50.2), VKA (49.8) | Patients received NOAC or warfarin for 6 weeks. Follow-up imaging (TEE or CT) was performed between 6 weeks (60%) and 4 months (40%) to evaluate thrombus apposition and LAA closure. CT confirmed no peri-device leak by the absence of contrast enhancement. For patients without imaging, anticoagulation continuation was determined individually by the physician. |
| **Tung 2016**(48) |  |  |  | x | x | x | x | OAC+APT (46.8), SAPT (12.8), DAPT (23.4) | Post-implant, 66% of patients were on SAPT, with 87% using aspirin (75–100 mg daily) and 13% using clopidogrel (75 mg daily). By three months, 89.4% remained on SAPT (95% aspirin, 5% clopidogrel). Anticoagulation was not used post-implant in 40.4% of patients, of whom 36.8% were on SAPT and the rest on DAPT. By day 45, 70.2% were no longer on anticoagulation. Only four patients received triple therapy (anticoagulation + DAPT) for a maximum of 60 days. |
| **Tzikas 2016**(49) | x |  | x | x | x | x | x | SAPT (34.7), SAPT+DOAC (1.2), SAPT+VKA (6.7), DAPT (15.7), DOAC (1.3), VKA (16), Triple Therapy (1.9) | The device manufacturer recommended ASA (80–100 mg) and clopidogrel (75 mg) daily for 1–3 months post-LAAO, followed by ASA (80–100 mg) alone for at least 3 months. However, the choice and duration of therapy were tailored to patient history, LAAO indications, and physician preference. |
| **Bösche 2015**(50) | x |  |  |  | x |  |  | DOAC (40), DAPT (60) | Patients with contraindications to DOACs were prescribed DAPT (aspirin 100 mg/day + clopidogrel 75 mg/day) for 6 months, followed by lifelong aspirin (100 mg/day). Patients eligible for OAC received DOACs (dabigatran or rivaroxaban) for ≥45 days, as decided by the operator. After 45 days, DOACs were typically replaced with DAPT until 6 months post-implantation, after which clopidogrel was discontinued, and aspirin monotherapy was continued. |
| **Wiebe 2015**(51) |  |  |  |  | x |  | x | DAPT (41.8), SAPT+VKA (58.2) | In patients without anticoagulation contraindications, VKAs were given for 45 days with heparin bridging until the INR reached 2.0–3.0. Aspirin (100 mg/day) was initiated the day before implantation and continued lifelong. Clopidogrel was added at the 45-day follow-up and continued until the 6-month follow-up. Patients ineligible for anticoagulation were treated with lifelong aspirin (100 mg/day) starting the day before implantation and clopidogrel (75 mg/day) from implantation to 6 months. |
| **Meincke 2013**(52) |  |  | x |  | x |  |  | DAPT (88.3), VKA (11.6) | When OAT was feasible, the PROTECT-AF protocol was followed: warfarin for 45 days, then DAPT (aspirin 100 mg/day + clopidogrel 75 mg/day) for 6 months, followed by aspirin monotherapy. In patients with contraindications to warfarin, DAPT was initiated and maintained for at least 3 months. Device evaluation via echocardiography was performed at 3 and 6 months. Clopidogrel was discontinued after 3 months for high bleeding risk patients and after 6 months for those with low to moderate bleeding risk if the device was properly positioned and no thrombi were present. |

### **Supplementary Table 7.** Endpoint events, sample size, and follow-up duration, stratified by each post-LAAO antithrombotic therapy and by each study

| **Author - Year** | **Follow-up** | **Endpoints** | **SAPT** | | **DAPT** | | **VKA** | | **standard-dose DOAC** | | **VKA + SAPT** | | **DOAC + SAPT** | | **low-dose DOAC** | |
| --- | --- | --- | --- | --- | --- | --- | --- | --- | --- | --- | --- | --- | --- | --- | --- | --- |
|  |  |  | **Events** | **Sample Size** | **Events** | **Sample Size** | **Events** | **Sample Size** | **Events** | **Sample Size** | **Events** | **Sample Size** | **Events** | **Sample Size** | **Events** | **Sample Size** |
| **Freixa 2024** | 1 month | DRT | - | - | 4 | 46 | - | - | - | - | - | - | - | - | 0 | 44 |
|  | 3 months | major bleeding | - | - | 13 | 46 | - | - | - | - | - | - | - | - | 2 | 44 |
|  |  | thromboembolism | - | - | 4 | 46 | - | - | - | - | - | - | - | - | 0 | 44 |
| **Reinhardt 2024** | 45 days | DRT | 8 | 1425 | 22 | 4342 | 6 | 1310 | 35 | 12168 | 16 | 4504 | 89 | 28667 | - | - |
|  | 6 months | major bleeding | 30 | 1425 | 123 | 4342 | 16 | 1310 | 167 | 12168 | 97 | 4504 | 732 | 28667 | - | - |
|  |  | thromboembolism | 4 | 1425 | 21 | 4342 | 4 | 1310 | 34 | 12168 | 20 | 4504 | 85 | 28667 | - | - |
|  |  | all-cause mortality | 21 | 1425 | 38 | 4342 | 14 | 1310 | 66 | 12168 | 37 | 4504 | 172 | 28667 | - | - |
| **Mesnier 2024** | 3 months | major bleeding | 14 | 413 | 43 | 645 | 3 | 148 | 7 | 327 | - | - | - | - | - | - |
| **Moliner-Abós 2024** | 46.2 months | major bleeding | 3 | 32 | - | - | - | - | 0 | 3 | - | - | - | - | - | - |
| **Li 2023** | 12 months | DRT | - | - | 12 | 129 | - | - | - | - | - | - | - | - | 10 | 240 |
|  | 1 year | thromboembolism | - | - | 14 | 140 | - | - | - | - | - | - | - | - | 14 | 240 |
| **Zhang 2023** | 2 months | DRT | - | - | - | - | - | - | 2 | 120 | - | - | - | - | 3 | 83 |
|  | 1 year | major bleeding | - | - | - | - | - | - | 5 | 120 | - | - | - | - | 1 | 83 |
|  |  | thromboembolism | - | - | - | - | - | - | 2 | 120 | - | - | - | - | 2 | 83 |
| **Zhou 2023** | 45 days | DRT | - | - | - | - | 3 | 115 | - | - | - | - | - | - | 3 | 342 |
|  | 2.7 years | major bleeding | - | - | - | - | 5 | 115 | - | - | - | - | - | - | 3 | 342 |
|  |  | thromboembolism | - | - | - | - | 4 | 115 | - | - | - | - | - | - | 8 | 342 |
| **Bangash 2023** | 12 months | DRT | - | - | 0 | 6 | - | - | 0 | 42 | - | - | - | - | 0 | 55 |
|  |  | major bleeding | - | - | 0 | 6 | - | - | 0 | 42 | - | - | - | - | 0 | 55 |
|  |  | thromboembolism | - | - | 0 | 6 | - | - | 0 | 42 | - | - | - | - | 0 | 55 |
|  |  | all-cause mortality | - | - | 0 | 6 | - | - | 0 | 42 | - | - | - | - | 0 | 55 |
| **Kailey 2023** | 3 months | DRT | 0 | 26 | 0 | 2 | 0 | 9 | 0 | 22 | - | - | - | - | - | - |
|  |  | major bleeding | 0 | 26 | 2 | 2 | 0 | 9 | 1 | 22 | - | - | - | - | - | - |
|  |  | thromboembolism | 0 | 26 | 0 | 2 | 0 | 9 | 0 | 22 | - | - | - | - | - | - |
|  |  | all-cause mortality | 0 | 26 | 1 | 2 | 0 | 9 | 2 | 22 | - | - | - | - | - | - |
| **Zadori 2023** | 2.7 ± 2.5 years | thromboembolism | 0 | 61 | 4 | 61 | 0 | 1 | 0 | 4 | - | - | - | - | - | - |
| **Maarse 2023** | 3 months | DRT | 0 | 48 | 0 | 46 | - | - | 3 | 113 | - | - | 0 | 16 | - | - |
| **Margonato 2023** | 47.2 months | DRT | - | - | 2 | 31 | 0 | 30 | 0 | 36 | - | - | - | - | - | - |
|  |  | major bleeding | - | - | 2 | 31 | 1 | 30 | 3 | 36 | - | - | - | - | - | - |
|  |  | thromboembolism | - | - | 7 | 31 | 2 | 30 | 0 | 36 | - | - | - | - | - | - |
| **Ge 2022** | 6 weeks | DRT | - | - | - | - | 1 | 46 | - | - | - | - | - | - | 6 | 38 |
|  | 12 months | major bleeding | - | - | - | - | 1 | 46 | - | - | - | - | - | - | 0 | 38 |
|  |  | thromboembolism | - | - | - | - | 1 | 46 | - | - | - | - | - | - | 0 | 38 |
| **Fu 2022** | 45 days | DRT | - | - | - | - | 3 | 77 | - | - | - | - | - | - | 2 | 291 |
|  | 3 months | major bleeding | - | - | - | - | 0 | 77 | - | - | - | - | - | - | 1 | 291 |
|  |  | thromboembolism | - | - | - | - | 1 | 77 | - | - | - | - | - | - | 0 | 291 |
|  |  | all-cause mortality | - | - | - | - | 0 | 77 | - | - | - | - | - | - | 1 | 291 |
| **Vignali 2022** | 12 months | DRT | 0 | 49 | 1 | 74 | - | - | - | - | - | - | - | - | - | - |
|  | 32 months | major bleeding | 9 | 49 | 4 | 74 | - | - | - | - | - | - | - | - | - | - |
|  |  | thromboembolism | 5 | 49 | 5 | 74 | - | - | - | - | - | - | - | - | - | - |
| **Fukunaga 2022** | 45 days | DRT | - | - | 0 | 6 | - | - | - | - | 2 | 27 | 1* | 22 | - | - |
|  | 1 year | major bleeding | - | - | 1 | 6 | - | - | - | - | 3 | 27 | 5 | 22 | - | - |
|  |  | thromboembolism | - | - | 0 | 6 | - | - | - | - | 1 | 27 | 0 | 22 | - | - |
|  |  | all-cause mortality | - | - | 1 | 6 | - | - | - | - | 2 | 27 | 1 | 22 | - | - |
| **Li 2022** | 3 months | DRT | - | - | - | - | 3 | 27 | 5 | 55 | - | - | 1 | 78 | - | - |
| **Pivato 2022** | 6 months | DRT | 0 | 3 | 0 | 44 | - | - | 0 | 3 | - | - | - | - | - | - |
|  |  | major bleeding | 0 | 3 | 1 | 44 | - | - | 0 | 3 | - | - | - | - | - | - |
|  |  | thromboembolism | 0 | 3 | 0 | 44 | - | - | 0 | 3 | - | - | - | - | - | - |
| **Su 2022** | 1 month | major bleeding | 3 | 50 | 3 | 154 | 2 | 371 | 22 | 2056 | 1 | 35 | 3 | 390 | - | - |
|  |  | thromboembolism | 2 | 50 | 1 | 154 | 0 | 371 | 4 | 2056 | 0 | 35 | 2 | 390 | - | - |
|  |  | all-cause mortality | 1 | 50 | 1 | 154 | 1 | 371 | 4 | 2056 | 0 | 35 | 0 | 390 | - | - |
| **Cepas-Guillen 2021** | 3 months | DRT | 2 | 26 | 3 | 73 | - | - | - | - | - | - | - | - | 0 | 40 |
|  |  | major bleeding | 0 | 26 | 7 | 73 | - | - | - | - | - | - | - | - | 0 | 40 |
|  |  | thromboembolism | 0 | 26 | 1 | 73 | - | - | - | - | - | - | - | - | 0 | 40 |
|  |  | all-cause mortality | 1 | 26 | 2 | 73 | - | - | - | - | - | - | - | - | 2 | 40 |
| **Della Rocca 2021** | 45 days | DRT | - | - | - | - | - | - | - | - | - | - | 12 | 357 | 0 | 198 |
|  | 14 months | major bleeding | - | - | - | - | - | - | - | - | - | - | 14 | 357 | 1 | 198 |
|  |  | thromboembolism | - | - | - | - | - | - | - | - | - | - | 11 | 357 | 1 | 198 |
|  |  | all-cause mortality | - | - | - | - | - | - | - | - | - | - | 22 | 357 | 9 | 198 |
| **Schrag 2021** | 45 days | thromboembolism | 1 | 8 | 0 | 2 | 0 | 5 | 0 | 2 | - | - | - | - | 0 | 2 |
|  | 25 months | all-cause mortality | 1 | 8 | 0 | 2 | 0 | 5 | 0 | 2 | - | - | - | - | 0 | 2 |
| **Briosa e Gala 2021** | 2 months | DRT | 3 | 20 | 5 | 158 | - | - | - | - | - | - | - | - | - | - |
| **Casu 2021** | 16.1 months | major bleeding | 1 | 35 | 2 | 58 | - | - | - | - | - | - | - | - | - | - |
|  |  | thromboembolism | 1 | 35 | 1 | 58 | - | - | - | - | - | - | - | - | - | - |
|  |  | all-cause mortality | 1 | 35 | 1 | 58 | - | - | - | - | - | - | - | - | - | - |
| **Chen 2021** | 45 days | DRT | - | - | - | - | 3 | 170 | 4 | 164 | - | - | - | - | - | - |
|  | 868 days | major bleeding | - | - | - | - | 1 | 170 | 0 | 164 | - | - | - | - | - | - |
|  |  | thromboembolism | - | - | - | - | 0 | 170 | 2 | 164 | - | - | - | - | - | - |
|  |  | all-cause mortality | - | - | - | - | 6 | 155 | 2 | 166 | - | - | - | - | - | - |
| **Faroux 2021** | 90 days | DRT | - | - | 4 | 154 | - | - | 0 | 74 | - | - | - | - | - | - |
|  | 22 months | major bleeding | - | - | 14 | 190 | - | - | 3 | 95 | - | - | - | - | - | - |
|  |  | thromboembolism | - | - | 2 | 190 | - | - | 1 | 95 | - | - | - | - | - | - |
|  |  | all-cause mortality | - | - | 7 | 190 | - | - | 1 | 95 | - | - | - | - | - | - |
| **Mazzone 2021** | 90 days | DRT | 0 | 6 | 2 | 186 | 0 | 26 | 1 | 13 | - | - | 0 | 12 | - | - |
|  | 420 days | major bleeding | 1 | 6 | 3 | 186 | 1 | 26 | 0 | 13 | - | - | 1 | 12 | - | - |
|  |  | thromboembolism | 0 | 6 | 3 | 186 | 1 | 26 | 1 | 13 | - | - | 2 | 12 | - | - |
|  |  | all-cause mortality | 0 | 6 | 12 | 186 | 1 | 26 | 0 | 13 | - | - | 0 | 12 | - | - |
| **Tjoe 2021** | 45-day | DRT | - | - | - | - | - | - | 4 | 71 | 2 | 59 | 1 | 81 | - | - |
|  | 1 year | major bleeding | - | - | - | - | - | - | 2 | 67 | 2 | 57 | 1 | 62 | - | - |
|  |  | thromboembolism | - | - | - | - | - | - | 0 | 71 | 0 | 60 | 0 | 78 | - | - |
|  |  | all-cause mortality | - | - | - | - | - | - | 0 | 71 | 1 | 60 | 0 | 78 | - | - |
| **Zhu 2021** | 45 days | DRT | - | - | - | - | 2 | 30 | 1 | 40 | - | - | - | - | - | - |
|  |  | major bleeding | - | - | - | - | 1 | 30 | 0 | 40 | - | - | - | - | - | - |
|  |  | thromboembolism | - | - | - | - | 0 | 30 | 1 | 40 | - | - | - | - | - | - |
| **Duthoit 2020** | 3 months | DRT | - | - | 2 | 33 | - | - | - | - | - | - | - | - | 0 | 71 |
|  |  | major bleeding | - | - | 7 | 33 | - | - | - | - | - | - | - | - | 10 | 71 |
|  |  | thromboembolism | - | - | 0 | 33 | - | - | - | - | - | - | - | - | 1 | 71 |
|  |  | all-cause mortality | - | - | 0 | 33 | - | - | - | - | - | - | - | - | 1 | 71 |
| **Asmarats 2020** | 45 days | DRT | 2 | 17 | 3 | 31 | 0 | 4 | 0 | 26 | - | - | - | - | - | - |
| **Barocelli 2020** | 2.32 years | major bleeding | 2 | 14 | 3 | 44 | - | - | - | - | - | - | - | - | - | - |
|  |  | thromboembolism | 0 | 14 | 4 | 44 | - | - | - | - | - | - | - | - | - | - |
| **Darmon 2020** | 90 days | DRT | 0 | 72 | 0 | 57 | - | - | - | - | 0 | 1 | - | - | - | - |
|  | 20.6 months | major bleeding | 0 | 72 | 0 | 57 | - | - | - | - | 0 | 1 | - | - | - | - |
|  |  | thromboembolism | 0 | 72 | 0 | 57 | - | - | - | - | 0 | 1 | - | - | - | - |
|  |  | all-cause mortality | 2 | 72 | 1 | 57 | - | - | - | - | 0 | 1 | - | - | - | - |
| **Patti 2020** | 90 days | DRT | 4 | 280 | 3 | 330 | - | - | - | - | - | - | - | - | - | - |
|  | 12 months | major bleeding | 8 | 280 | 22 | 330 | - | - | - | - | - | - | - | - | - | - |
|  |  | thromboembolism | 5 | 280 | 7 | 330 | - | - | - | - | - | - | - | - | - | - |
|  |  | all-cause mortality | 17 | 280 | 18 | 330 | - | - | - | - | - | - | - | - | - | - |
| **Zhang 2020** | 45 days | DRT | - | - | - | - | 1 | 23 | 1 | 26 | - | - | - | - | - | - |
| **Şahiner 2019** | 180 days | DRT | 0 | 2 | 0 | 52 | - | - | - | - | - | - | - | - | 0 | 5 |
|  | 21 months | major bleeding | 0 | 2 | 1 | 52 | - | - | - | - | - | - | - | - | 0 | 5 |
|  |  | thromboembolism | 0 | 2 | 2 | 52 | - | - | - | - | - | - | - | - | 0 | 5 |
| **Cheung 2019** | 45 days | DRT | - | - | 1 | 95 | - | - | - | - | 4 | 61 | - | - | - | - |
|  | 28.3 months | major bleeding | - | - | 3 | 95 | - | - | - | - | 4 | 67 | - | - | - | - |
|  |  | thromboembolism | - | - | 3 | 95 | - | - | - | - | 1 | 67 | - | - | - | - |
|  |  | all-cause mortality | - | - | 10 | 93 | - | - | - | - | 4 | 65 | - | - | - | - |
| **Cohen 2019** | 90 days | DRT | - | - | - | - | 0 | 45 | 0 | 52 | - | - | - | - | - | - |
|  | 8 months | major bleeding | - | - | - | - | 2 | 45 | 3 | 52 | - | - | - | - | - | - |
|  |  | thromboembolism | - | - | - | - | 1 | 45 | 0 | 52 | - | - | - | - | - | - |
|  |  | all-cause mortality | - | - | - | - | 2 | 45 | 1 | 52 | - | - | - | - | - | - |
| **Fauchier 2018** | 90 days | DRT | 11 | 102 | 1 | 82 | - | - | - | - | - | - | - | - | - | - |
| **Landmesser 2018** | 1 year | DRT | 2 | 242 | 10 | 591 | - | - | - | - | - | - | - | - | - | - |
|  |  | major bleeding | 16 | 242 | 52 | 619 | - | - | - | - | - | - | - | - | - | - |
| **Peyrol 2018** | 2 months | DRT | 0 | 10 | 1 | 27 | - | - | - | - | - | - | - | - | - | - |
|  | 15.5 months | thromboembolism | 0 | 10 | 0 | 27 | - | - | - | - | - | - | - | - | - | - |
|  |  | all-cause mortality | 0 | 10 | 0 | 27 | - | - | - | - | - | - | - | - | - | - |
| **Zhang 2018** | 45 days | DRT | - | - | 1 | 33 | 1 | 33 | 1 | 33 | - | - | - | - | - | - |
|  | 18.7 months | major bleeding | - | - | 0 | 33 | 0 | 33 | 0 | 33 | - | - | - | - | - | - |
|  |  | thromboembolism | - | - | 0 | 33 | 0 | 33 | 0 | 33 | - | - | - | - | - | - |
| **Bergmann 2017** | 92 days | DRT | 2 | 70 | 15 | 605 | 1 | 155 | 0 | 64 | - | - | - | - | 1 | 45 |
|  |  | major bleeding | 2 | 70 | 9 | 605 | 3 | 155 | 0 | 64 | - | - | - | - | 2 | 45 |
|  |  | thromboembolism | 1 | 70 | 3 | 605 | 0 | 155 | 0 | 64 | - | - | - | - | 0 | 45 |
| **da Costa 2017** | 180 days | DRT | 0 | 8 | 0 | 3 | - | - | - | - | - | - | - | - | - | - |
|  | 1 year | major bleeding | 0 | 8 | 0 | 3 | - | - | - | - | - | - | - | - | - | - |
|  |  | thromboembolism | 0 | 8 | 0 | 3 | - | - | - | - | - | - | - | - | - | - |
|  |  | all-cause mortality | 0 | 8 | 0 | 3 | - | - | - | - | - | - | - | - | - | - |
| **Korsholm 2017** | 42 days | DRT | 2 | 94 | 0 | 13 | - | - | - | - | - | - | - | - | - | - |
|  | 2.3 years | major bleeding | 1 | 94 | 1 | 13 | - | - | - | - | - | - | - | - | - | - |
| **Saw 2017** | 88 days | DRT | 3 | 79 | 7 | 159 | - | - | - | - | - | - | - | - | - | - |
| **Enomoto 2016** | 42 days | DRT | - | - | - | - | 1 | 212 | 2 | 214 | - | - | - | - | - | - |
|  | 4 months | major bleeding | - | - | - | - | 1 | 212 | 1 | 214 | - | - | - | - | - | - |
|  |  | thromboembolism | - | - | - | - | 1 | 212 | 1 | 214 | - | - | - | - | - | - |
| **Tung 2016** | 2.4 years | DRT | 0 | 6 | 0 | 11 | - | - | - | - | - | - | - | - | - | - |
|  |  | thromboembolism | 1 | 6 | 0 | 11 | - | - | - | - | - | - | - | - | - | - |
| **Tzikas 2016** | 210 days | DRT | 1 | 78 | 15 | 312 | 0 | 6 | 0 | 2 | - | - | - | - | - | - |
|  | 13 months | major bleeding | 3 | 110 | 9 | 410 | 0 | 6 | 0 | 2 | - | - | - | - | - | - |
|  |  | thromboembolism | 4 | 110 | 5 | 410 | 0 | 6 | 0 | 2 | - | - | - | - | - | - |
|  |  | all-cause mortality | 1 | 110 | 8 | 410 | 1 | 6 | 0 | 2 | - | - | - | - | - | - |
| **Bösche 2015** | 45 days | DRT | - | - | 0 | 27 | - | - | 0 | 18 | - | - | - | - | - | - |
|  | 417 days | major bleeding | - | - | 3 | 27 | - | - | 3 | 18 | - | - | - | - | - | - |
|  |  | thromboembolism | - | - | 0 | 27 | - | - | 0 | 18 | - | - | - | - | - | - |
|  |  | all-cause mortality | - | - | 5 | 27 | - | - | 2 | 18 | - | - | - | - | - | - |
| **Wiebe 2015** | 6 months | DRT | - | - | 2 | 41 | - | - | - | - | 0 | 55 | - | - | - | - |
|  | 3 years | thromboembolism | - | - | 1 | 41 | - | - | - | - | 0 | 55 | - | - | - | - |
| **Meincke 2013** | 45 days | DRT | - | - | 3 | 53 | 0 | 7 | - | - | - | - | - | - | - | - |
|  | 6.3 months | major bleeding | - | - | 0 | 53 | 0 | 7 | - | - | - | - | - | - | - | - |


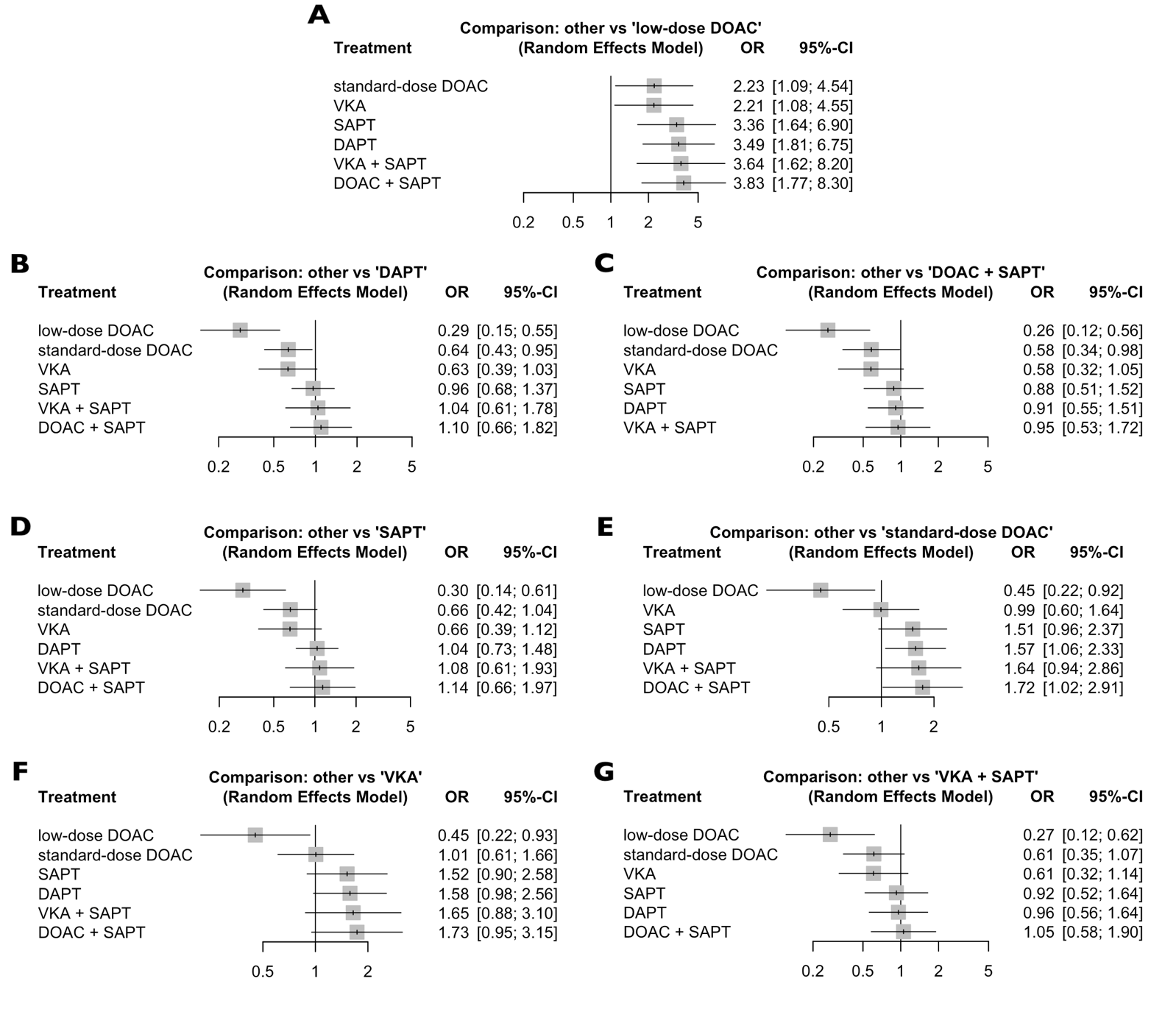


### **Supplemental Figure 2.** Forest plots of random effects network meta-analysis for major bleeding across different reference groups: (A) vs low-dose DOAC, (B) vs DAPT, (C) vs DOAC plus SAPT, (D) vs SAPT, (E) vs standard-dose DOAC, (F) vs VKA, and (G) vs VKA plus SAPT.

Abbreviations: DAPT, dual antiplatelet therapy; DOAC, direct oral anticoagulants; SAPT, single antiplatelet therapy; VKA, vitamin K antagonist.


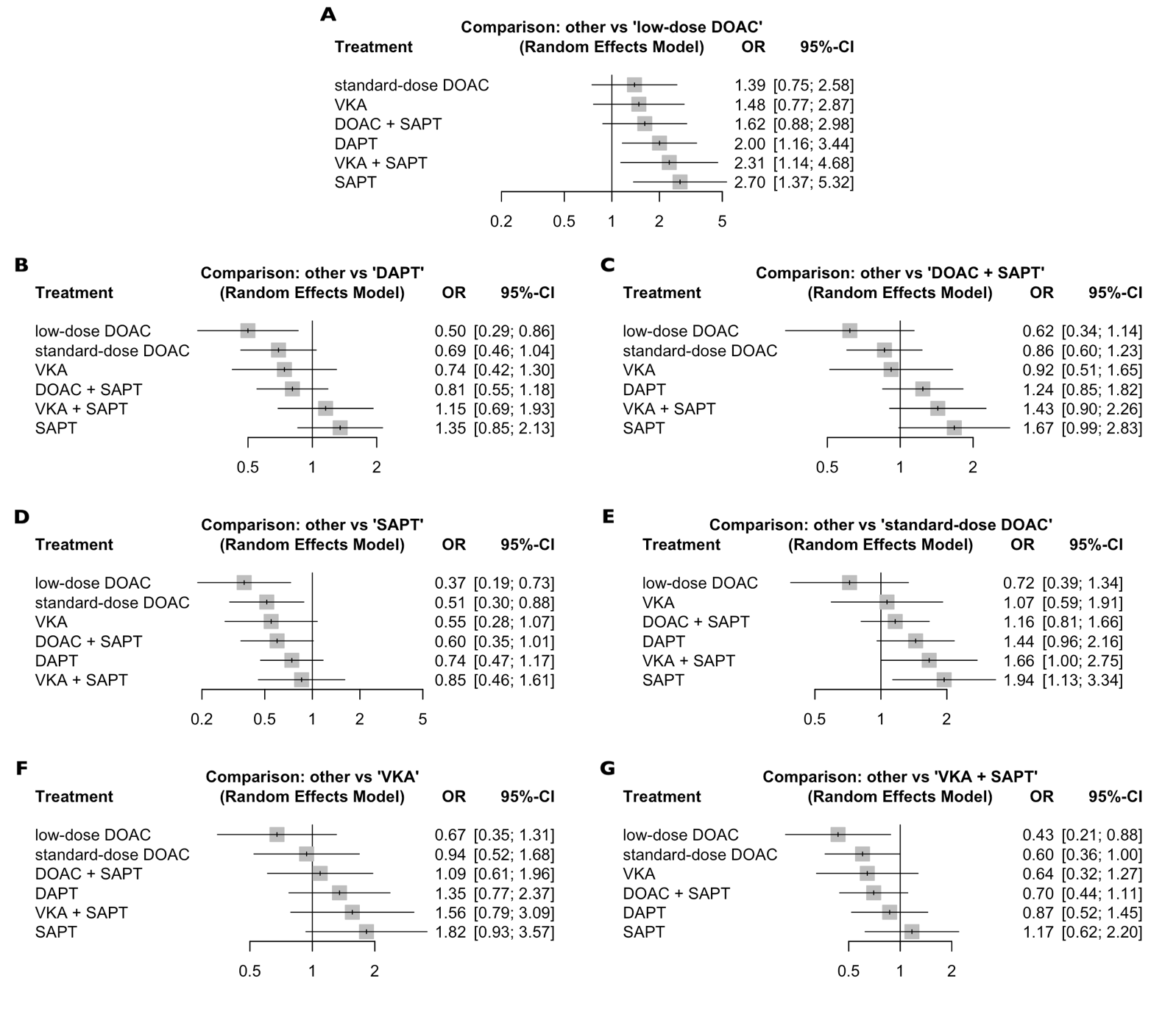


### **Supplemental Figure 3.** Forest plots of random effects network meta-analysis for thromboembolism across different reference groups: (A) vs low-dose DOAC, (B) vs DAPT, (C) vs DOAC plus SAPT, (D) vs SAPT, (E) vs standard-dose DOAC, (F) vs VKA, and (G) vs VKA plus SAPT.

Abbreviations: DAPT, dual antiplatelet therapy; DOAC, direct oral anticoagulants; SAPT, single antiplatelet therapy; VKA, vitamin K antagonist.


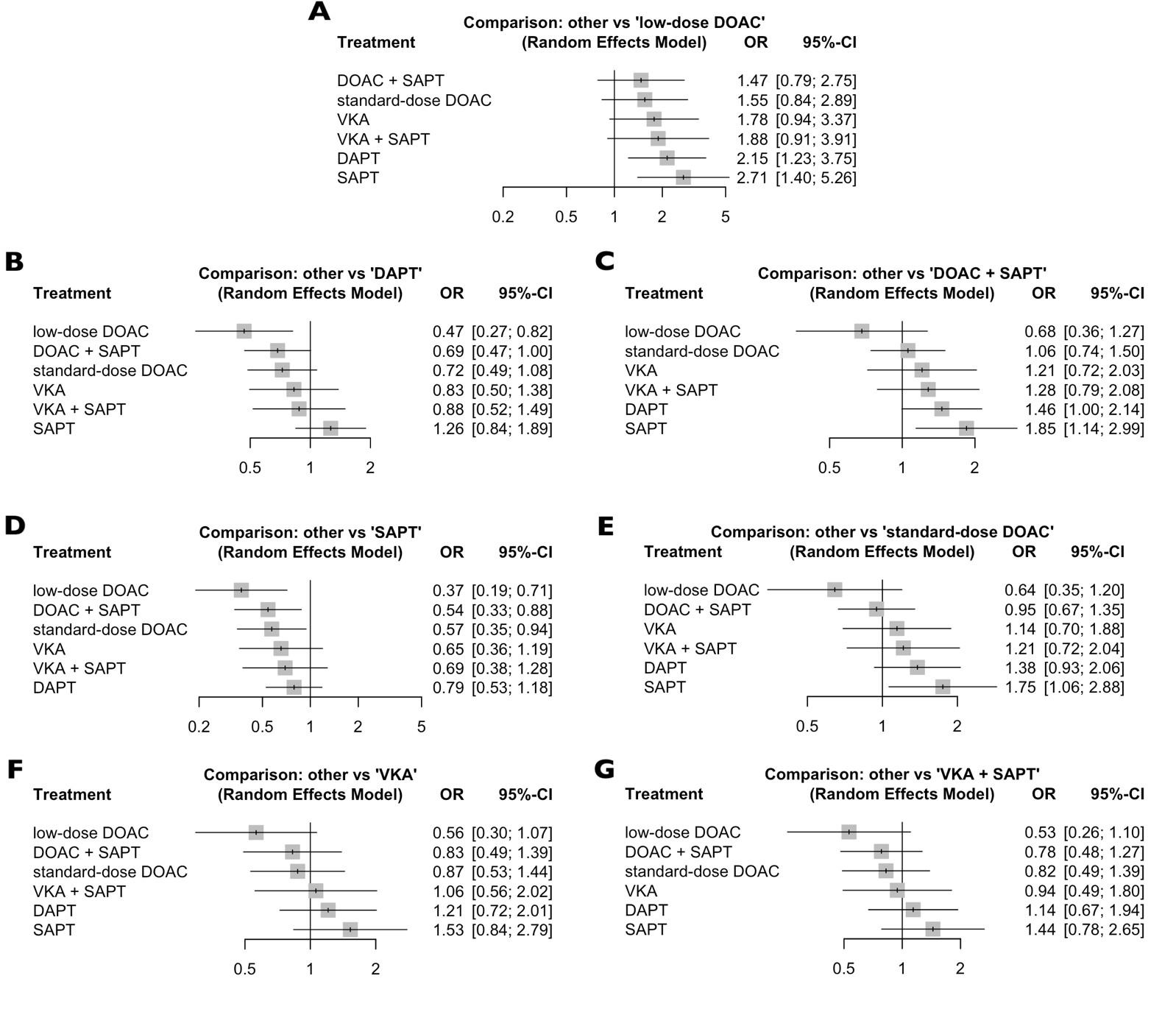


### **Supplementary Figure 4.** Forest plots of random effects network meta-analysis for device-related thrombosis across different reference groups: (A) vs low-dose DOAC, (B) vs DAPT, (C) vs DOAC plus SAPT, (D) vs SAPT, (E) vs standard-dose DOAC, (F) vs VKA, and (G) vs VKA plus SAPT.

Abbreviations: DAPT, dual antiplatelet therapy; DOAC, direct oral anticoagulants; SAPT, single antiplatelet therapy; VKA, vitamin K antagonist.


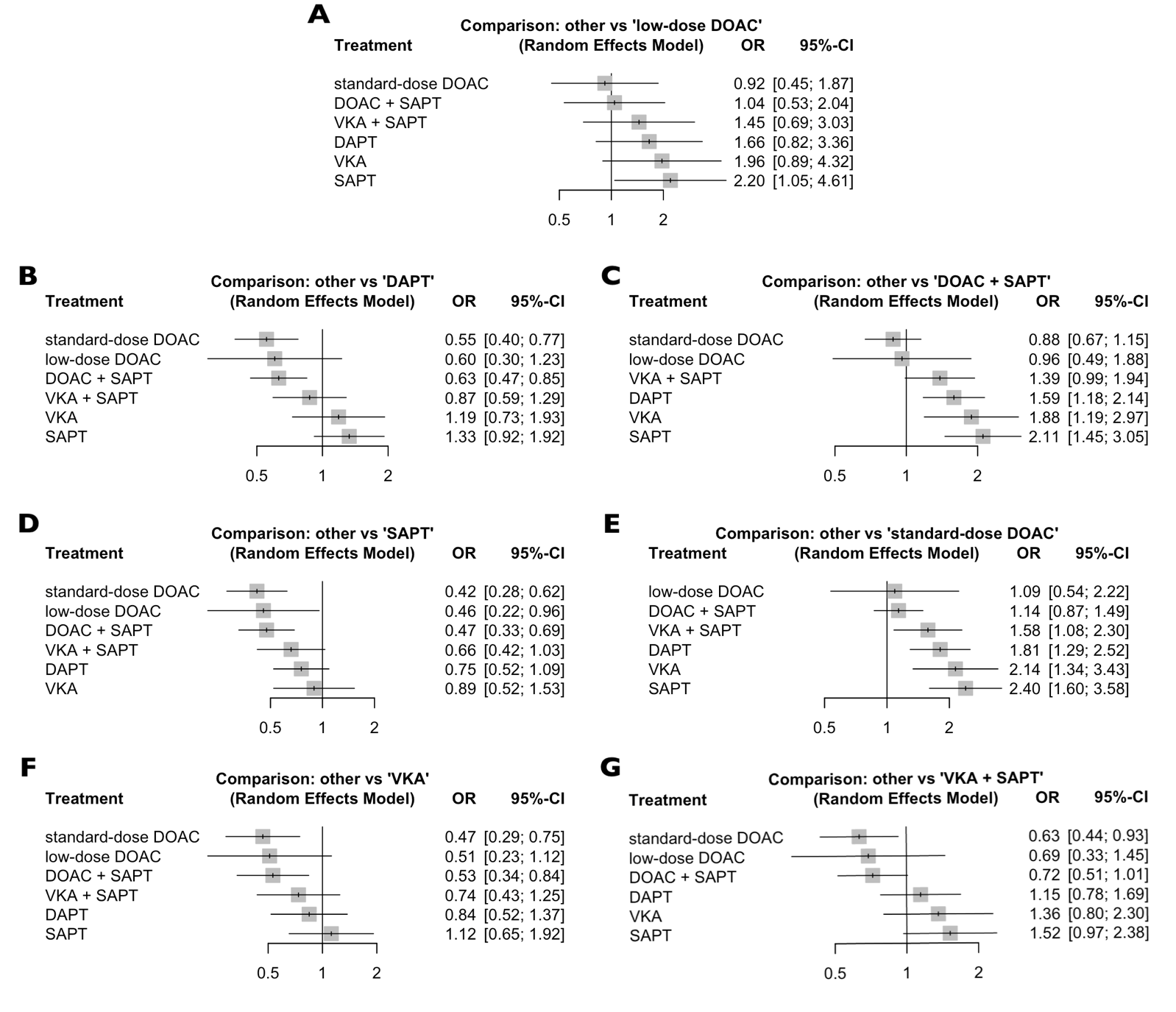


### **Supplemental Figure 5.** Forest plots of random effects network meta-analysis for all-cause mortality across different reference groups: (A) vs low-dose DOAC, (B) vs DAPT, (C) vs DOAC plus SAPT, (D) vs SAPT, (E) vs standard-dose DOAC, (F) vs VKA, and (G) vs VKA plus SAPT.

Abbreviations: DAPT, dual antiplatelet therapy; DOAC, direct oral anticoagulants; SAPT, single antiplatelet therapy; VKA, vitamin K antagonist.


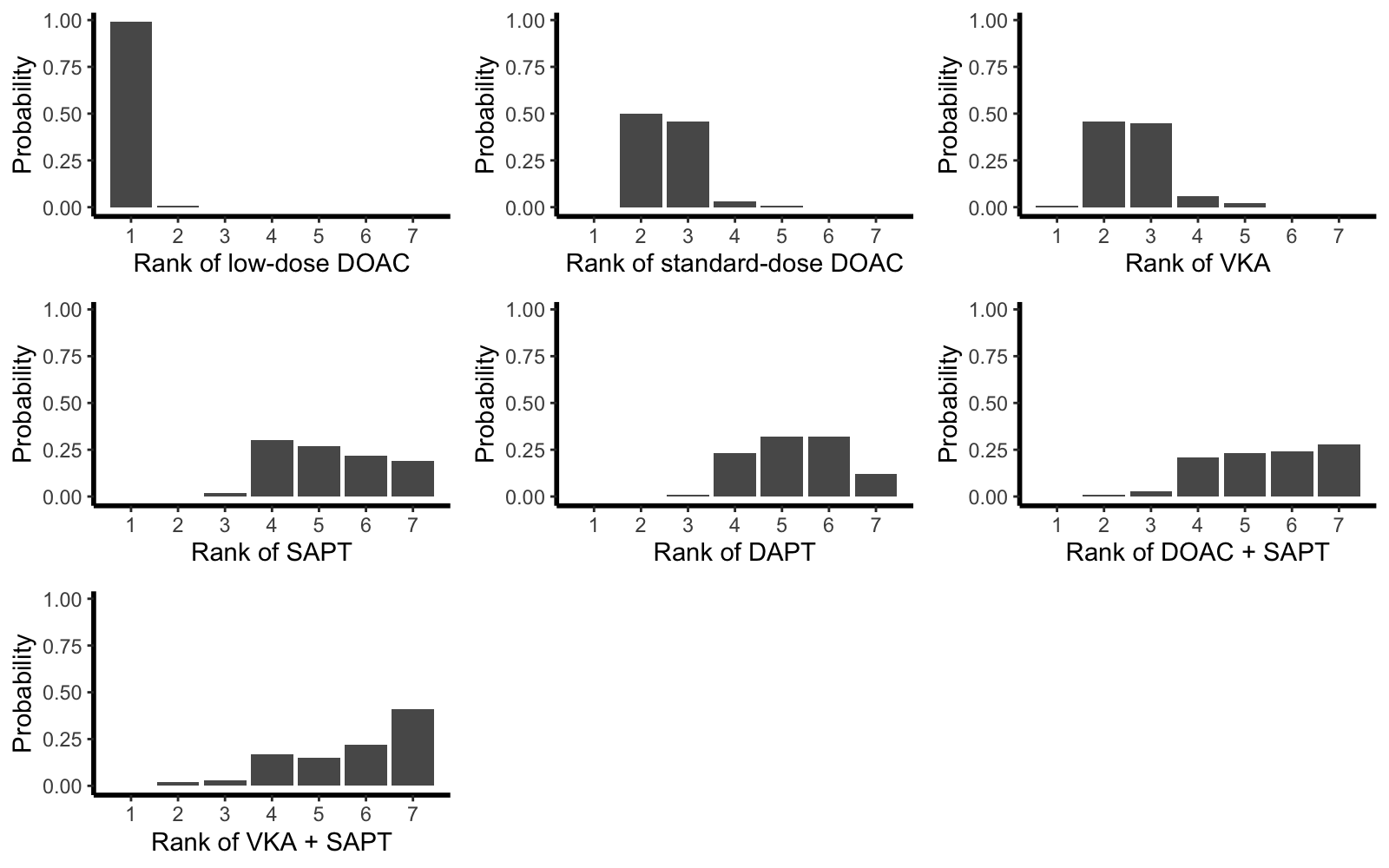


### **Supplemental Figure 6**. Ranking probabilities (rankograms) for the outcome of major bleeding for each antithrombotic strategy.


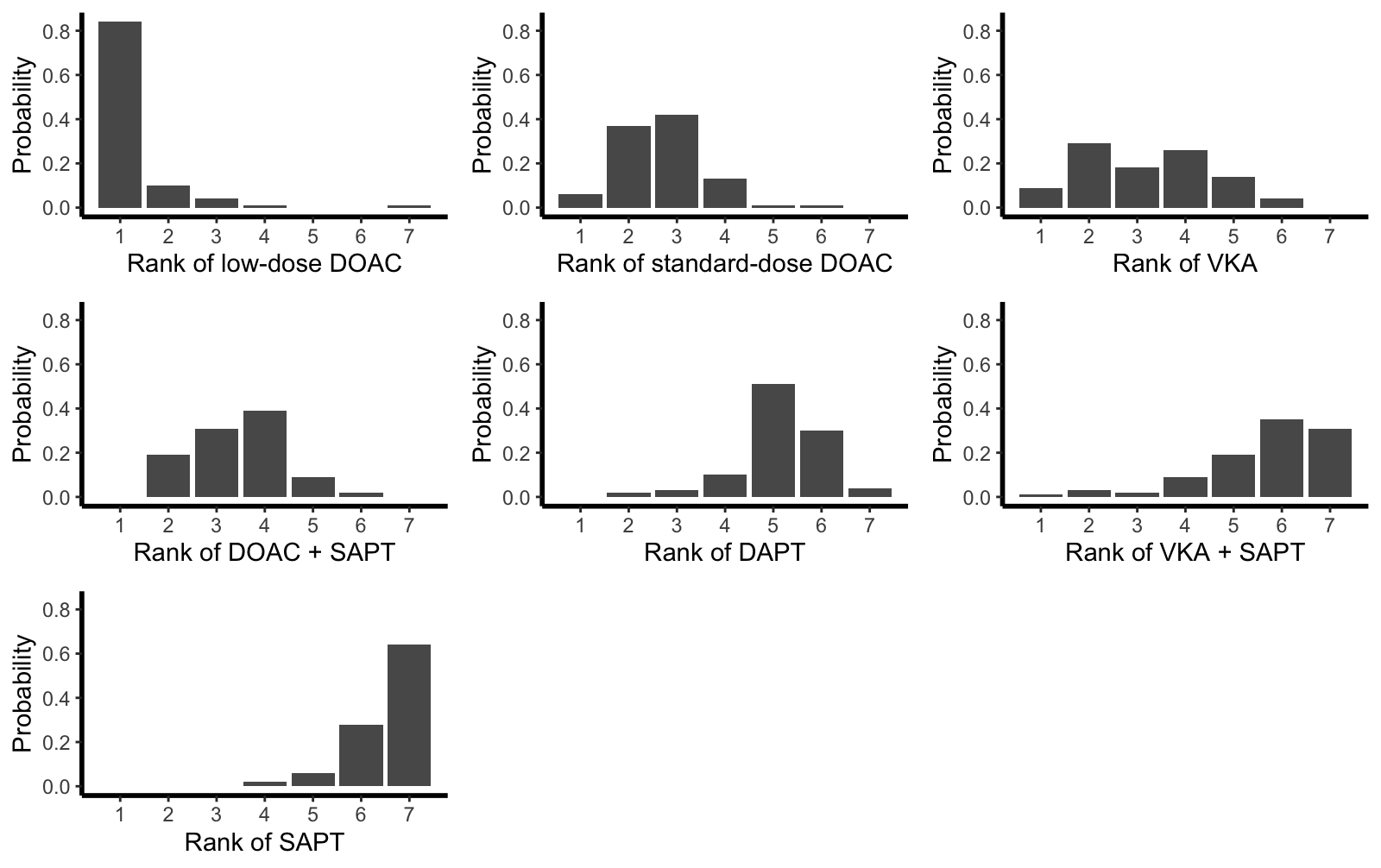


### **Supplemental Figure 7**. Ranking probabilities (rankograms) for the outcome of thromboembolism for each antithrombotic strategy.


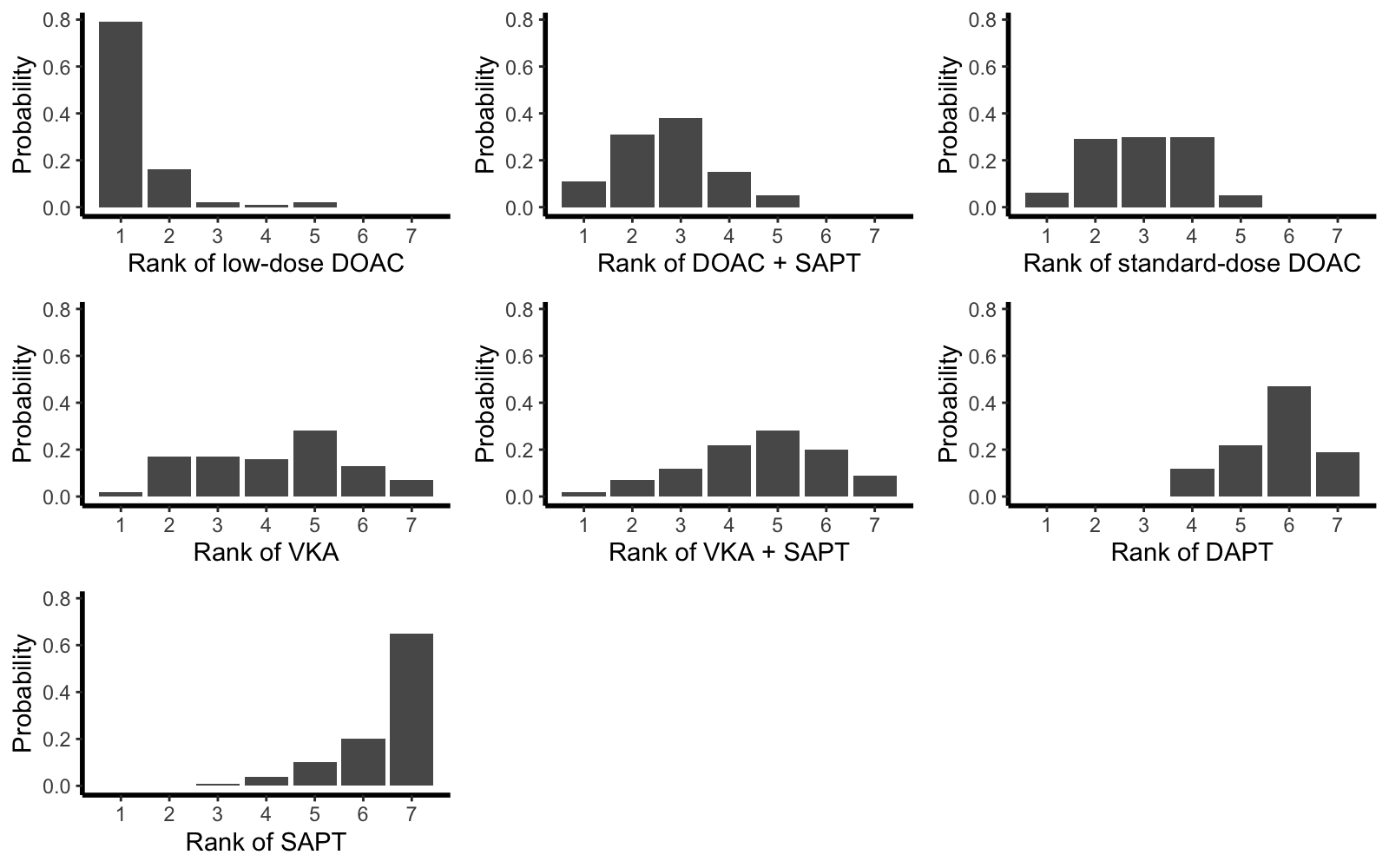


### **Supplemental Figure 8**. Ranking probabilities (rankograms) for the outcome of device-related thrombosis for each antithrombotic strategy.


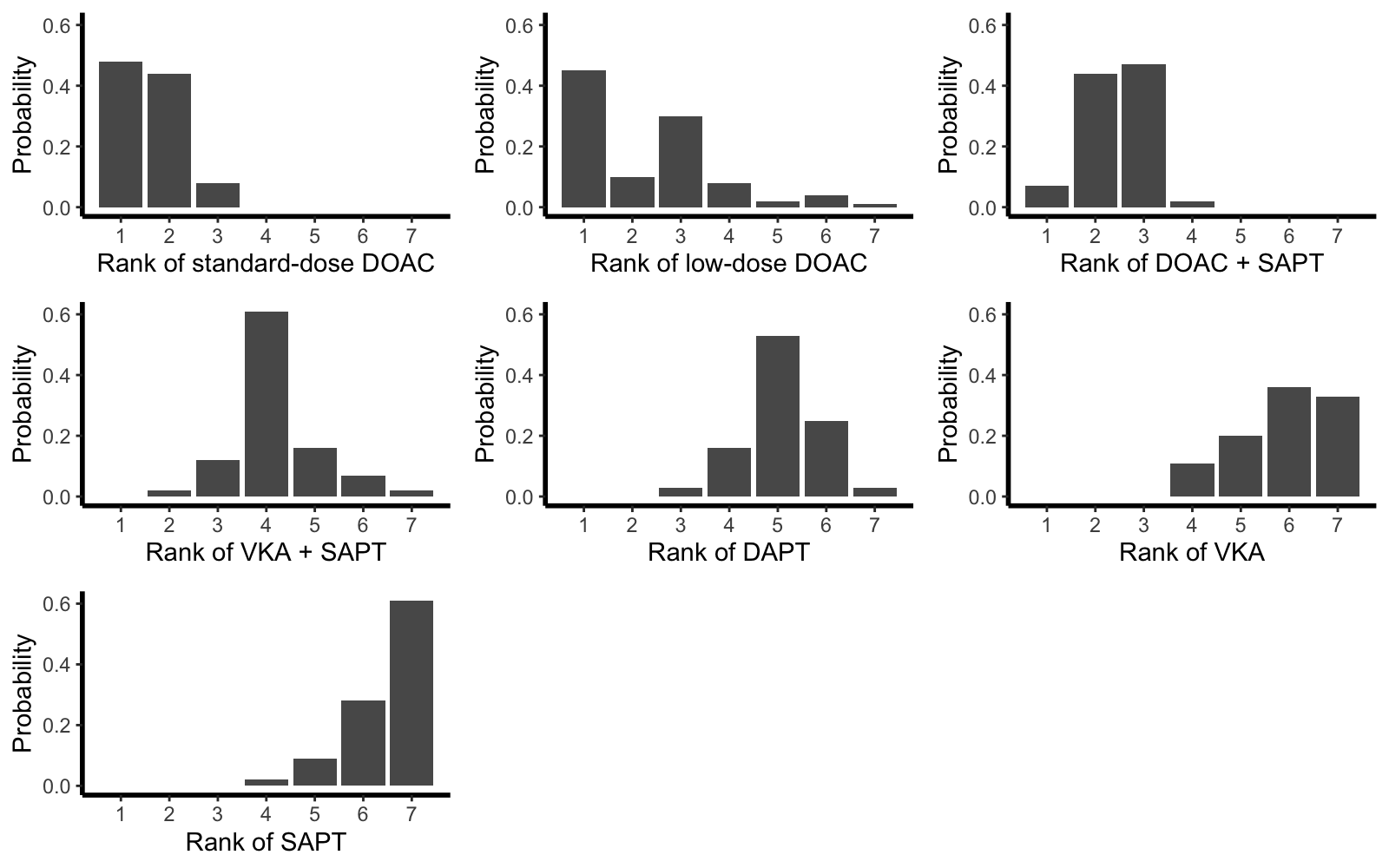


### **Supplemental Figure 9**. Ranking probabilities (rankograms) for the outcome of all-cause mortality for each antithrombotic strategy.


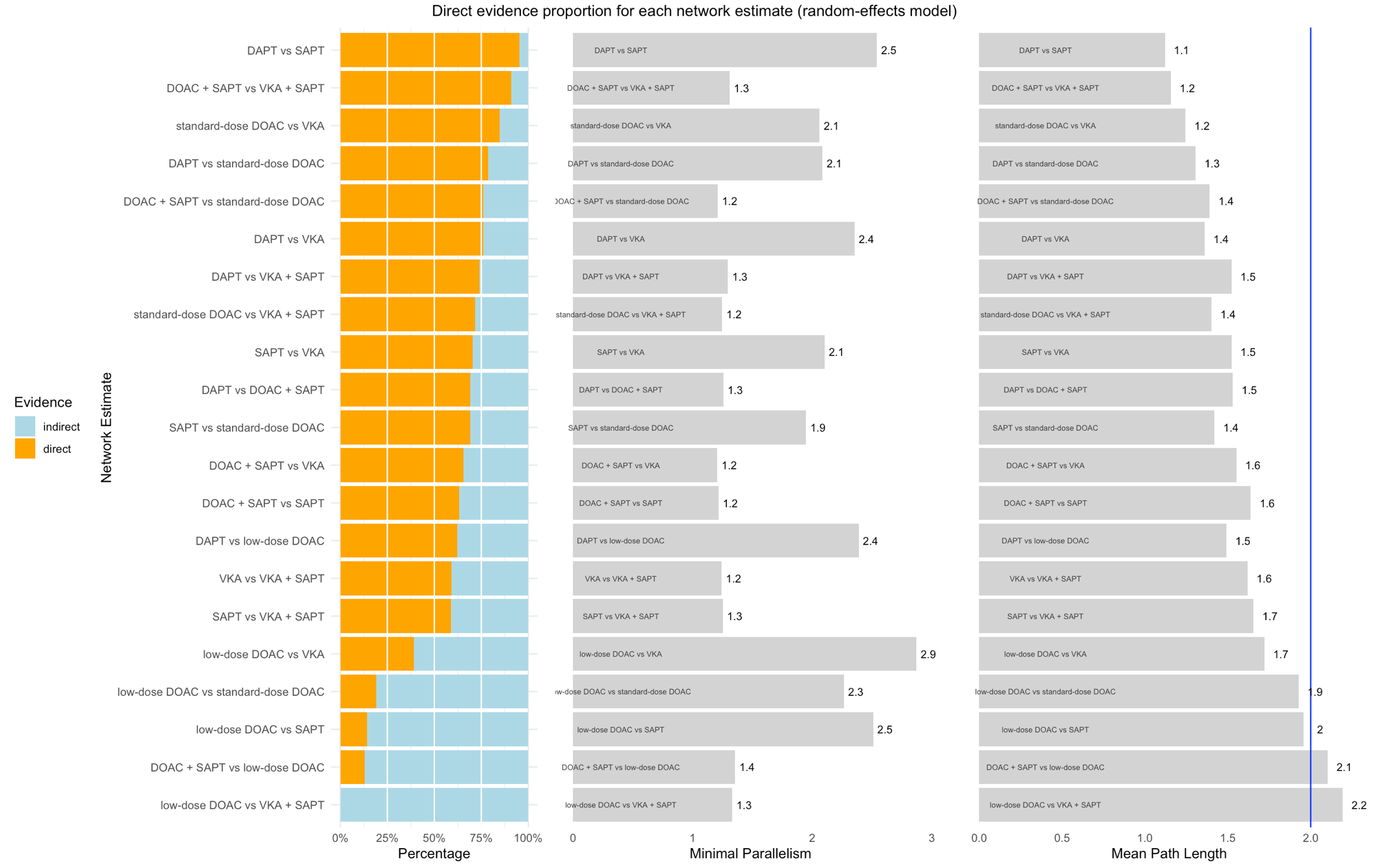


**Supplemental Figure 10**. Plot for direct evidence proportions in the network meta-analysis for major bleeding events: **Direct Evidence Proportion (left)**, this bar chart displays the proportion of direct evidence (orange) contained in each network estimate; **Minimal Parallelism (center)**, this bar chart displays the minimum number of independent paths contributing to the effect estimate on an aggregated level; **Mean Path Length (right)**, this bar chart displays the mean path length, which characterizes the degree of indirectness of an estimate. Higher mean path lengths indicate less reliable estimates, given that more similarity assumptions have to be made when serially combining direct comparisons.


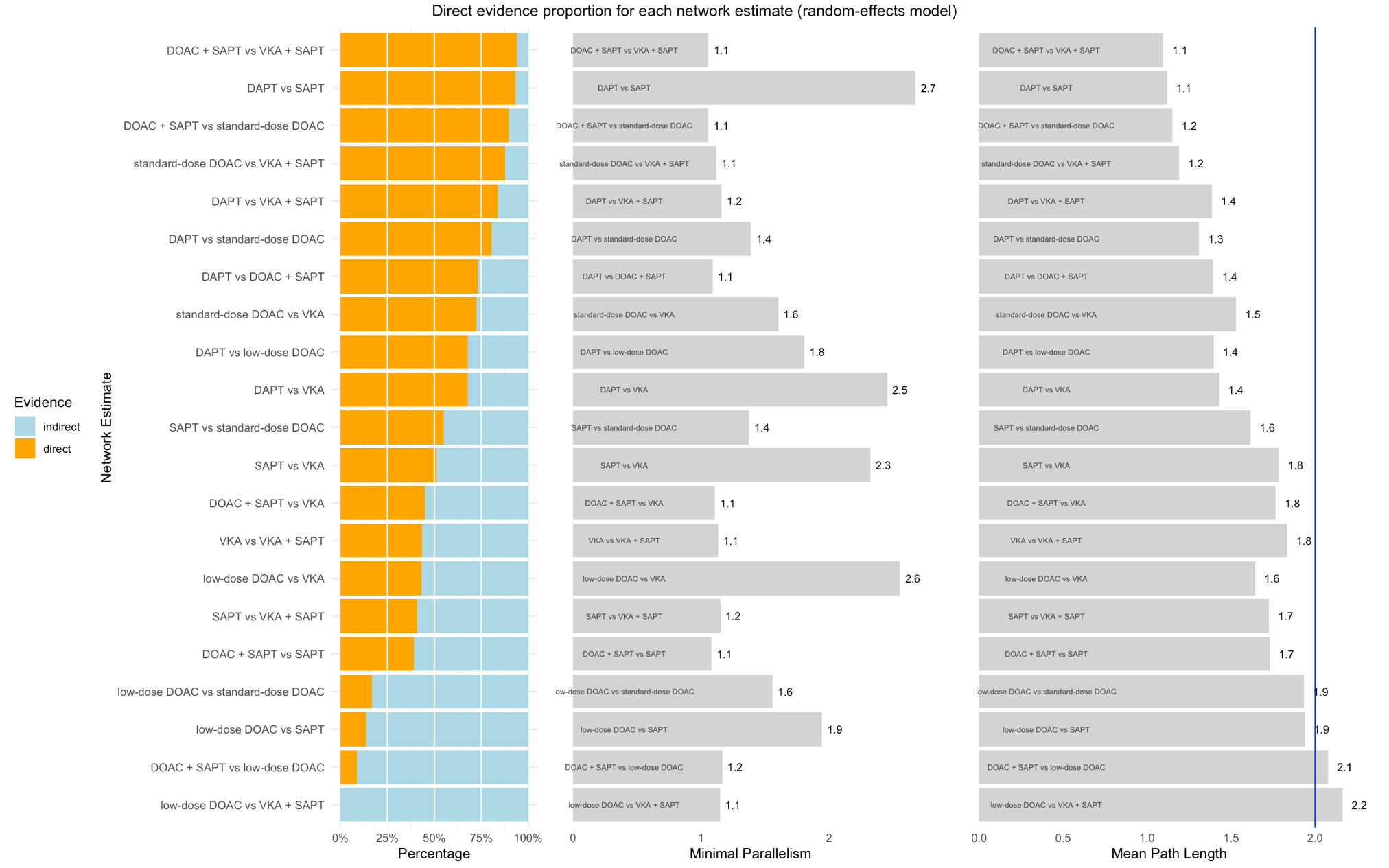


**Supplemental Figure 11**. Plot for direct evidence proportions in the network meta-analysis for thromboembolic events: **Direct Evidence Proportion (left)**, this bar chart displays the proportion of direct evidence (orange) contained in each network estimate; **Minimal Parallelism (center)**, this bar chart displays the minimum number of independent paths contributing to the effect estimate on an aggregated level; **Mean Path Length (right)**, this bar chart displays the mean path length, which characterizes the degree of indirectness of an estimate. Higher mean path lengths indicate less reliable estimates, given that more similarity assumptions have to be made when serially combining direct comparisons.


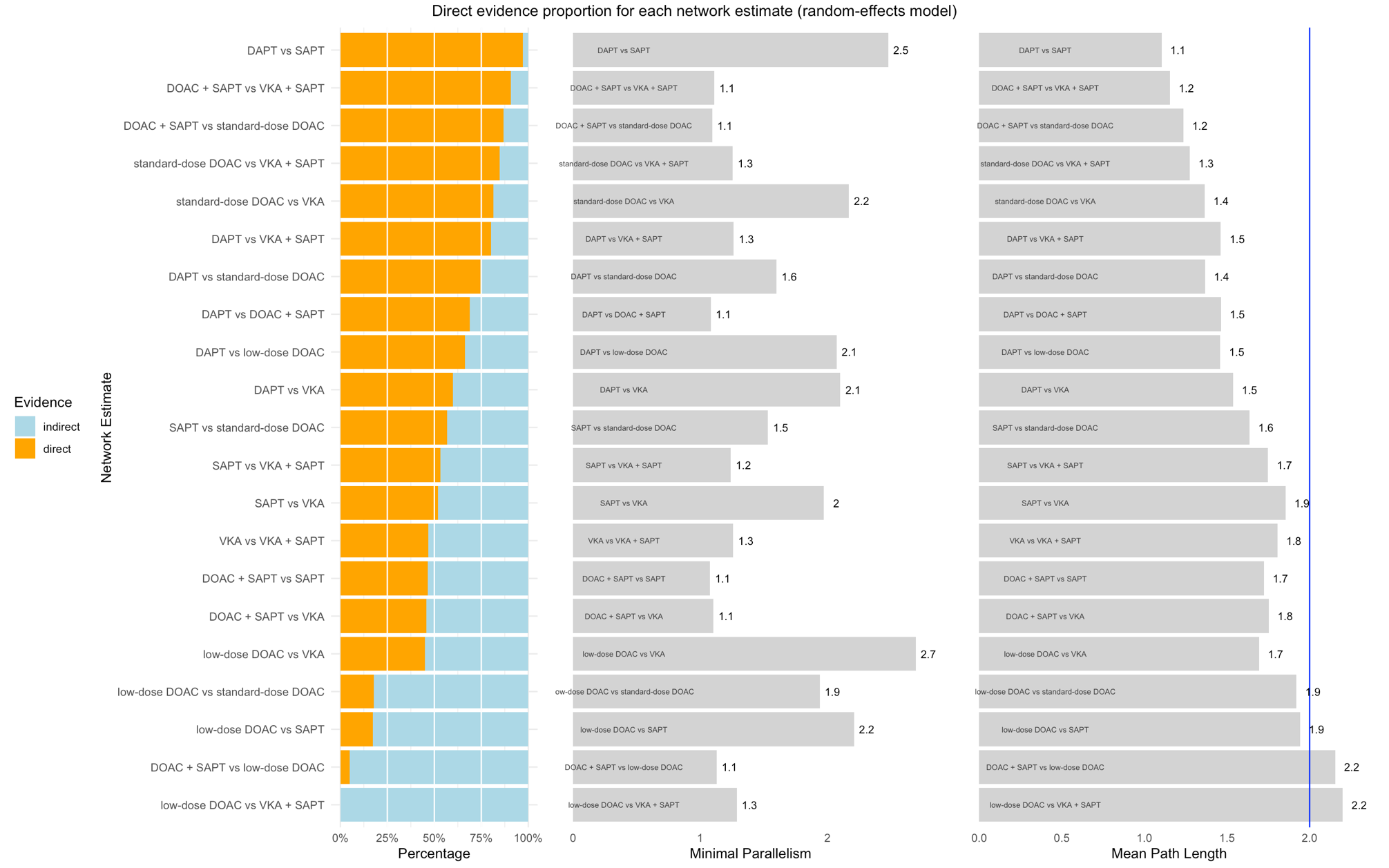


**Supplemental Figure 12**. Plot for direct evidence proportions in the network meta-analysis for device-related thrombosis: **Direct Evidence Proportion (left)**, this bar chart displays the proportion of direct evidence (orange) contained in each network estimate; **Minimal Parallelism (center)**, this bar chart displays the minimum number of independent paths contributing to the effect estimate on an aggregated level; **Mean Path Length (right)**, this bar chart displays the mean path length, which characterizes the degree of indirectness of an estimate. Higher mean path lengths indicate less reliable estimates, given that more similarity assumptions have to be made when serially combining direct comparisons.


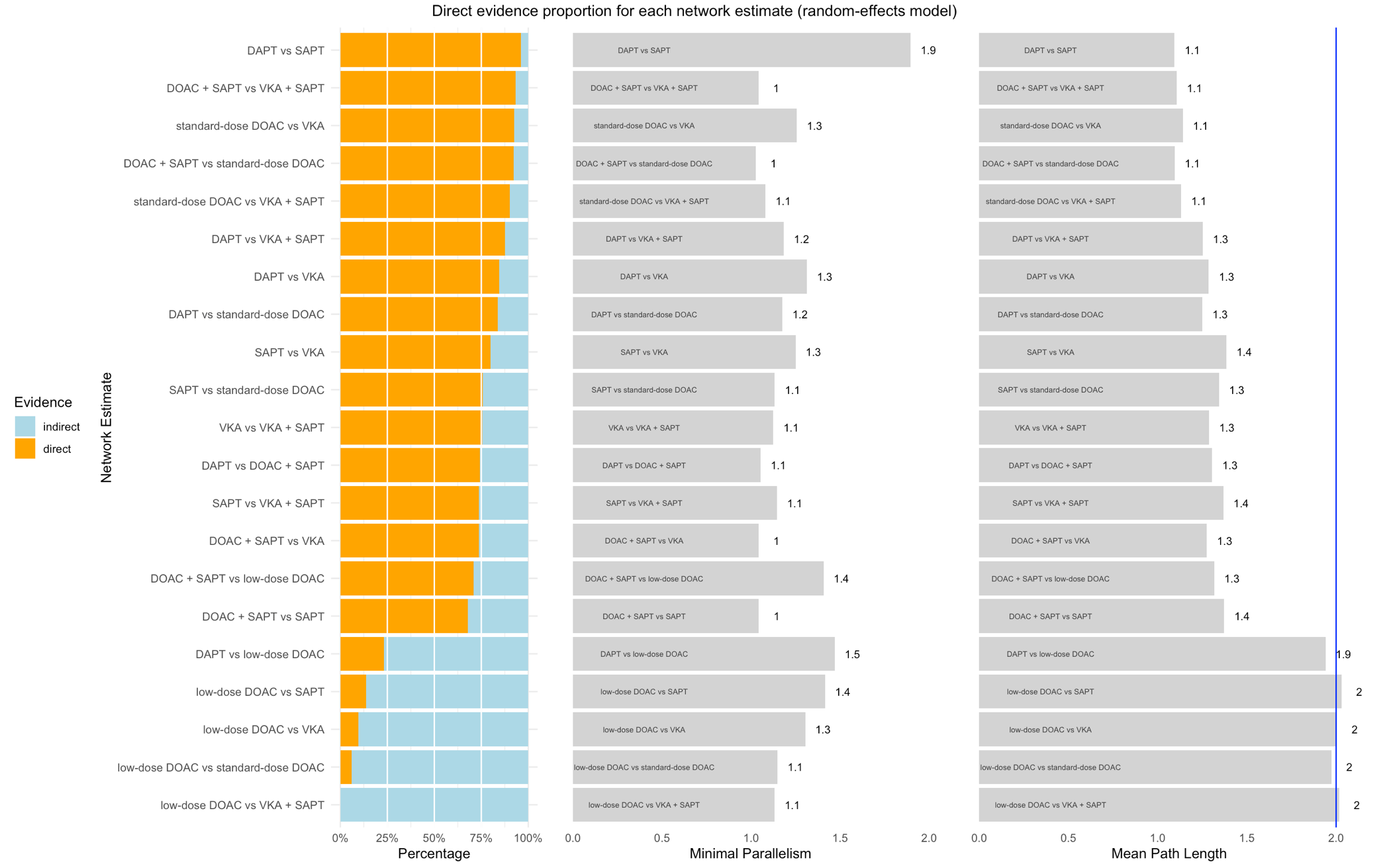


**Supplemental Figure 13**. Plot for direct evidence proportions in the network meta-analysis for all-cause mortality: **Direct Evidence Proportion (left)**, this bar chart displays the proportion of direct evidence (orange) contained in each network estimate; **Minimal Parallelism (center)**, this bar chart displays the minimum number of independent paths contributing to the effect estimate on an aggregated level; **Mean Path Length (right)**, this bar chart displays the mean path length, which characterizes the degree of indirectness of an estimate. Higher mean path lengths indicate less reliable estimates, given that more similarity assumptions have to be made when serially combining direct comparisons


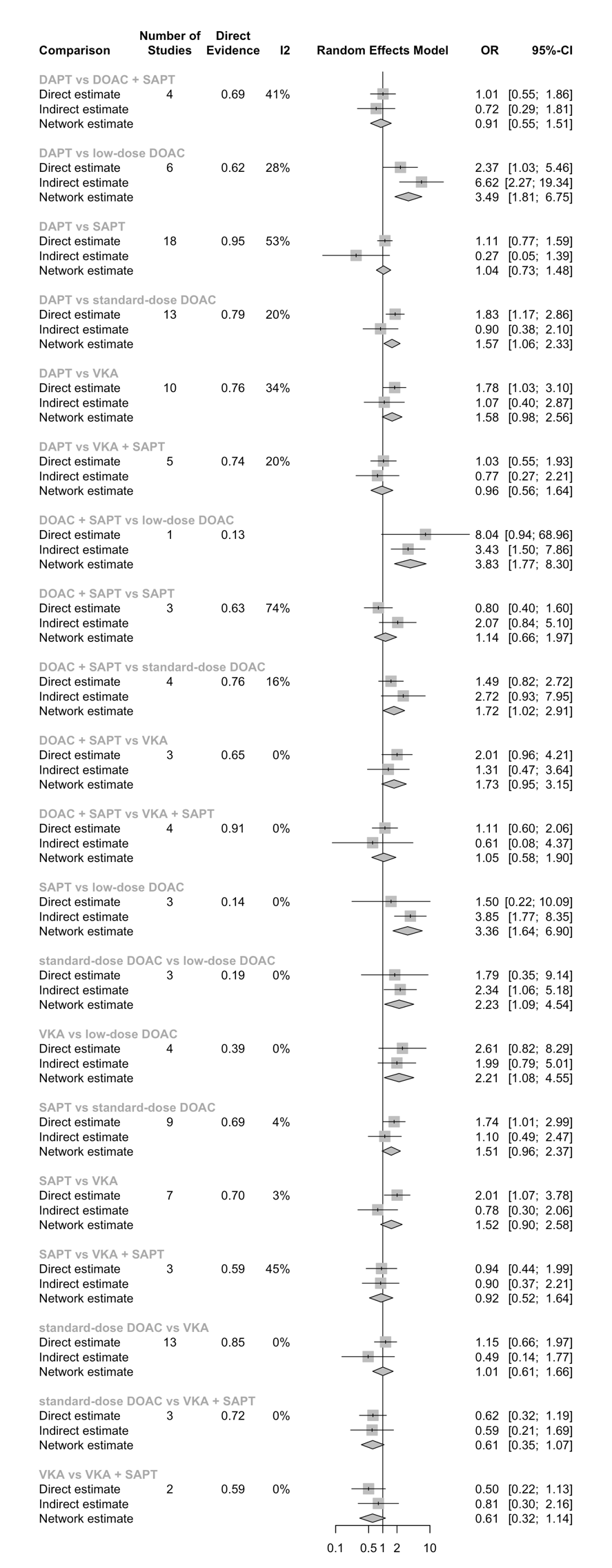


### **Supplemental Figure 14**. Split network estimates illustrating the contribution of direct and indirect evidence and local inconsistency in network meta-analysis for major bleeding.


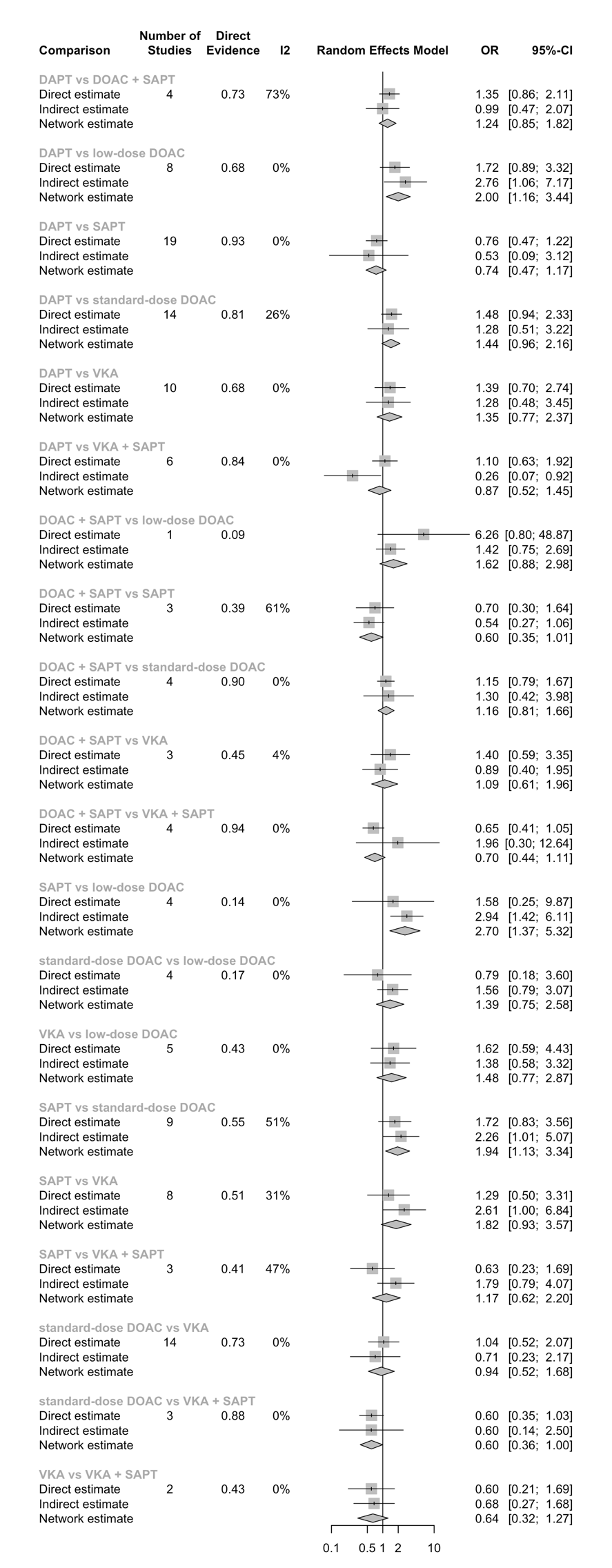


### **Supplemental Figure 15**. Split network estimates illustrating the contribution of direct and indirect evidence and local inconsistency in network meta-analysis for thromboembolic events.


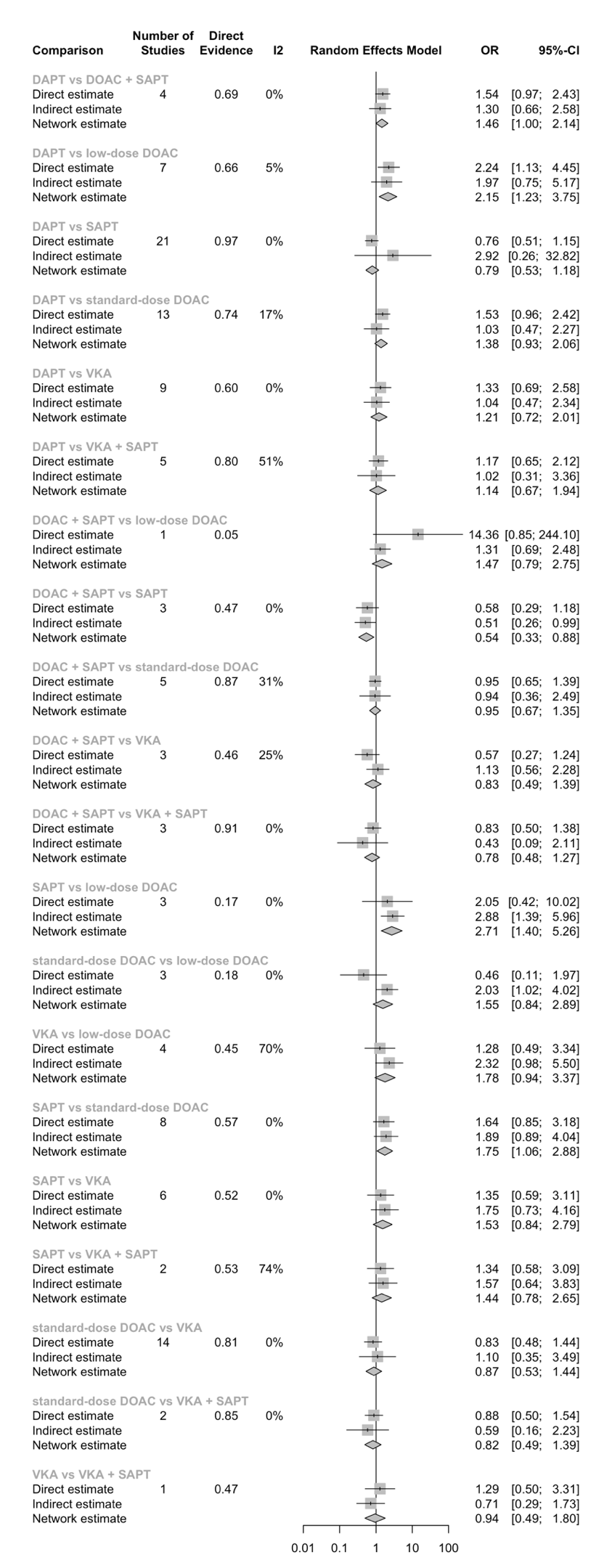


### **Supplemental Figure 16**. Split network estimates illustrating the contribution of direct and indirect evidence and local inconsistency in network meta-analysis for device-related thrombosis.


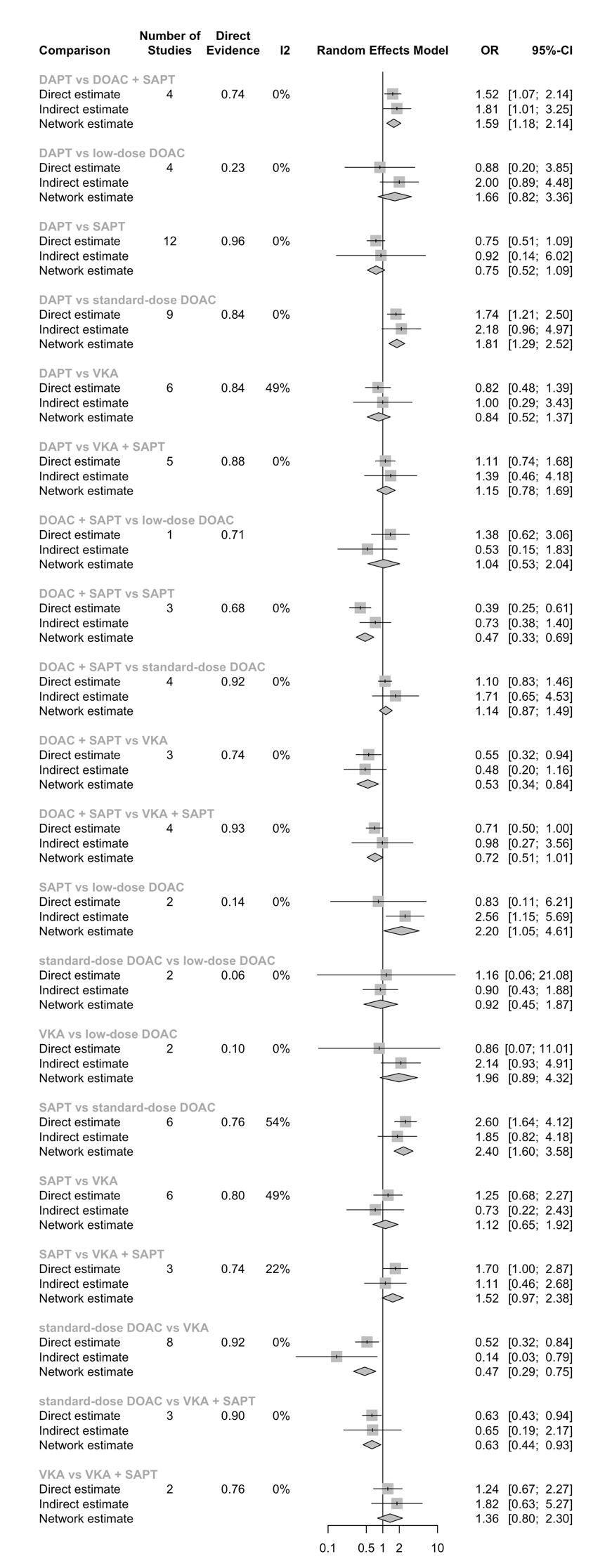


### **Supplemental Figure 17**. Split network estimates illustrating the contribution of direct and indirect evidence and local inconsistency in network meta-analysis for all-cause mortality.

### **Supplemental Table 8**. League table of the sensitivity analysis for major bleeding restricting follow-up to 3 months.

| DAPT | 0.94 (0.55 - 1.63) | 2.25 (0.93 - 5.45) | 1.29 (0.78 - 2.14) | 1.78 (1.08 - 2.93) | 1.45 (0.78 - 2.70) |
| --- | --- | --- | --- | --- | --- |
| 0.82 (0.51 - 1.33) | **DOAC plus SAPT** | 8.04 (0.98 - 66.26) | 1.15 (0.61 - 2.18) | 1.80 (1.04 - 3.13) | 2.13 (1.05 - 4.32) |
| 3.42 (1.72 - 6.79) | 4.15 (1.93 - 8.97) | **Low-dose DOAC** | 0.77 (0.06 - 9.33) | 0.52 (0.09 - 3.03) | 0.38 (0.12 - 1.17) |
| 1.18 (0.72 - 1.93) | 1.44 (0.81 - 2.53) | 0.35 (0.16 - 0.77) | **SAPT** | 1.65 (0.87 - 3.13) | 1.84 (0.90 - 3.80) |
| 1.56 (1.00 - 2.45) | 1.90 (1.15 - 3.15) | 0.46 (0.22 - 0.97) | 1.32 (0.76 - 2.31) | **Standard-dose DOAC** | 0.99 (0.54 - 1.82) |
| 1.38 (0.81 - 2.36) | 1.68 (0.93 - 3.04) | 0.40 (0.19 - 0.85) | 1.17 (0.62 - 2.19) | 0.88 (0.51 - 1.54) | **VKA** |
| 0.98 (0.60 - 1.62) | 1.20 (0.71 - 2.02) | 0.29 (0.13 - 0.64) | 0.83 (0.46 - 1.50) | 0.63 (0.37 - 1.07) | 0.71 (0.38 - 1.32) |

Comparisons should be read from left to right. Comparative estimates (reported as odds ratios with corresponding 95% confidence intervals) are located at the intersection between the treatment defined by the column and the treatment defined by the row. Bottom left rectangle: network estimates (synthesis of direct and indirect evidence). Top right rectangle: direct evidence (pairwise meta-analysis).


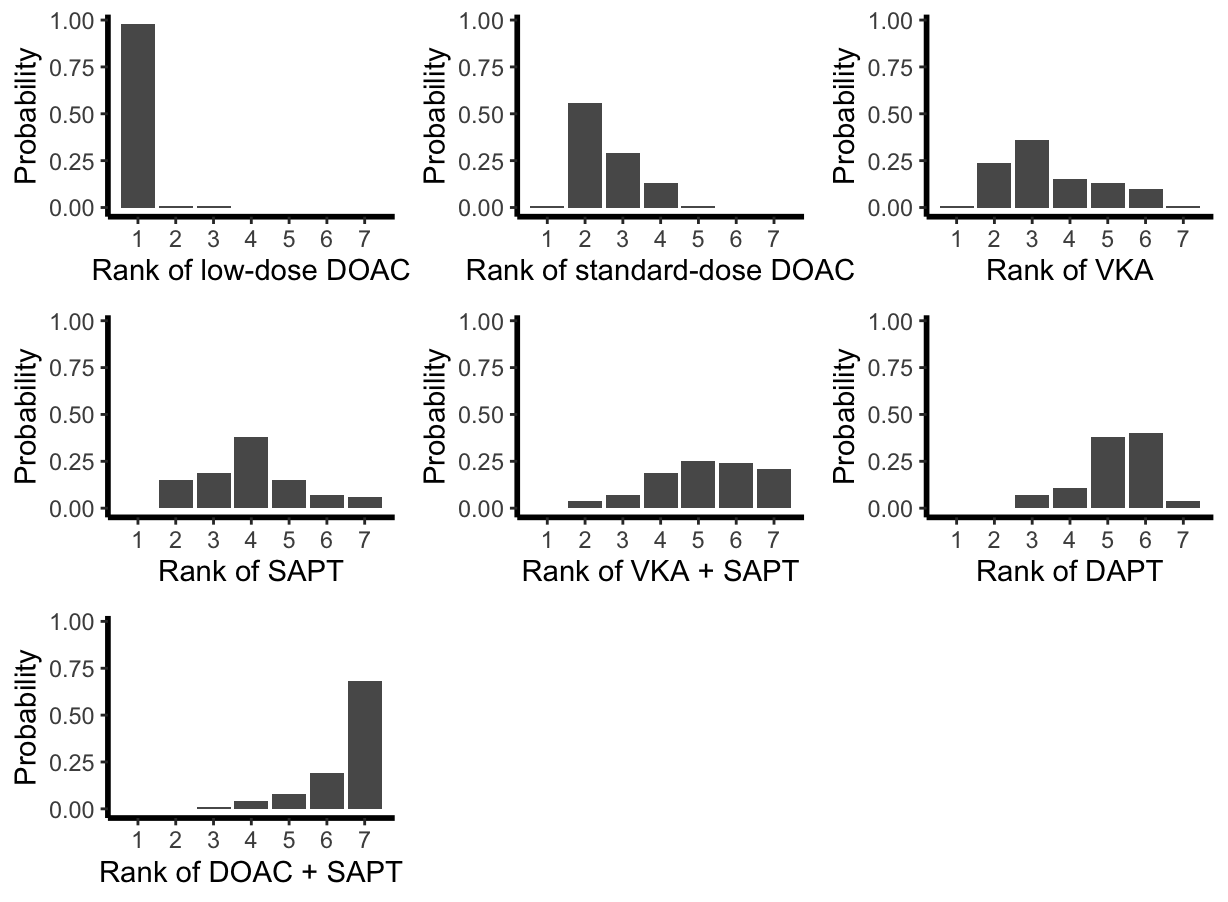


### **Supplemental Figure 18**. Ranking probabilities (rankograms) of the sensitivity analysis for major bleeding restricting follow-up to 3 months.

### **Supplemental Table 9**. League table of the sensitivity analysis for major bleeding restricting follow-up to 6 months.

| DAPT | 0.97 (0.59 - 1.59) | 2.23 (0.99 - 5.02) | 1.27 (0.80 - 2.02) | 1.75 (1.10 - 2.77) | 1.49 (0.82 - 2.71) |
| --- | --- | --- | --- | --- | --- |
| 0.84 (0.54 - 1.30) | **DOAC plus SAPT** | 8.04 (0.99 - 65.32) | 1.16 (0.64 - 2.10) | 1.81 (1.10 - 3.00) | 2.13 (1.09 - 4.17) |
| 3.32 (1.74 - 6.33) | 3.97 (1.92 - 8.19) | **Low-dose DOAC** | 0.67 (0.10 - 4.41) | 0.52 (0.09 - 2.99) | 0.38 (0.12 - 1.15) |
| 1.18 (0.75 - 1.86) | 1.42 (0.84 - 2.39) | 0.36 (0.17 - 0.75) | **SAPT** | 1.62 (0.90 - 2.94) | 1.84 (0.92 - 3.68) |
| 1.56 (1.03 - 2.37) | 1.87 (1.17 - 2.97) | 0.47 (0.23 - 0.96) | 1.32 (0.79 - 2.21) | **Standard-dose DOAC** | 1.00 (0.55 - 1.79) |
| 1.39 (0.83 - 2.34) | 1.67 (0.95 - 2.94) | 0.42 (0.20 - 0.86) | 1.18 (0.65 - 2.15) | 0.89 (0.52 - 1.53) | **VKA** |
| 1.00 (0.63 - 1.59) | 1.19 (0.74 - 1.94) | 0.30 (0.14 - 0.64) | 0.84 (0.49 - 1.46) | 0.64 (0.39 - 1.04) | 0.72 (0.40 - 1.29) |

Comparisons should be read from left to right. Comparative estimates (reported as odds ratios with corresponding 95% confidence intervals) are located at the intersection between the treatment defined by the column and the treatment defined by the row. Bottom left rectangle: network estimates (synthesis of direct and indirect evidence). Top right rectangle: direct evidence (pairwise meta-analysis).


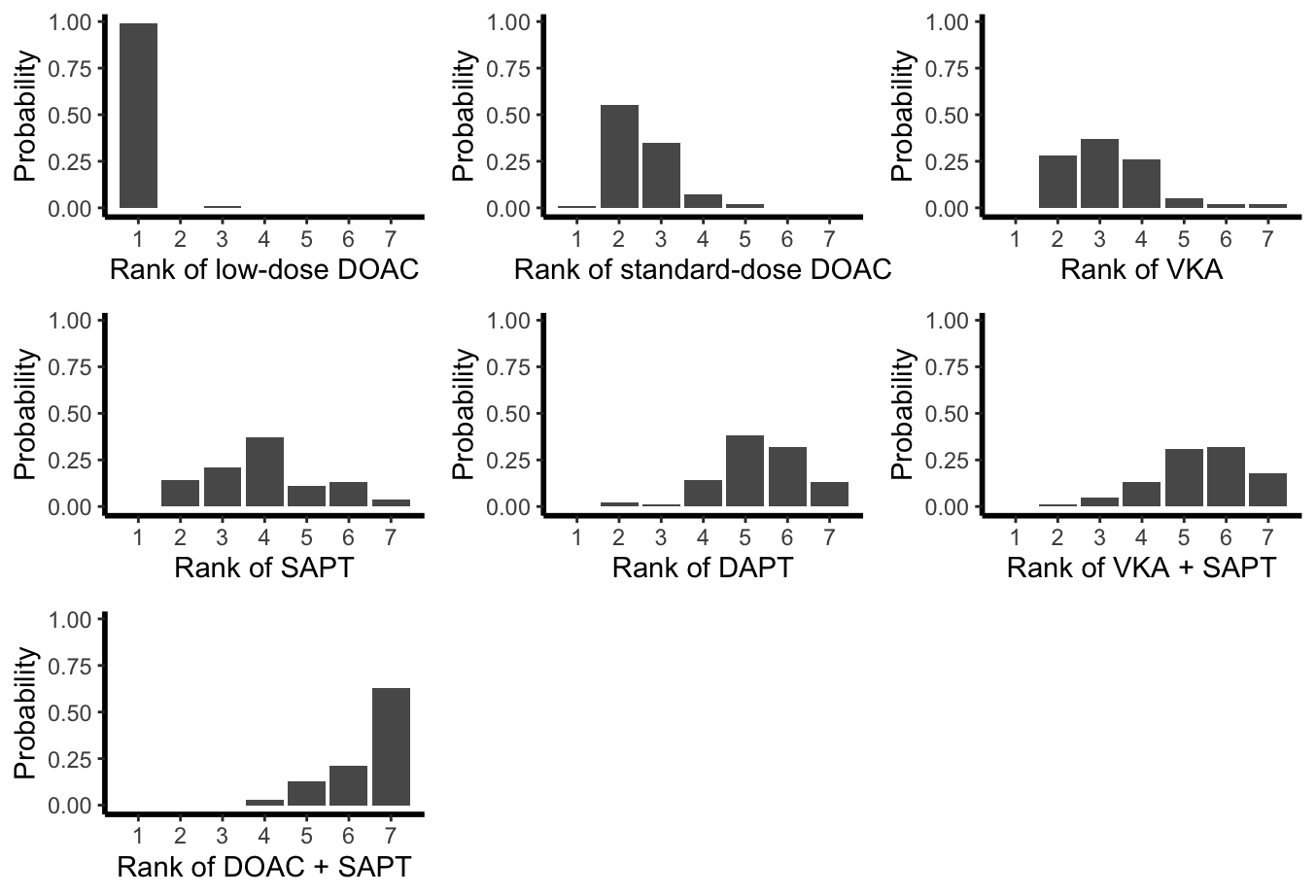


### **Supplemental Figure 19**. Ranking probabilities (rankograms) of the sensitivity analysis for major bleeding restricting follow-up to 6 months.

### **Supplemental Table 10**. League table of the sensitivity analysis for device-related thrombosis restricting follow-up to 3 months.

| DAPT | 1.45 (0.76 - 2.76) | 2.31 (1.03 - 5.17) | 0.72 (0.41 - 1.25) | 1.43 (0.78 - 2.62) | 1.26 (0.59 - 2.73) |
| --- | --- | --- | --- | --- | --- |
| 1.41 (0.82 - 2.42) | **DOAC plus SAPT** | 14.36 (0.81 - 254.65) | 0.59 (0.26 - 1.37) | 0.82 (0.46 - 1.44) | 0.55 (0.23 - 1.32) |
| 2.05 (1.08 - 3.90) | 1.46 (0.68 - 3.12) | **Low-dose DOAC** | 0.92 (0.11 - 7.68) | 2.62 (0.51 - 13.33) | 0.80 (0.30 - 2.14) |
| 0.73 (0.43 - 1.26) | 0.52 (0.27 - 1.00) | 0.36 (0.16 - 0.79) | **SAPT** | 1.89 (0.86 - 4.18) | 1.67 (0.65 - 4.31) |
| 1.23 (0.74 - 2.04) | 0.88 (0.52 - 1.48) | 0.60 (0.29 - 1.23) | 1.68 (0.89 - 3.16) | **Standard-dose DOAC** | 0.84 (0.46 - 1.55) |
| 1.14 (0.63 - 2.04) | 0.81 (0.43 - 1.53) | 0.55 (0.27 - 1.12) | 1.55 (0.76 - 3.15) | 0.92 (0.53 - 1.61) | **VKA** |
| 0.98 (0.52 - 1.86) | 0.70 (0.37 - 1.31) | 0.48 (0.21 - 1.11) | 1.34 (0.64 - 2.81) | 0.79 (0.42 - 1.50) | 0.86 (0.41 - 1.81) |

Comparisons should be read from left to right. Comparative estimates (reported as odds ratios with corresponding 95% confidence intervals) are located at the intersection between the treatment defined by the column and the treatment defined by the row. Bottom left rectangle: network estimates (synthesis of direct and indirect evidence). Top right rectangle: direct evidence (pairwise meta-analysis).


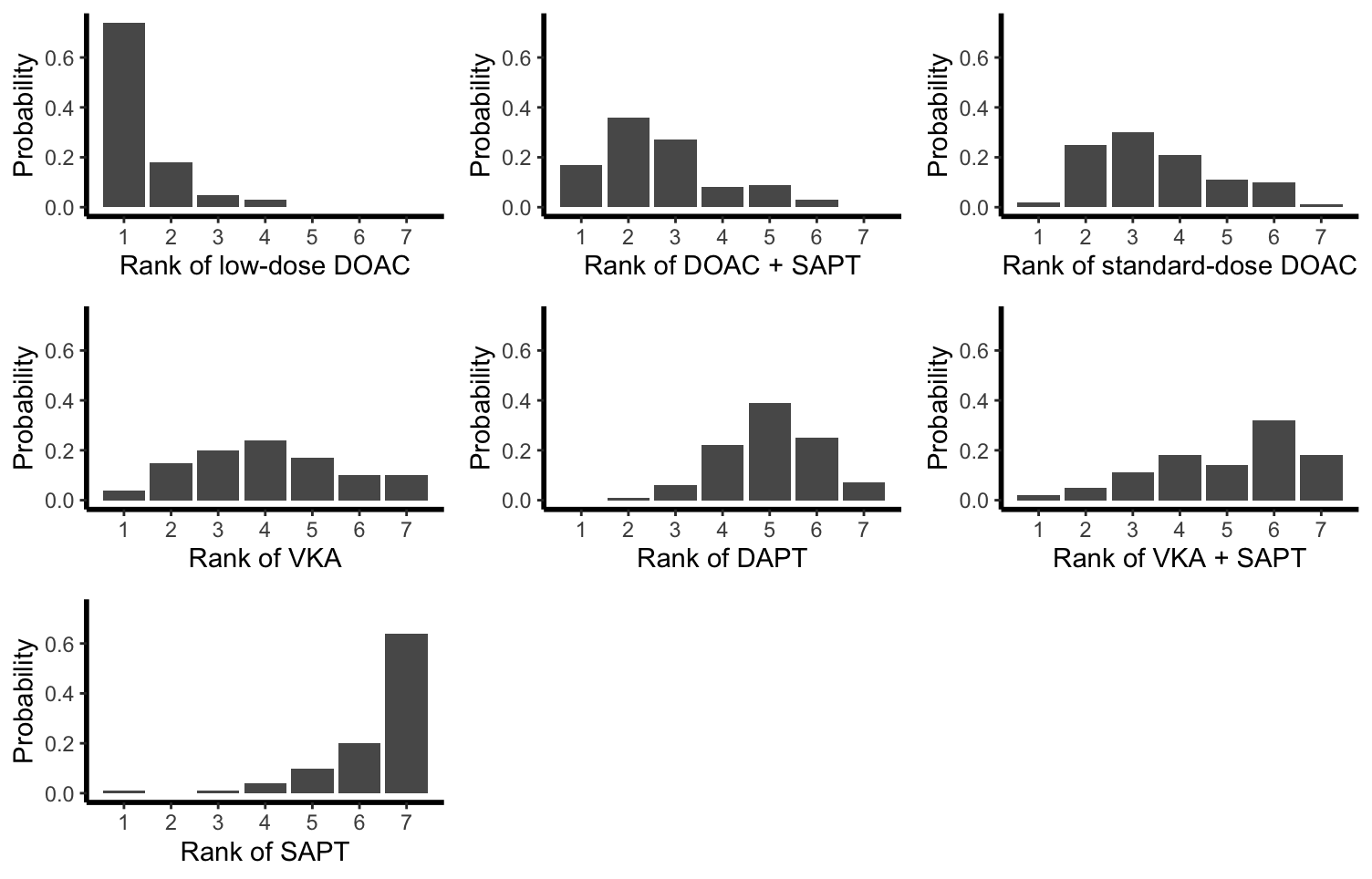


### **Supplemental Figure 20**. Ranking probabilities (rankograms) of the sensitivity analysis for device-related thrombosis restricting follow-up to 3 months.

### **Supplemental Table 11**. League table of the sensitivity analysis for device-related thrombosis restricting follow-up to 6 months.

| DAPT | 1.50 (0.88 - 2.56) | 2.15 (1.04 - 4.42) | 0.68 (0.42 - 1.10) | 1.42 (0.84 - 2.41) | 1.26 (0.61 - 2.60) |
| --- | --- | --- | --- | --- | --- |
| 1.41 (0.90 - 2.20) | **DOAC plus SAPT** | 14.36 (0.83 - 247.60) | 0.58 (0.28 - 1.24) | 0.90 (0.57 - 1.42) | 0.57 (0.25 - 1.27) |
| 2.05 (1.14 - 3.69) | 1.46 (0.74 - 2.86) | **Low-dose DOAC** | 0.48 (0.10 - 2.39) | 2.61 (0.53 - 12.87) | 0.79 (0.30 - 2.07) |
| 0.71 (0.44 - 1.14) | 0.50 (0.29 - 0.88) | 0.34 (0.17 - 0.70) | **SAPT** | 1.87 (0.91 - 3.83) | 1.64 (0.66 - 4.06) |
| 1.28 (0.82 - 2.00) | 0.91 (0.59 - 1.39) | 0.62 (0.32 - 1.20) | 1.81 (1.03 - 3.17) | **Standard-dose DOAC** | 0.83 (0.46 - 1.49) |
| 1.15 (0.66 - 1.99) | 0.81 (0.46 - 1.45) | 0.56 (0.29 - 1.09) | 1.62 (0.84 - 3.14) | 0.90 (0.53 - 1.52) | **VKA** |
| 1.07 (0.61 - 1.90) | 0.76 (0.44 - 1.31) | 0.52 (0.24 - 1.13) | 1.52 (0.78 - 2.96) | 0.84 (0.48 - 1.48) | 0.94 (0.47 - 1.86) |

Comparisons should be read from left to right. Comparative estimates (reported as odds ratios with corresponding 95% confidence intervals) are located at the intersection between the treatment defined by the column and the treatment defined by the row. Bottom left rectangle: network estimates (synthesis of direct and indirect evidence). Top right rectangle: direct evidence (pairwise meta-analysis).


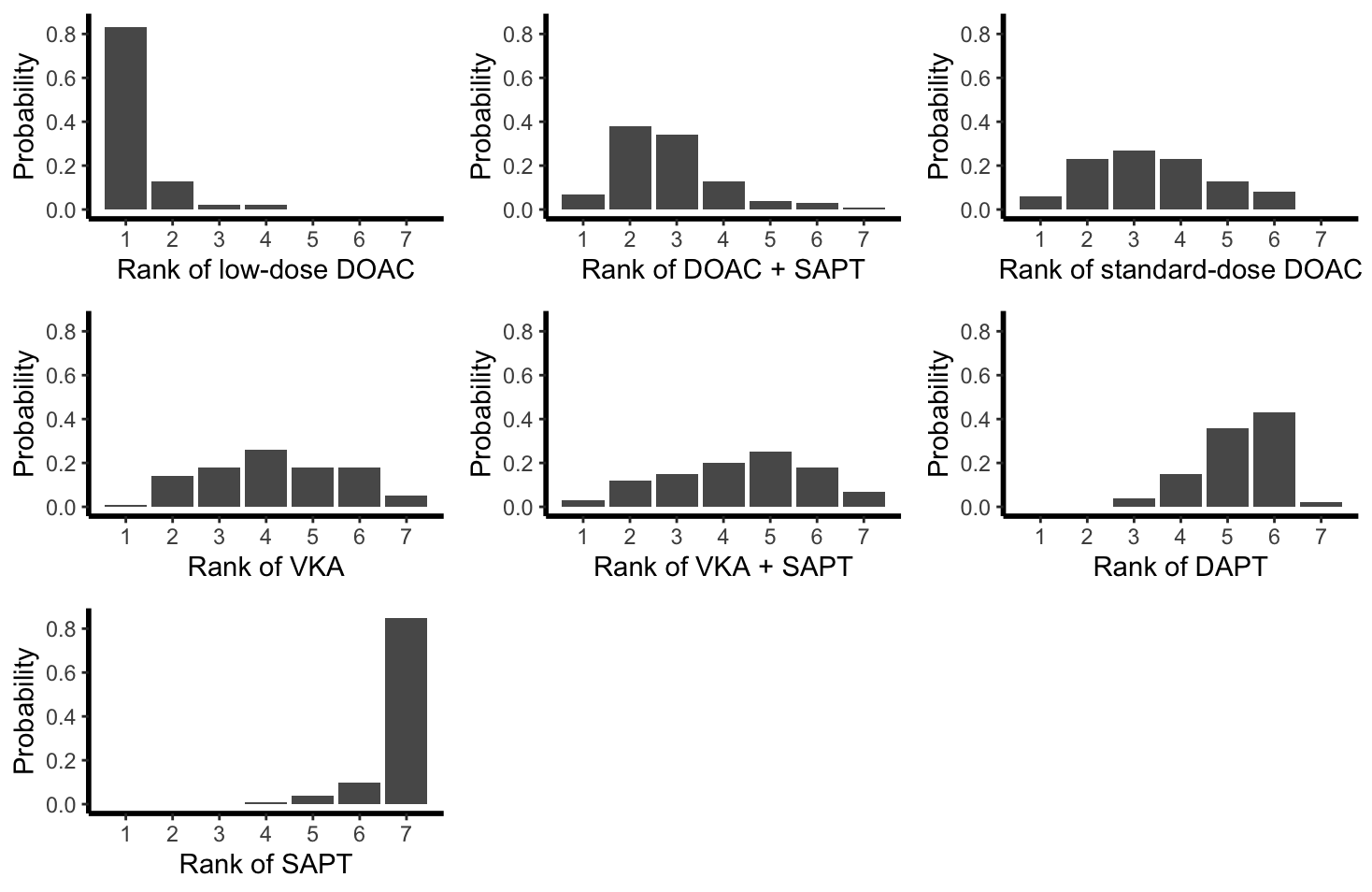


### **Supplemental Figure 21**. Ranking probabilities (rankograms) of the sensitivity analysis for device-related thrombosis restricting follow-up to 6 months.

### **Supplemental Table 12**. League table presenting results of sensitivity analyses limited to observational studies, evaluating the relative odds of major bleeding across treatment comparisons.

| DAPT | 1.00 (0.54 - 1.88) | 1.53 (0.35 - 6.60) | 1.10 (0.76 - 1.59) | 1.83 (1.16 - 2.89) | 1.80 (1.02 - 3.16) | 1.03 (0.54 - 1.94) |
| --- | --- | --- | --- | --- | --- | --- |
| 0.92 (0.55 - 1.56) | DOAC + SAPT | 8.04 (0.93 - 69.45) | 0.79 (0.39 - 1.60) | 1.48 (0.80 - 2.74) | 2.00 (0.94 - 4.26) | 1.11 (0.59 - 2.08) |
| 3.95 (1.66 - 9.42) | 4.29 (1.70 - 10.81) | low-dose DOAC | 0.67 (0.10 - 4.53) | 0.56 (0.11 - 2.86) | 0.38 (0.12 - 1.23) | . |
| 1.04 (0.73 - 1.49) | 1.13 (0.65 - 1.97) | 0.26 (0.11 - 0.65) | SAPT | 1.74 (1.01 - 3.01) | 2.02 (1.07 - 3.81) | 0.94 (0.44 - 2.01) |
| 1.58 (1.05 - 2.38) | 1.72 (1.00 - 2.94) | 0.40 (0.17 - 0.97) | 1.52 (0.96 - 2.40) | standard-dose DOAC | 1.15 (0.66 - 2.00) | 0.62 (0.31 - 1.21) |
| 1.61 (0.98 - 2.66) | 1.75 (0.95 - 3.22) | 0.41 (0.17 - 0.97) | 1.55 (0.90 - 2.66) | 1.02 (0.61 - 1.70) | VKA | 0.50 (0.22 - 1.15) |
| 0.96 (0.56 - 1.66) | 1.04 (0.57 - 1.91) | 0.24 (0.09 - 0.64) | 0.92 (0.51 - 1.66) | 0.61 (0.34 - 1.07) | 0.60 (0.31 - 1.14) | VKA + SAPT |

### **Supplemental Figure 22**. Forest plot from random-effects network meta-analysis evaluating the risk of major bleeding, based on sensitivity analysis restricted to observational studies.


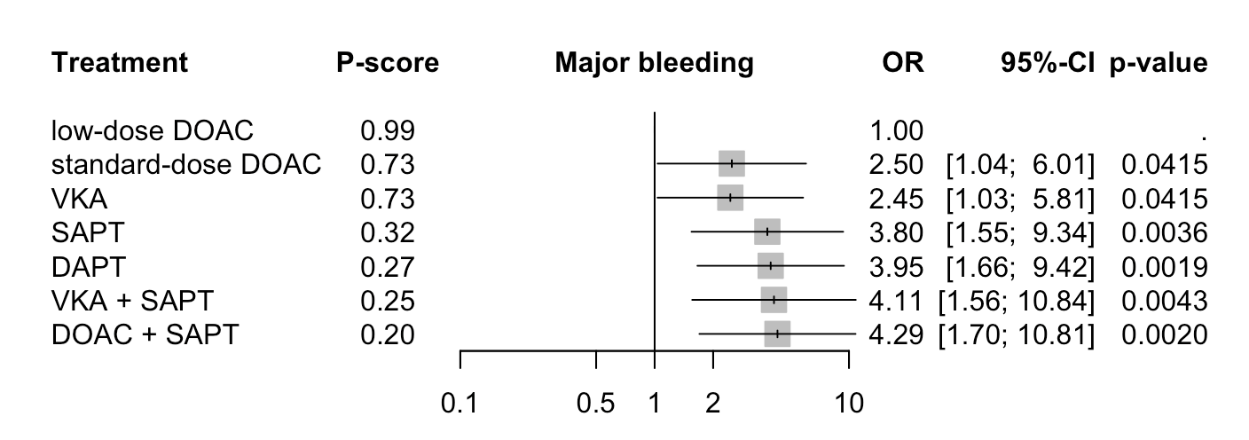


### **Supplemental Table 13**. League table presenting results of sensitivity analyses limited to observational studies, evaluating the relative odds of thromboembolism across treatment comparisons.

| DAPT | 1.33 (0.84 - 2.11) | 1.63 (0.81 - 3.27) | 0.76 (0.47 - 1.22) | 1.48 (0.93 - 2.36) | 1.40 (0.70 - 2.80) | 1.10 (0.62 - 1.94) |
| --- | --- | --- | --- | --- | --- | --- |
| 1.23 (0.83 - 1.82) | DOAC + SAPT | 6.26 (0.80 - 49.04) | 0.70 (0.30 - 1.64) | 1.15 (0.78 - 1.71) | 1.40 (0.58 - 3.37) | 0.65 (0.40 - 1.06) |
| 1.96 (1.11 - 3.44) | 1.60 (0.85 - 3.00) | low-dose DOAC | 0.63 (0.10 - 3.94) | 1.26 (0.28 - 5.72) | 0.62 (0.22 - 1.69) | . |
| 0.74 (0.47 - 1.17) | 0.60 (0.35 - 1.03) | 0.38 (0.19 - 0.76) | SAPT | 1.72 (0.83 - 3.57) | 1.29 (0.50 - 3.32) | 0.63 (0.23 - 1.70) |
| 1.44 (0.95 - 2.18) | 1.17 (0.81 - 1.70) | 0.74 (0.39 - 1.39) | 1.94 (1.13 - 3.35) | standard-dose DOAC | 1.04 (0.52 - 2.10) | 0.60 (0.35 - 1.04) |
| 1.34 (0.76 - 2.38) | 1.10 (0.60 - 1.99) | 0.69 (0.35 - 1.35) | 1.82 (0.92 - 3.59) | 0.94 (0.52 - 1.70) | VKA | 0.60 (0.21 - 1.70) |
| 0.86 (0.51 - 1.45) | 0.70 (0.44 - 1.13) | 0.44 (0.21 - 0.91) | 1.17 (0.62 - 2.20) | 0.60 (0.36 - 1.00) | 0.64 (0.32 - 1.29) | VKA + SAPT |

### **Supplemental Figure 23**. Forest plot from random-effects network meta-analysis evaluating the risk of thromboembolism, based on sensitivity analysis restricted to observational studies.


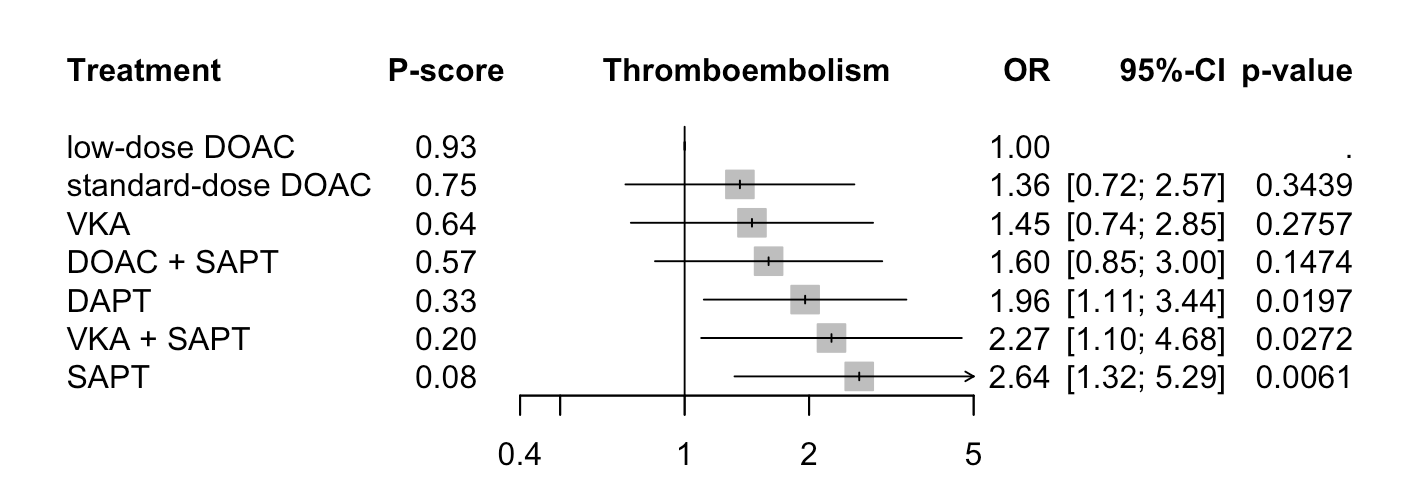


### **Supplemental Table 14**. League table presenting results of sensitivity analyses limited to observational studies, evaluating the relative odds of device-related thrombosis across treatment comparisons.

| DAPT | 1.49 (0.86 - 2.60) | 1.85 (0.86 - 3.96) | 0.76 (0.50 - 1.16) | 1.50 (0.88 - 2.53) | 1.37 (0.67 - 2.78) | 1.12 (0.58 - 2.16) |
| --- | --- | --- | --- | --- | --- | --- |
| 1.44 (0.91 - 2.29) | DOAC + SAPT | 14.36 (0.83 - 248.90) | 0.59 (0.27 - 1.27) | 0.88 (0.54 - 1.42) | 0.56 (0.25 - 1.28) | 0.82 (0.45 - 1.47) |
| 1.87 (1.02 - 3.43) | 1.30 (0.65 - 2.60) | low-dose DOAC | 0.48 (0.10 - 2.40) | 2.20 (0.50 - 9.69) | 0.79 (0.30 - 2.08) | . |
| 0.78 (0.51 - 1.19) | 0.54 (0.31 - 0.93) | 0.42 (0.21 - 0.84) | SAPT | 1.61 (0.80 - 3.25) | 1.35 (0.57 - 3.20) | 1.31 (0.53 - 3.20) |
| 1.30 (0.84 - 2.03) | 0.90 (0.58 - 1.41) | 0.70 (0.36 - 1.36) | 1.67 (0.98 - 2.85) | standard-dose DOAC | 0.83 (0.46 - 1.49) | 0.89 (0.47 - 1.69) |
| 1.15 (0.67 - 1.98) | 0.80 (0.45 - 1.43) | 0.62 (0.31 - 1.21) | 1.48 (0.79 - 2.76) | 0.88 (0.52 - 1.50) | VKA | 1.29 (0.47 - 3.51) |
| 1.09 (0.61 - 1.95) | 0.76 (0.43 - 1.33) | 0.58 (0.27 - 1.28) | 1.40 (0.73 - 2.68) | 0.84 (0.47 - 1.49) | 0.95 (0.48 - 1.89) | VKA + SAPT |

### **Supplemental Figure 24**. Forest plot from random-effects network meta-analysis evaluating the risk of device-related thrombosis, based on sensitivity analysis restricted to observational studies.


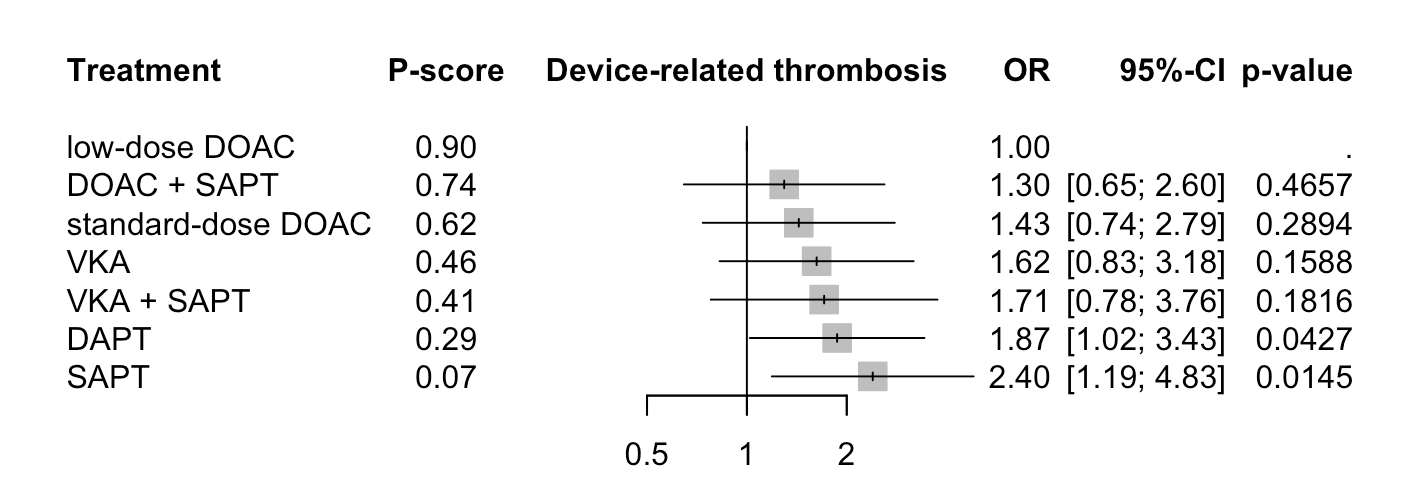


### **Supplemental Table 15**. League table presenting results of sensitivity analyses limited to observational studies, evaluating the relative odds of all-cause mortality across treatment comparisons.

| DAPT | 1.52 (1.07 - 2.14) | 0.94 (0.18 - 4.90) | 0.75 (0.51 - 1.09) | 1.74 (1.21 - 2.50) | 0.82 (0.48 - 1.39) | 1.11 (0.74 - 1.68) |
| --- | --- | --- | --- | --- | --- | --- |
| 1.60 (1.19 - 2.15) | DOAC + SAPT | 1.38 (0.62 - 3.06) | 0.39 (0.25 - 0.61) | 1.10 (0.83 - 1.46) | 0.55 (0.32 - 0.94) | 0.71 (0.50 - 1.00) |
| 1.73 (0.84 - 3.57) | 1.08 (0.55 - 2.15) | low-dose DOAC | 1.20 (0.16 - 8.97) | 0.87 (0.05 - 15.77) | 1.16 (0.09 - 14.88) | . |
| 0.76 (0.52 - 1.09) | 0.47 (0.33 - 0.69) | 0.44 (0.21 - 0.93) | SAPT | 2.60 (1.64 - 4.12) | 1.25 (0.68 - 2.27) | 1.70 (1.00 - 2.87) |
| 1.81 (1.30 - 2.53) | 1.14 (0.87 - 1.49) | 1.05 (0.51 - 2.17) | 2.40 (1.61 - 3.58) | standard-dose DOAC | 0.52 (0.32 - 0.84) | 0.63 (0.43 - 0.94) |
| 0.85 (0.52 - 1.38) | 0.53 (0.34 - 0.84) | 0.49 (0.22 - 1.09) | 1.12 (0.65 - 1.92) | 0.47 (0.29 - 0.75) | VKA | 1.24 (0.67 - 2.27) |
| 1.15 (0.78 - 1.69) | 0.72 (0.51 - 1.01) | 0.67 (0.31 - 1.41) | 1.52 (0.97 - 2.39) | 0.63 (0.43 - 0.93) | 1.36 (0.80 - 2.30) | VKA + SAPT |

### **Supplemental Figure 25**. Forest plot from random-effects network meta-analysis evaluating the risk of all-cause mortality, based on sensitivity analysis restricted to observational studies.


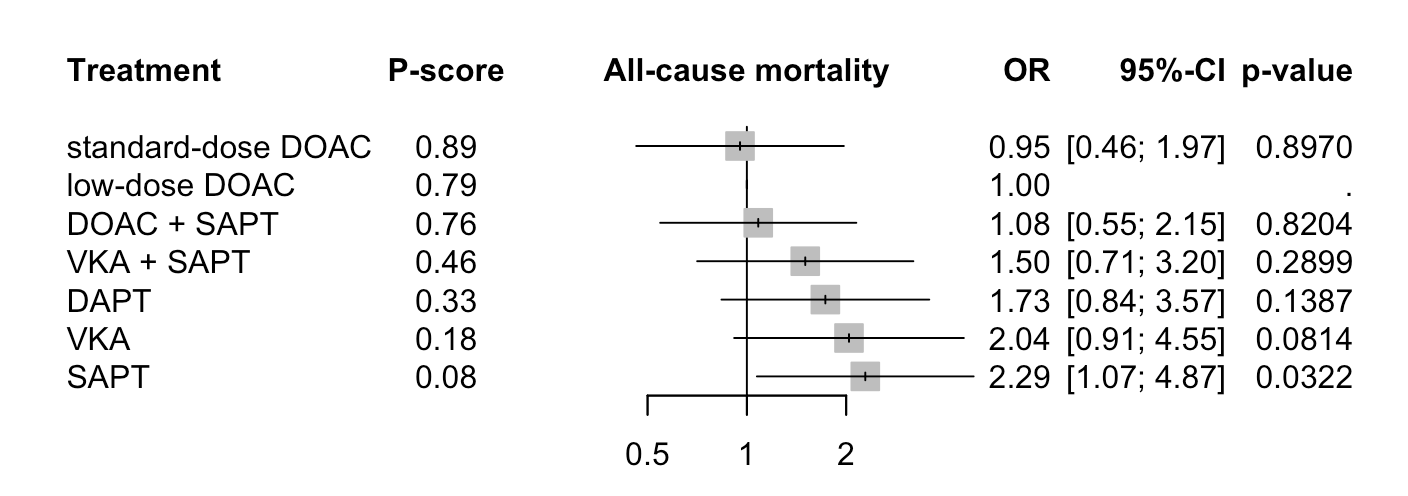


### **Supplemental Table 16**. Certainty of evidence ratings for major bleeding derived using the Confidence in Network Meta-Analysis (CINeMA) framework.

| **Comparison** | **Within-study bias** | **Across-studies bias** | **Indirectness** | **Imprecision** | **Heterogeneity** | **Incoherence** | **Overall confidence** |
| --- | --- | --- | --- | --- | --- | --- | --- |
| Low-dose DOAC vs Standard-dose DOAC | Major concerns | Some concerns | Some concerns | Major concerns | Some concerns | No concerns | Low |
| Standard-dose DOAC vs SAPT | Major concerns | Major concerns | Some concerns | Some concerns | Some concerns | No concerns | Low |
| Standard-dose DOAC vs DAPT | Major concerns | Major concerns | Some concerns | Some concerns | Major concerns | Some concerns | Very Low |
| Low-dose DOAC vs SAPT | Major concerns | Major concerns | Some concerns | Major concerns | Some concerns | No concerns | Very Low |
| Low-dose DOAC vs DAPT | Major concerns | Major concerns | Some concerns | Major concerns | Some concerns | No concerns | Low |
| Any DOAC vs VKA+SAPT | Major concerns | Some concerns | Some concerns | Some concerns | Some concerns | Some concerns | Low |
| Standard-dose DOAC vs VKA | Major concerns | Some concerns | Some concerns | Some concerns | Some concerns | No concerns | Low |
| DAPT vs SAPT | Major concerns | Some concerns | Major concerns | Some concerns | Major concerns | Some concerns | Very Low |

### **Supplemental Table 17**. Certainty of evidence ratings for thromboembolism derived using the Confidence in Network Meta-Analysis (CINeMA) framework.

| Comparison | Within-study bias | Across-studies bias | Indirectness | Imprecision | Heterogeneity | Incoherence | Overall confidence |
| --- | --- | --- | --- | --- | --- | --- | --- |
| Low-dose DOAC vs Standard-dose DOAC | Major concerns | Some concerns | Some concerns | Major concerns | Some concerns | No concerns | Low |
| Standard-dose DOAC vs SAPT | Major concerns | Major concerns | Some concerns | Some concerns | Some concerns | No concerns | Low |
| Standard-dose DOAC vs DAPT | Major concerns | Major concerns | Some concerns | Some concerns | Major concerns | Some concerns | Very Low |
| Low-dose DOAC vs SAPT | Major concerns | Major concerns | Some concerns | Major concerns | Some concerns | No concerns | Very Low |
| Low-dose DOAC vs DAPT | Major concerns | Major concerns | Some concerns | Major concerns | Some concerns | No concerns | Low |
| Any DOAC vs VKA+SAPT | Major concerns | Some concerns | Some concerns | Some concerns | Some concerns | Some concerns | Low |
| Standard-dose DOAC vs VKA | Major concerns | Some concerns | Some concerns | Some concerns | Some concerns | No concerns | Low |
| DAPT vs SAPT | Major concerns | Some concerns | Major concerns | Some concerns | Major concerns | Some concerns | Very Low |

### **Supplemental Table 18**. Certainty of evidence ratings for device-related thrombosis derived using the Confidence in Network Meta-Analysis (CINeMA) framework.

| Comparison | Within-study bias | Across-studies bias | Indirectness | Imprecision | Heterogeneity | Incoherence | Overall confidence |
| --- | --- | --- | --- | --- | --- | --- | --- |
| Low-dose DOAC vs Standard-dose DOAC | Major concerns | Some concerns | Some concerns | Major concerns | Some concerns | No concerns | Low |
| Standard-dose DOAC vs SAPT | Major concerns | Major concerns | Some concerns | Some concerns | Some concerns | No concerns | Low |
| Standard-dose DOAC vs DAPT | Major concerns | Major concerns | Some concerns | Some concerns | Major concerns | Some concerns | Very Low |
| Low-dose DOAC vs SAPT | Major concerns | Major concerns | Some concerns | Major concerns | Some concerns | No concerns | Very Low |
| Low-dose DOAC vs DAPT | Major concerns | Major concerns | Some concerns | Major concerns | Some concerns | No concerns | Low |
| Any DOAC vs VKA+SAPT | Major concerns | Some concerns | Some concerns | Some concerns | Some concerns | Some concerns | Low |
| Standard-dose DOAC vs VKA | Major concerns | Some concerns | Some concerns | Some concerns | Some concerns | No concerns | Low |
| DAPT vs SAPT | Major concerns | Some concerns | Major concerns | Some concerns | Major concerns | Some concerns | Very Low |

### **Supplemental Table 19**. Certainty of evidence ratings for all-cause mortality derived using the Confidence in Network Meta-Analysis (CINeMA) framework.

| Comparison | Within-study bias | Across-studies bias | Indirectness | Imprecision | Heterogeneity | Incoherence | Overall confidence |
| --- | --- | --- | --- | --- | --- | --- | --- |
| Low-dose DOAC vs Standard-dose DOAC | Major concerns | Some concerns | Some concerns | Major concerns | Some concerns | No concerns | Low |
| Standard-dose DOAC vs SAPT | Major concerns | Major concerns | Some concerns | Some concerns | Some concerns | No concerns | Low |
| Standard-dose DOAC vs DAPT | Major concerns | Major concerns | Some concerns | Some concerns | Major concerns | Some concerns | Very Low |
| Low-dose DOAC vs SAPT | Major concerns | Major concerns | Some concerns | Major concerns | Some concerns | No concerns | Very Low |
| Low-dose DOAC vs DAPT | Major concerns | Major concerns | Some concerns | Major concerns | Some concerns | No concerns | Low |
| Any DOAC vs VKA+SAPT | Major concerns | Some concerns | Some concerns | Some concerns | Some concerns | Some concerns | Low |
| Standard-dose DOAC vs VKA | Major concerns | Some concerns | Some concerns | Some concerns | Some concerns | No concerns | Low |
| DAPT vs SAPT | Major concerns | Some concerns | Major concerns | Some concerns | Major concerns | Some concerns | Very Low |


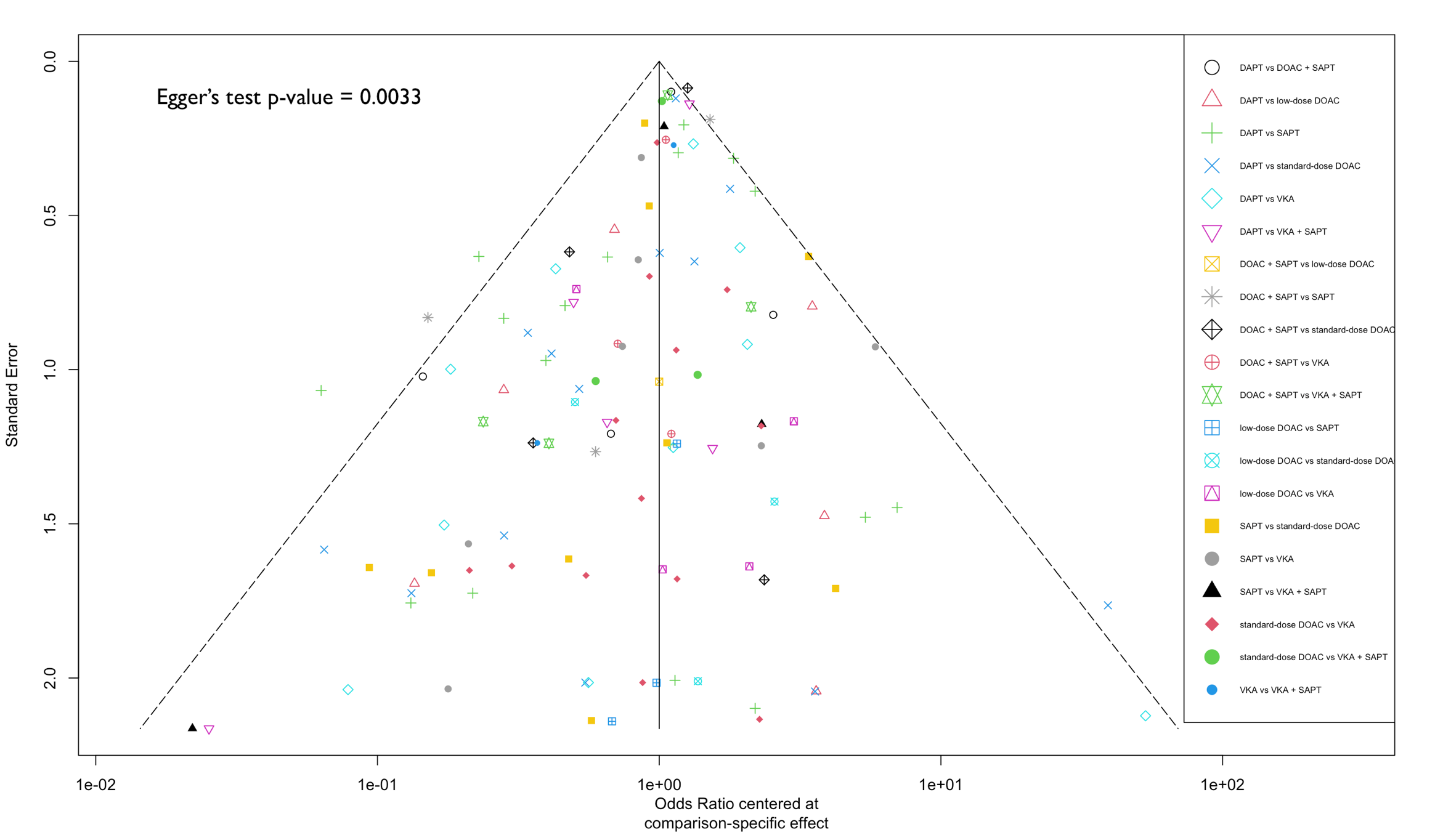


### **Supplemental Figure 26**. Funnel plot of network meta-analysis depicting the relationship between effect size versus standard error for the effect of different antithrombotic strategies on major bleeding events.


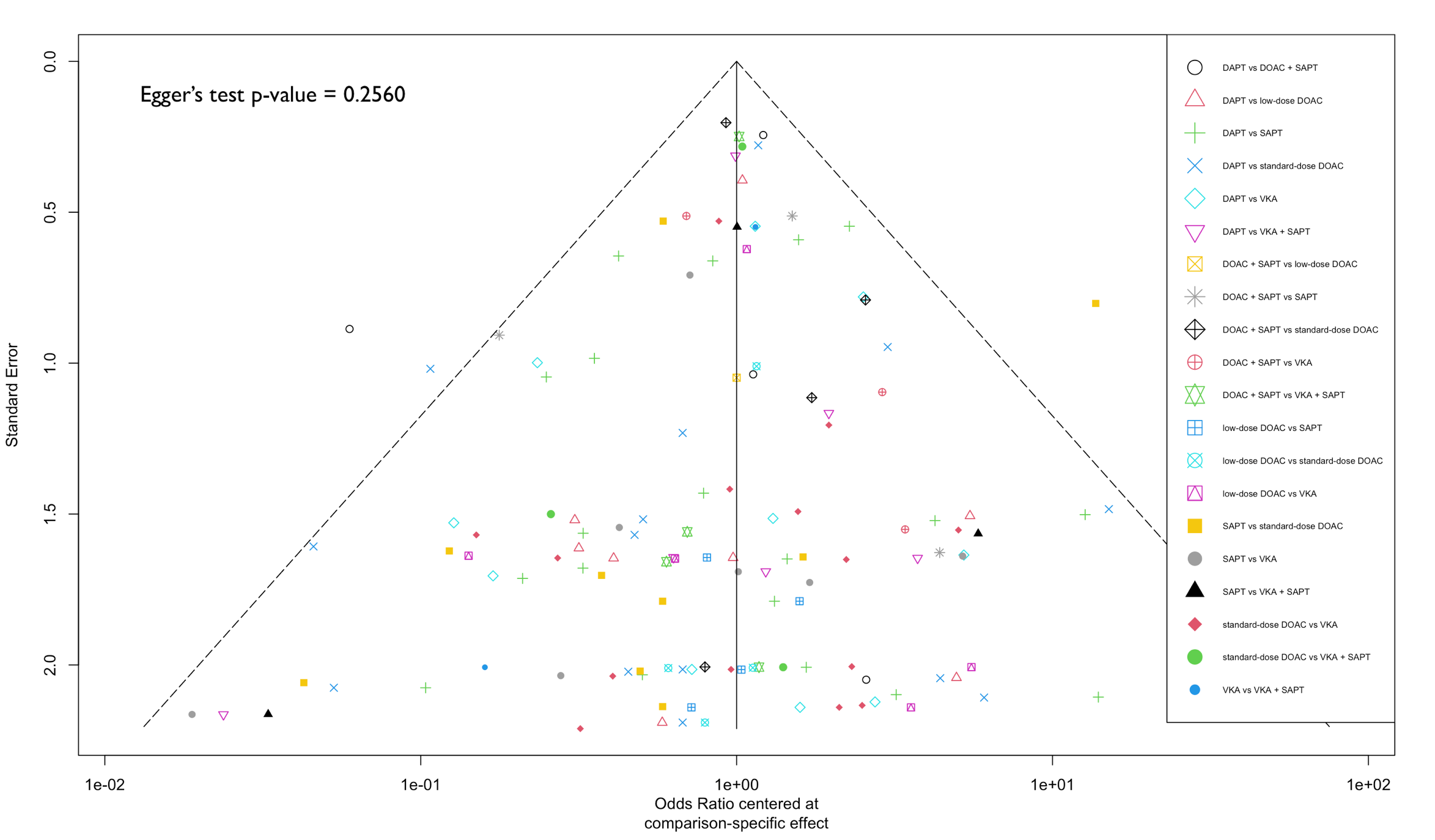


### **Supplemental Figure 27**. Funnel plot of network meta-analysis depicting the relationship between effect size versus standard error for the effect of different antithrombotic strategies on thromboembolic events.


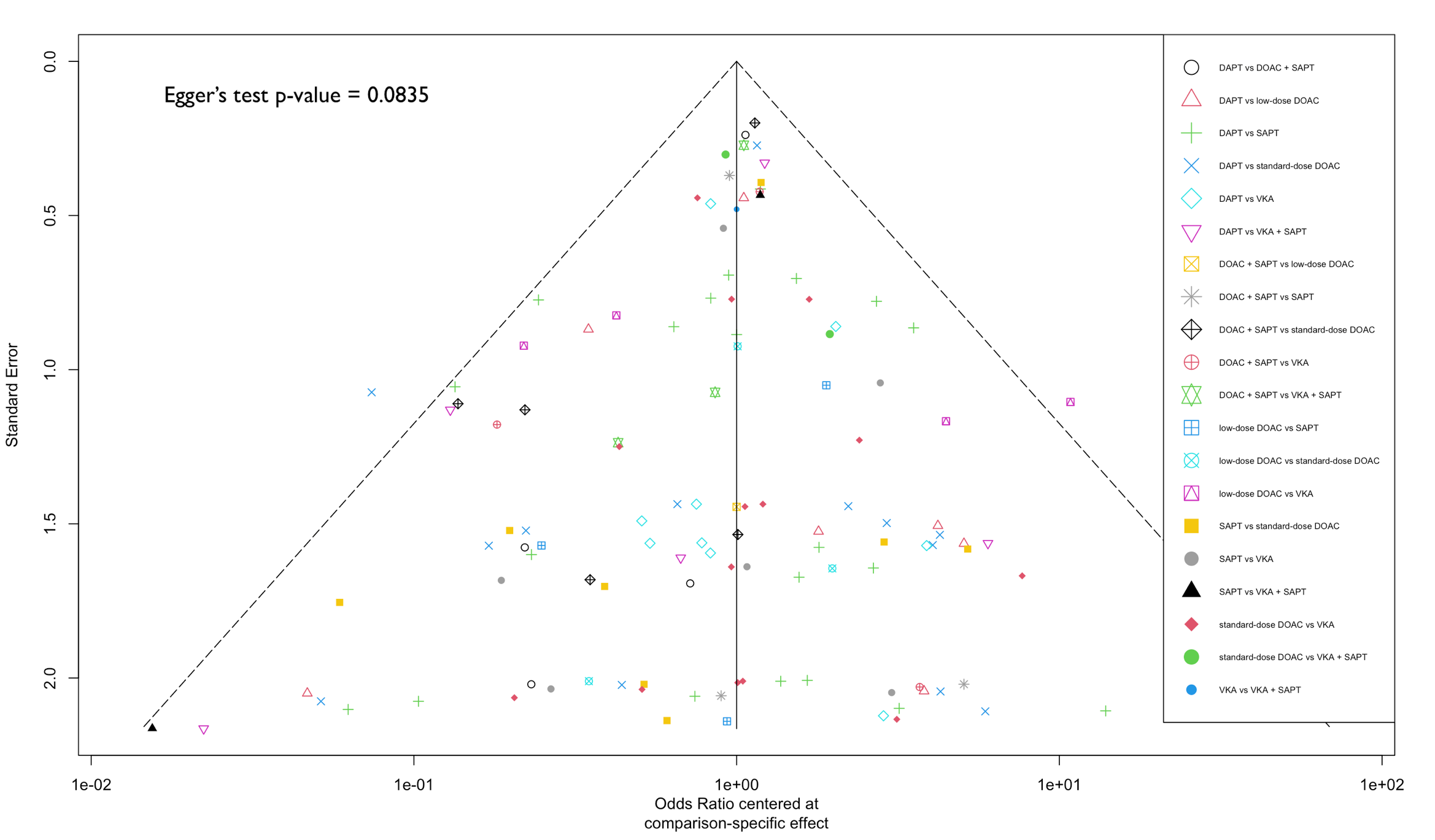


### **Supplemental Figure 28**. Funnel plot of network meta-analysis depicting the relationship between effect size versus standard error for the effect of different antithrombotic strategies on device-related thrombosis.


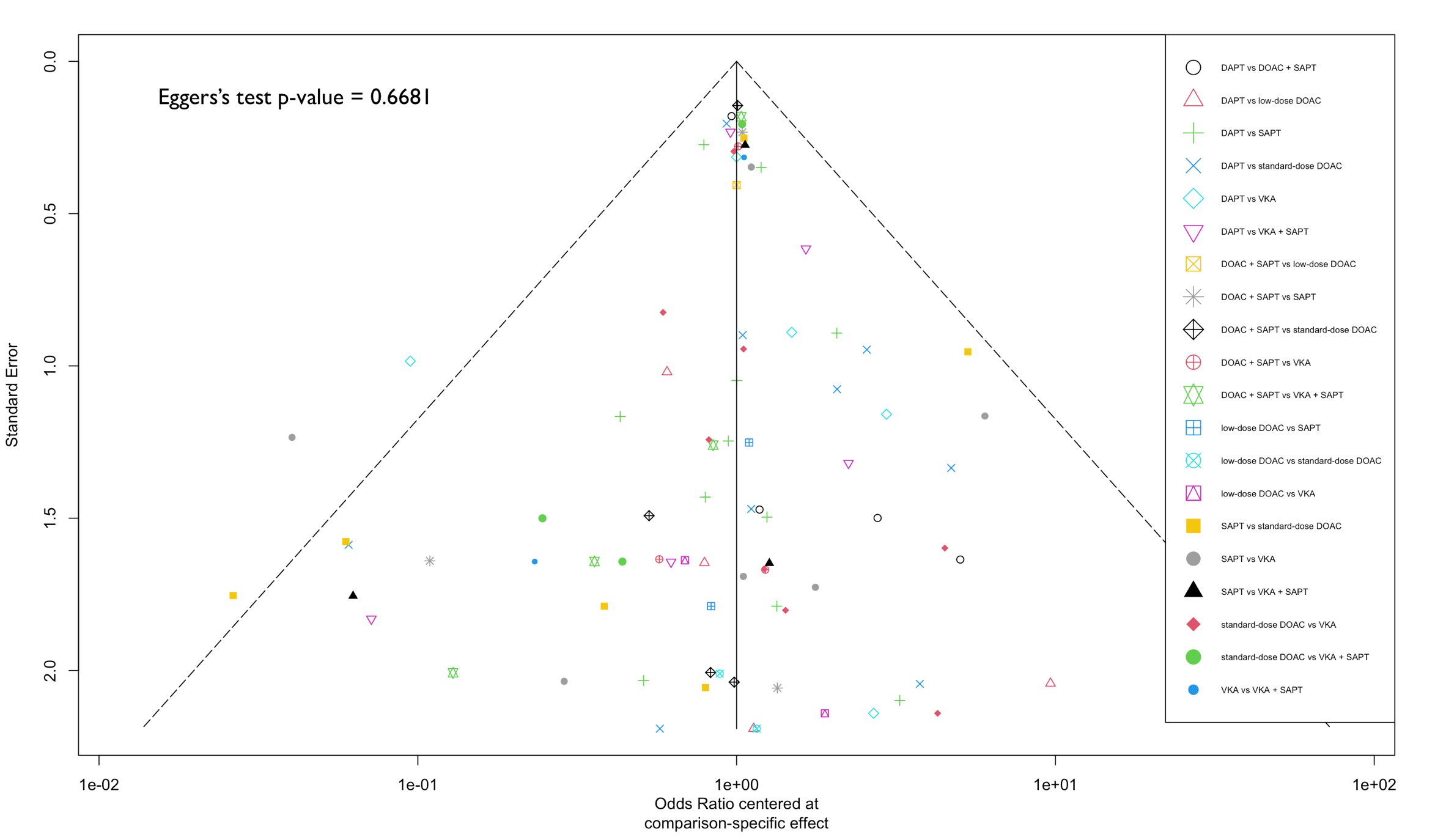


### **Supplemental Figure 29**. Funnel plot of network meta-analysis depicting the relationship between effect size versus standard error for the effect of different antithrombotic strategies on all-cause mortality.


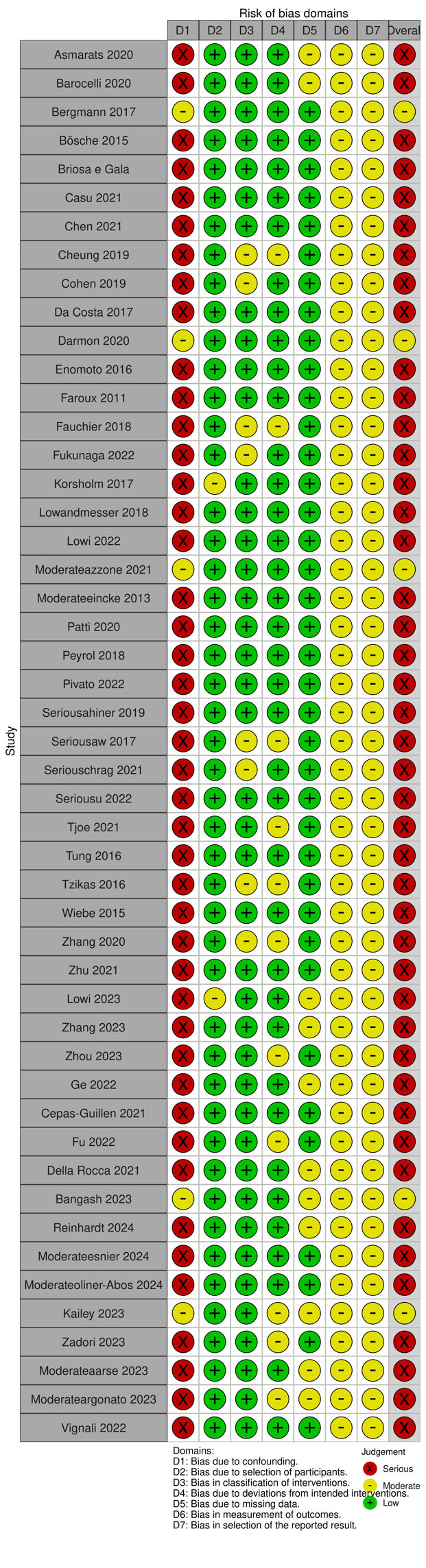


### **Supplemental Figure 30.** Risk of Bias in Non-randomized Studies of Interventions (ROBINS-I)


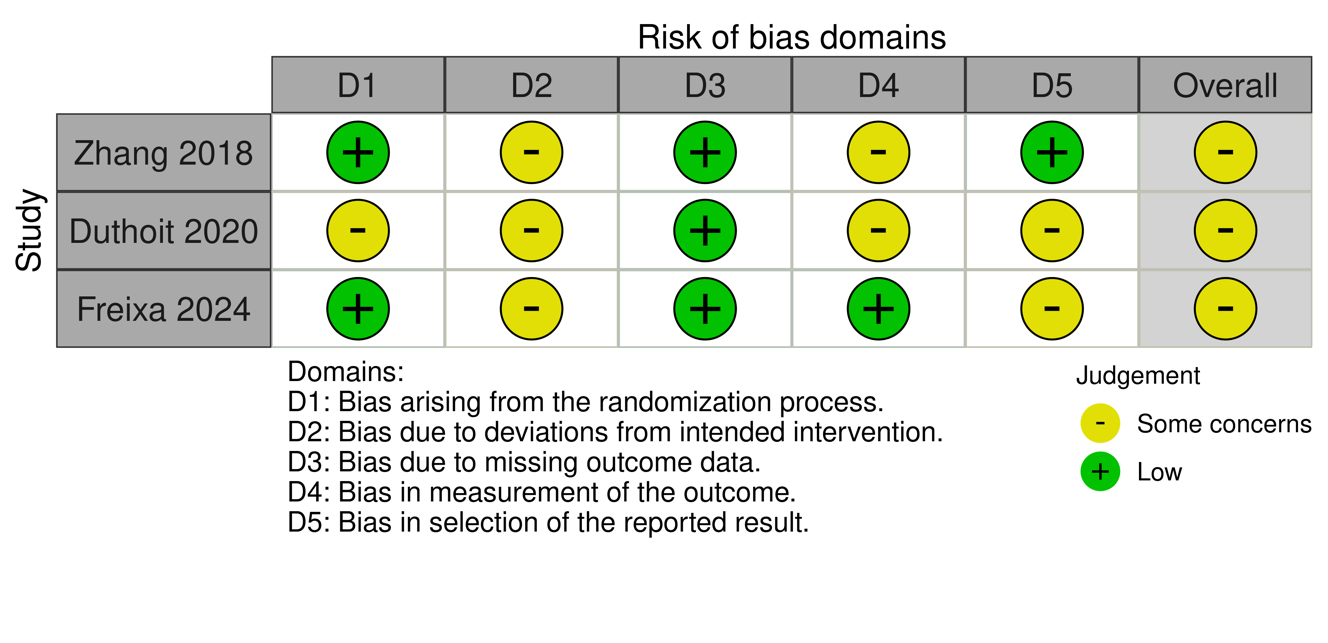


### **Supplemental Figure 31.** Risk of Bias in randomized studies (Revised Cochrane risk of bias tool for randomized trials RoB2)

**References**

1. Freixa X, Cruz-González I, Cepas-Guillén P, Millán X, Antúnez-Muiños P, Flores-Umanzor E, et al. Low-Dose Direct Oral Anticoagulation vs Dual Antiplatelet Therapy After Left  Atrial Appendage Occlusion: The ADALA Randomized Clinical Trial. JAMA Cardiol. 2024 Oct;9(10):922–6.

2. Reinhardt SW, Gibson DN, Hsu JC, Kapadia SR, Yeh RW, Price MJ, et al. Anticoagulation Alone vs Anticoagulation Plus Aspirin or DAPT Following  Left Atrial Appendage Occlusion. J Am Coll Cardiol. 2024 Sep;84(10):889–900.

3. Mesnier J, Cruz-González I, Guedeney P, Arzamendi D, Freixa X, Nombela-Franco L, et al. Early Nonprocedural Bleeding After Left Atrial Appendage Occlusion. JACC Cardiovascular interventions. 2024 Aug;17(15):1765–76.

4. Moliner-Abós C, Albertí-Vall B, Millan-Álvarez X, Camps-Renom P, Guasch-Jiménez M, Asmarats-Serra L, et al. Left atrial appendage occlusion in patients with spontaneous intracerebral  hemorrhage: An observational study. Journal of stroke and cerebrovascular diseases : the official journal of National  Stroke Association. 2024 Feb;33(2):107481.

5. Li X, Jin Q, Yao Y, Zhang X, Lv Q. Clinical Effectiveness and Safety Comparison between Reduced Rivaroxaban Dose and  Dual Antiplatelet Therapy for Nonvalvular Atrial Fibrillation Patients Following Percutaneous Left Atrial Appendage Closure: A Prospective Observational Study. Reviews in cardiovascular medicine. 2023 Nov;24(11):335.

6. Zhang X, Xing Z, Fang C, Yang M, Luo J, Ning Z. Safety and Efficacy of Cryoballoon Pulmonary Vein Isolation and Left Atrial  Appendage Closure Combined Procedure and Half-Dose Rivaroxaban After Operation in Elderly Patients with Atrial Fibrillation. Journal of multidisciplinary healthcare. 2023;16:2603–11.

7. Zhou XD, Chen QF, Lin F, Wang L, Chen Y, Liang D, et al. Reduced- or Half-Dose Rivaroxaban Following Left Atrial Appendage Closure: A  Feasible Antithrombotic Therapy in Patients at High Risk of Bleeding? Journal of clinical medicine. 2023 Jan;12(3).

8. Bangash AB, Li Y, Huang W, Zhong J, Zheng H, Zhang D, et al. Left atrial appendage occlusion using the LAmbre device in atrial fibrillation  patients with a history of ischemic stroke: 1-Year outcomes from a multicenter study in China. Pacing and clinical electrophysiology : PACE. 2023 Dec;46(12):1478–83.

9. Kailey BS, Koa-Wing M, Sutaria N, Mott T, Sohaib A, Qureshi N, et al. Left atrial appendage occlusion for atrial fibrillation and bleeding diathesis. Journal of cardiovascular electrophysiology. 2023 Dec;34(12):2552–62.

10. Zadori A, Kis Z, Toth T, Szigeti M, Temesvari A, Fontos G, et al. Long-Term Efficacy and Safety of Left Atrial Appendage Closure Procedures. International heart journal. 2023 Mar;64(2):188–95.

11. Maarse M, Aarnink EW, Huijboom MFM, Abeln BGS, Staal D, Rensing BJWM, et al. Long-term outcomes of successful left atrial appendage occlusion with focus on  stroke prevention: 10-year follow-up of a single-center registry. Heart rhythm O2. 2023 May;4(5):298–308.

12. Margonato D, Preda A, Ingallina G, Rizza V, Fierro N, Radinovic A, et al. Left atrial appendage occlusion after thromboembolic events or left atrial  appendage sludge during anticoagulation therapy: Is two better than one? Real-world experience from a tertiary care hospital. Journal of arrhythmia. 2023 Jun;39(3):395–404.

13. Ge H, Zhang C, Qiao ZQ, Hao ZY, Li Z, Gu ZC, et al. Risk of device-related thrombosis following short-term oral anticoagulation with  low-dose dabigatran versus warfarin after Watchman left atrial appendage occlusion. Science progress. 2022;105(3):368504221113194.

14. Fu G, Wang B, He B, Yu Y, Wang Z, Feng M, et al. Safety and efficacy of low-dose non-vitamin K antagonist oral anticoagulants  versus warfarin after left atrial appendage closure with the Watchman device. Journal of the Formosan Medical Association = Taiwan yi zhi. 2022 Aug;121(8):1488–94.

15. Vignali L, Gurgoglione FL, Barocelli F, Cattabiani MA, Solinas E, Maini A, et al. Looking for optimal antithrombotic strategy after transcatheter left atrial  appendage occlusion: a real-world comparison of different antiplatelet regimens. International journal of cardiology. 2023 Jan;371:92–9.

16. Fukunaga M, Isotani A, Shirai S, Murakami N, Nakamura M, Morinaga T, et al. Initial practice of left atrial appendage closure device in Japan; single-center  experience. Journal of cardiology. 2022 Jun;79(6):752–8.

17. Li W, Gao R, Zhao J, Ren Y, Chen G, Zhu J, et al. Safety and efficacy of different anticoagulation regimens after left atrial  appendage occlusion. Annals of palliative medicine. 2022 Jan;11(1):201–9.

18. Pivato CA, Liccardo G, Sanz-Sanchez J, Pelloni E, Pujdak K, Xuareb RG, et al. Left atrial appendage closure with the II generation Ultraseal device: An  international registry. The LIGATE study. Catheterization and cardiovascular interventions : official journal of the  Society for Cardiac Angiography & Interventions. 2022 Oct;100(4):620–7.

19. Su F, Gao C, Liu J, Ning Z, He B, Liu Y, et al. Periprocedural Outcomes Associated With Use of a Left Atrial Appendage Occlusion  Device in China. JAMA network open. 2022 May;5(5):e2214594.

20. Cepas-Guillen PL, Flores-Umanzor E, Regueiro A, Brugaletta S, Ibañez C, Sanchis L, et al. Low Dose of Direct Oral Anticoagulants after Left Atrial Appendage Occlusion. J Cardiovasc Dev Dis. 2021 Oct;8(11).

21. Della Rocca DG, Magnocavallo M, Di Biase L, Mohanty S, Trivedi C, Tarantino N, et al. Half-Dose Direct Oral Anticoagulation Versus Standard Antithrombotic Therapy  After Left Atrial Appendage Occlusion. JACC Cardiovascular interventions. 2021 Nov;14(21):2353–64.

22. Schrag M, Mac Grory B, Nackenoff A, Eaton J, Mistry E, Kirshner H, et al. Left Atrial Appendage Closure for Patients with Cerebral Amyloid Angiopathy and  Atrial Fibrillation: the LAA-CAA Cohort. Translational stroke research. 2021 Apr;12(2):259–65.

23. Briosa E Gala A, Pope MTB, Monteiro C, Leo M, Dawkins S, Newton JD, et al. Long-term outcomes and periprocedural safety and efficacy of percutaneous left  atrial appendage closure in a United Kingdom tertiary center: An 11-year experience. Heart rhythm. 2021 Oct;18(10):1724–32.

24. Casu G, D’Angelo G, Ugo F, Ronco F, Simonetto F, Barbierato M, et al. Left atrial appendage occlusion in atrial fibrillation patients with previous  intracranial bleeding: A national multicenter study. International journal of cardiology. 2021 Apr;328:75–80.

25. Chen Y, Zhang Y, Qu L, Huang W, Su X, Chen Y. Short-term non-vitamin K antagonist oral anticoagulants vs. warfarin in  preventing device-related thrombosis after left atrial appendage closure. Journal of thrombosis and thrombolysis. 2021 Oct;52(3):872–9.

26. Faroux L, Cruz-González I, Arzamendi D, Freixa X, Nombela-Franco L, Peral V, et al. Short-term direct oral anticoagulation or dual antiplatelet therapy following  left atrial appendage closure in patients with relative contraindications to chronic anticoagulation therapy. International journal of cardiology. 2021 Jun;333:77–82.

27. Mazzone P, Laricchia A, D’Angelo G, Falasconi G, Pannone L, Limite LR, et al. Role of Different Antithrombotic Regimens after Percutaneous Left Atrial  Appendage Occlusion: A Large Single Center Experience. Journal of clinical medicine. 2021 May;10(9).

28. Tjoe B, Nguyen H, Mandava S, Quintos J, Li J, Yim S, et al. Use of Direct Oral Anticoagulation Therapy Following Implantation of the Watchman Left Atrial Appendage Occlusion Device. Structural Heart. 2021;5(3):295–301.

29. Zhu J, Xu J. The Use of Novel Non-Vitamin K Antagonist Oral Anticoagulants Following Closure  of the Left Atrial Appendage: Preliminary Results of Clinical Follow-Up. Drug design, development and therapy. 2021;15:1067–73.

30. Duthoit G, Silvain J, Marijon E, Ducrocq G, Lepillier A, Frere C, et al. Reduced Rivaroxaban Dose Versus Dual Antiplatelet Therapy After Left Atrial  Appendage Closure: ADRIFT a Randomized Pilot Study. Circulation Cardiovascular interventions. 2020 Jul;13(7):e008481.

31. Asmarats L, O’Hara G, Champagne J, Paradis JM, Bernier M, O’Connor K, et al. Short-Term Oral Anticoagulation Versus Antiplatelet Therapy Following  Transcatheter Left Atrial Appendage Closure. Circulation Cardiovascular interventions. 2020 Aug;13(8):e009039.

32. Barocelli F, Coli S, Crocamo A, Guidorossi A, Cattabiani MA, Preti F, et al. Left atrial appendage occlusion in patients with atrial fibrillation and large  prevalence of prior intracranial bleeding. Journal of cardiovascular medicine (Hagerstown, Md). 2020 Aug;21(8):583–91.

33. Darmon A, Couture EL, Stein G, Cormier B, Chevalier B, Lefèvre T, et al. Left Atrial Appendage Closure in Patients With Atrial Fibrillation at Very High  Bleeding Risk Without Postimplantation Antithrombotic Therapy. The Journal of invasive cardiology. 2020 Oct;32(10):385–91.

34. Patti G, Sticchi A, Verolino G, Pasceri V, Vizzi V, Brscic E, et al. Safety and Efficacy of Single Versus Dual Antiplatelet Therapy After Left Atrial  Appendage Occlusion. The American journal of cardiology. 2020 Nov;134:83–90.

35. Zhang ZH, Yao Q, Huang HY, Zhu P, Xu X, Song ZY, et al. “One-stop shop”: safety and efficacy of combining atrial septal defect occlusion  and left atrial appendage closure for patients with atrial septal defect and atrial fibrillation. BMC cardiovascular disorders. 2020 Oct;20(1):444.

36. Şahiner ML, Kaya EB, Çöteli C, Aytemir K. Left atrial appendage transcatheter occlusion with amplatzer^TM^ amulet^TM^ device: Real life data with mid-term follow-up results. Arquivos Brasileiros de Cardiologia. 2019 Oct 1;113(4):712–21.

37. Cheung GS, So KC, Chan CK, Chan AK, Lee APW, Lam YY, et al. Comparison of three left atrial appendage occlusion devices for stroke prevention  in patients with non-valvular atrial fibrillation: a single-centre seven-year experience with WATCHMAN, AMPLATZER Cardiac Plug/Amulet, LAmbre: Comparison of three LAAO devi. AsiaIntervention. 2019 Feb;5(1):57–63.

38. Cohen JA, Heist EK, Galvin J, Lee H, Johnson M, Fitzsimons M, et al. A comparison of postprocedural anticoagulation in high-risk patients undergoing  WATCHMAN device implantation. Pacing and clinical electrophysiology : PACE. 2019 Oct;42(10):1304–9.

39. Fauchier L, Cinaud A, Brigadeau F, Lepillier A, Pierre B, Abbey S, et al. Device-Related Thrombosis After Percutaneous Left Atrial Appendage Occlusion  for Atrial Fibrillation. J Am Coll Cardiol. 2018 Apr;71(14):1528–36.

40. Landmesser U, Tondo C, Camm J, Diener HC, Paul V, Schmidt B, et al. Left atrial appendage occlusion with the AMPLATZER Amulet device: One-year follow-up from the prospective global Amulet observational registry. EuroIntervention. 2018 Aug 3;14(5):e590–7.

41. Peyrol M, Cautela J, Salaun E, Miola C, Franceschi F, Thuny F, et al. Device-Related Thrombus After Left Atrial Appendage Occlusion With the Amulet  Device. Heart, lung & circulation. 2019 Nov;28(11):1683–8.

42. Zhang Z, Yao Q, Huang H, Wan C, Song Z, Li H kang. Efficacy and safety of dabigatran and dual antiplatelet therapy after left atrial appendage occlusion with the Watchman device. In 2018.

43. Bergmann MW, Betts TR, Sievert H, Schmidt B, Pokushalov E, Kische S, et al. Safety and efficacy of early anticoagulation drug regimens after WATCHMAN left  atrial appendage closure: three-month data from the EWOLUTION prospective, multicentre, monitored international WATCHMAN LAA closure registry. EuroIntervention. 2017 Sep;13(7):877–84.

44. Costa MJM da, Ferreira E, Quintella EF, Amorim B, Fuchs A, Zajdenverg R, et al. Left Atrial Appendage Closure with Amplatzer Cardiac Plug in Nonvalvular Atrial  Fibrillation: Safety and Long-Term Outcome. Arquivos brasileiros de cardiologia. 2017 Dec;109(6):541–9.

45. Korsholm K, Nielsen KM, Jensen JM, Jensen HK, Andersen G, Nielsen-Kudsk JE. Transcatheter left atrial appendage occlusion in patients with atrial  fibrillation and a high bleeding risk using aspirin alone for post-implant antithrombotic therapy. EuroIntervention : journal of EuroPCR in collaboration with the Working Group on  Interventional Cardiology of the European Society of Cardiology. 2017 Apr;12(17):2075–82.

46. Saw J, Tzikas A, Shakir S, Gafoor S, Omran H, Nielsen-Kudsk JE, et al. Incidence and Clinical Impact of Device-Associated Thrombus and Peri-Device Leak Following Left Atrial Appendage Closure With the Amplatzer Cardiac Plug. 2017.

47. Enomoto Y, Gadiyaram VK, Gianni C, Horton RP, Trivedi C, Mohanty S, et al. Use of non-warfarin oral anticoagulants instead of warfarin during left atrial  appendage closure with the Watchman device. Heart rhythm. 2017 Jan;14(1):19–24.

48. Tung MK, Ramkumar S, Cameron JD, Pang B, Nerlekar N, Kotschet E, et al. Retrospective Cohort Study Examining Reduced Intensity and Duration of Anticoagulant and Antiplatelet Therapy Following Left Atrial Appendage Occlusion with the WATCHMAN Device. Heart Lung and Circulation. 2017 May 1;26(5):477–85.

49. Tzikas A, Shakir S, Gafoor S, Omran H, Berti S, Santoro G, et al. Left atrial appendage occlusion for stroke prevention in atrial fibrillation: Multicentre experience with the AMPLATZER Cardiac Plug. EuroIntervention. 2016 Feb 1;11(10):1170–9.

50. Bösche LI, Afshari F, Schöne D, Ewers A, Mügge A, Gotzmann M. Initial Experience With Novel Oral Anticoagulants During the First 45 Days After  Left Atrial Appendage Closure With the Watchman Device. Clinical cardiology. 2015 Dec;38(12):720–4.

51. Wiebe J, Franke J, Lehn K, Hofmann I, Vaskelyte L, Bertog S, et al. Percutaneous Left Atrial Appendage Closure With the Watchman Device: Long-Term  Results Up to 5 Years. JACC Cardiovascular interventions. 2015 Dec;8(15):1915–21.

52. Meincke F, Schmidt-Salzmann M, Kreidel F, Kuck KH, Bergmann MW. New technical and anticoagulation aspects for left atrial appendage closure using  the WATCHMAN® device in patients not taking warfarin. EuroIntervention : journal of EuroPCR in collaboration with the Working Group on  Interventional Cardiology of the European Society of Cardiology. 2013 Aug;9(4):463–8.
